# Supplementary material for: Role of vector phenotypic plasticity in disease transmission as illustrated by the spread of dengue virus by Aedes albopictus
Source: Nat Commun. 2024 Sep 7;15:7823. doi: 10.1038/s41467-024-52144-5 (PMC11379831; doi:10.1038/s41467-024-52144-5)
Supplement: Supplementary file 1 — Supplementary Information [file 41467_2024_52144_MOESM1_ESM.pdf]

Supplementary Information: Role of vector phenotypic plasticity in disease transmission as illustrated by the spread of dengue virus by *Aedes albopictus*

Dominic P. Brass[1,2], Christina A. Cobbold[3], Bethan V. Purse[1], David A. Ewing[4], Amanda Callaghan[2], and Steven M. White[1]

[1] *UK Centre for Ecology & Hydrology, Benson Lane, Wallingford, Oxfordshire, OX10 8BB, UK*
[2] *Ecology and Evolutionary Biology, School of Biological Sciences, University of Reading, Reading, UK*
[3] *School of Mathematics and Statistics, College of Science and Engineering, University of Glasgow, University Place, Glasgow, UK*
[4] *Biomathematics and Statistics Scotland, Edinburgh, UK*

Contents

S. 1. Validations of the population dynamical model 3
S. 1.1. Example of model predictions 4
S. 1.1.1. Environmental variables 4
S. 1.1.2. Eggs 5
S. 1.1.3. Larvae 7
S. 1.1.4. Pupae 8
S. 1.1.5. Adults 9
S. 1.1.6. Infected adults 10
S. 1.1.7. Humans 11
S. 1.2. Europe 12
S. 1.2.1. Emilia-Romagna, Italy 12
S. 1.2.2. Rome, Italy 13
S. 1.2.3. Como, Italy 14
S. 1.2.4. Catania, Italy 15
S. 1.2.5. Trentino, Italy 16
S. 1.2.6. Cosenza, Italy 17
S. 1.2.7. Cagnes-sur-Mer, France 18
S. 1.2.8. Podgorica, Montenegro 19
S. 1.2.9. Zambelici, Montenegro 20
S. 1.2.10. Budva, Montenegro 21
S. 1.2.11. Ludwigshafen, Germany 22
S. 1.2.12. Freiburg im Breisgau, Germany 23
S. 1.2.13. Irun, Spain 24
S. 1.2.14. Baix Llobregat, Spain 25
S. 1.2.15. Split, Croatia 26
S. 1.2.16. Loule, Portugal 27
S. 1.2.17. Athens, Greece 28
S. 1.3. America 29
S. 1.3.1. Lake Charles, Louisiana 29
S. 1.3.2. New Orleans, Louisiana 30
S. 1.3.3. Fort Worth, Texas 31
S. 1.3.4. Lubbock, Texas 32
S. 1.3.5. Stratford, Connecticut 33
S. 1.3.6. Monmouth, New Jersey 34
S. 1.3.7. Charlotte, North Carolina 35
S. 1.3.8. Raleigh, North Carolina 36
S. 1.3.9. Asheville, North Carolina 37
S. 1.3.10. Greenville, North Carolina 38

S. 1.3.11. Indianapolis, Indiana . . . 39
S. 1.3.12. Washington, D.C. . . . 40
S. 1.3.13. Colombus, Ohio . . . 41
S. 1.3.14. Suffolk, Virginia . . . 42
S. 1.3.15. Santa Rosa Beach, Florida . . . 43
S. 1.4. Asia . . . 44
S. 1.4.1. Naha, Japan . . . 44
S. 1.4.2. Nagasaki, Japan . . . 45
S. 1.4.3. Tokyo, Japan . . . 46
S. 1.4.4. Guangzhou, China . . . 47
S. 1.4.5. Suwon, South Korea . . . 48
S. 1.5. Africa . . . 49
S. 1.5.1. La Reunion . . . 49

S. 2. Validations of the SEIR model 50
S. 2.1. Cagnes-sur-Mer . . . 51
S. 2.2. Guangzhou . . . 52
S. 2.3. Tokyo . . . 53
S. 2.4. Reunion . . . 54
S. 2.5. Hawai'i . . . 55

S. 3. Non-plastic and unstructured models 56
S. 3.1. Constant wing length models . . . 56
S. 3.2. Models with instantaneously varying wing length . . . 59
S. 3.3. Models with variable average wing length but without population structure . . . 60

S. 4. The basic reproduction number, R_0 62
S. 4.1. Comparison to previous R_0 equations . . . 62
S. 4.2. R_t at constant temperatures . . . 64

S. 5. Suitability maps 66
S. 5.1. First day of adult activity . . . 67
S. 5.2. Last day of adult activity . . . 68
S. 5.3. Duration of adult activity . . . 69
S. 5.4. Average adult density over active season . . . 70
S. 5.5. Average adult density over whole year . . . 71
S. 5.6. Maximum adult density . . . 72
S. 5.7. Time of maximum adult density . . . 73
S. 5.8. Average trait . . . 74
S. 5.9. Total dengue cases . . . 75
S. 5.10. First time $R_t > 1$ . . . 76
S. 5.11. Last time $R_t > 1$ . . . 77
S. 5.12. Duration of time for which $R_t > 1$ . . . 78
S. 5.13. Humidity layer . . . 79

S. 6. Glossary and parameter values 80

Bibliography 81

S. 1. Validations of the population dynamical model

Here we perform validations comparing our predictions of the population and trait dynamics of *Ae. albopictus* to data observed in field studies using R^2 to quantify goodness of fit. As both the field studies and our model only reflect relative abundance we uniformly apply a scaling factor to our predictions. Further, as the precise time of sampling is often ambiguous we also allow the uniform time-shift of observation by up to half of the interval between samples. We select the scaling factor and time-shift pair by trialling a range of possible values and retaining the pair that produces the maximum R^2 value. For datasets with multiple years of observations we use a single scaling factor and time shift for the entire dataset, preserving comparisons of relative density and fit between years. For each validation we report both the scaling factor and the time shift in addition to the value of R^2.

In many locations we wish to compare our predictions of the number of eggs within the population to the number of eggs that have accumulated in ovitraps over a number of days. We therefore convert our predictions of the number of eggs present in the population at time t to the number of eggs that would be collected in an ovitrap given the dynamics of the population we predict, which we term oviposition activity. For a study with a sampling period of τ_{sample} the number of individuals that would collect in an ovitrap sampled at time t is given by

$$OA(t) = \begin{cases} \sum_{i=1}^{\tau_{sample}} \sum_{j=1}^{m} \frac{q_j A_j(i)}{G(i)} - \frac{q_j A_j(i-\tau_{E_\gamma}(i))}{G(i-\tau_{E_\gamma}(i))} \text{ if } \tau_{E_\gamma}(i) < \tau_{sample}, \\ \sum_{i=1}^{\tau_{sample}} \sum_{j=1}^{m} \frac{q_j A_j(i)}{G(i)} - \frac{q_j A_j(i-\tau_{sample})}{G(i-\tau_{sample}))}, \text{ otherwise.} \end{cases} \quad \text{(S.E. 1.)}$$

S. 1.1. Example of model predictions

To demonstrate how the model functions we consider in detail the population dynamics and disease dynamics predicted by the model in a single location, in this case Rimini, Italy. This is a temperate location in the species invaded range where in 2007 the population of *Ae. albopictus* established in this region facilitated an outbreak of Chikungunya [1]. The model is simulated with 64 environmental classes, a number lower than the 200 used in the rest of the outputs, chosen to allow the reader to see more clearly how phenotypic plasticity acts. A single infected individual is introduced on 24th of May each year into a susceptible population of density 2088 people per km^2.

S. 1.1.1. Environmental variables

To simulate the model in this region we input the observed temperature, precipitation, evaporation, and the latitude of Rimini (Supplementary Figure 1). Using these quantities we simulate the model, initiating the dynamics in the year 2006 to allow a burn-in period of two years for the population dynamics, preventing the chosen initial conditions from altering the dynamics in the prediction year. These environmental variables are used to determine how the food available to larvae varies seasonally, as shown in Supplementary Figure 1D.

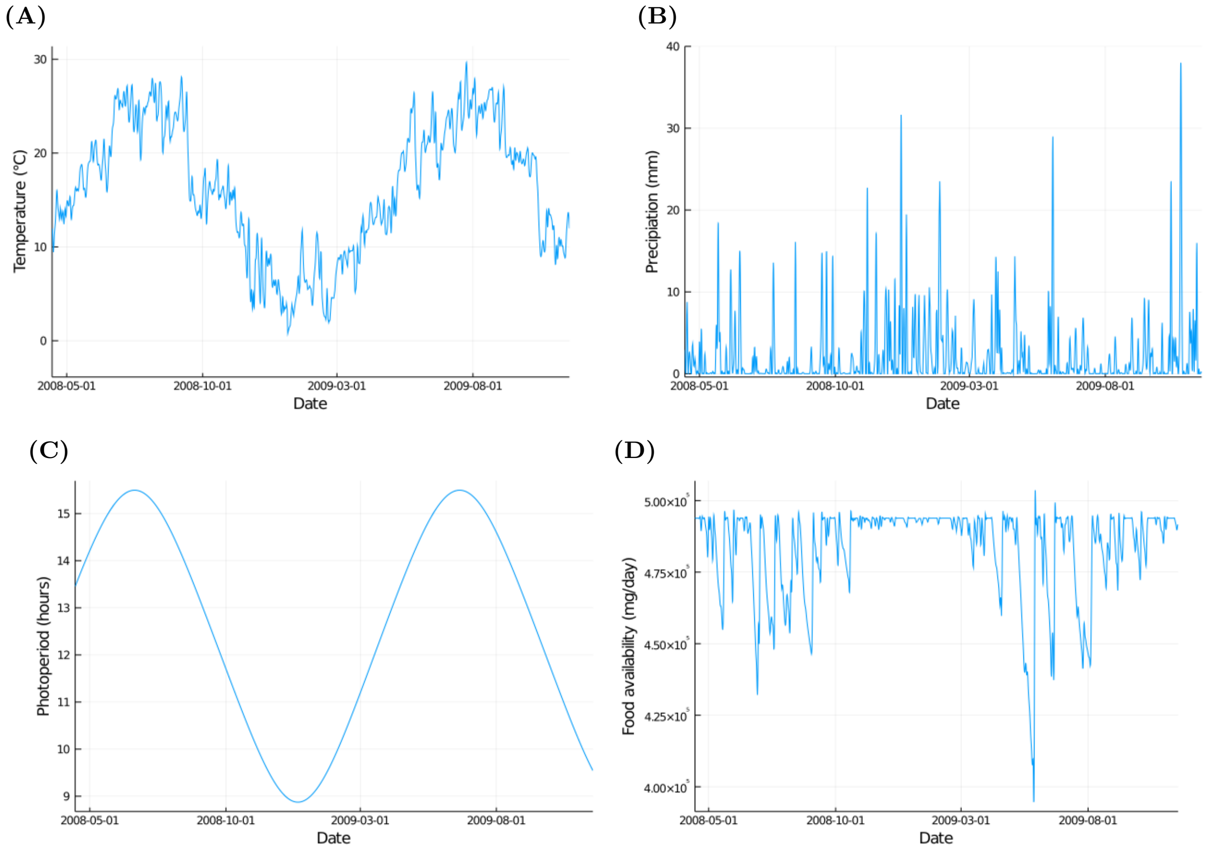

Supplementary Figure 1: The environmental variables used to simulate the model in Rimini. Source data are provided as a Source Data file. **(A)** Temperature. **(B)** Precipitation. **(C)** Photoperiod. **(D)** Total food produced in the developmental habitat per day.

S. 1.1.2. Eggs

In Supplementary Figure 2A the dynamics of the various egg classes as predicted by the model in Rimini are shown. Active eggs are produced throughout the summer and autumn, before falling temperatures and photoperiod trigger the production of diapausing eggs. As the temperature warms the duration of time taken for active eggs to develop decreases and a greater proportion survive through to the larval stage (Supplementary Figures 2B and 2C). Diapause eggs are produced in a short period of time before cold temperatures cause any remaining adults to die off, and these dormant eggs persist throughout the winter months experiencing a constant low level of mortality. In this region the temperatures never drop low enough to reach the lower thermal limit on diapause egg survival, allowing a sufficient number of eggs to survive through the winter to allow the population to persist in this region between years. When the critical photoperiod and temperature are reached these diapause eggs hatch into larvae and allow the life-cycle to continue. Quiescent eggs are produced in low-levels throughout the year whenever the water level of the container habitat begins to decrease, immediately hatching when inundated.

(A)

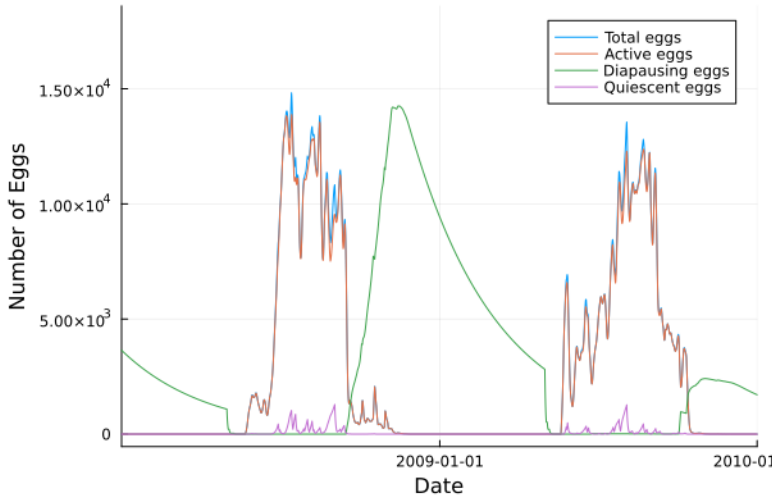

(B)

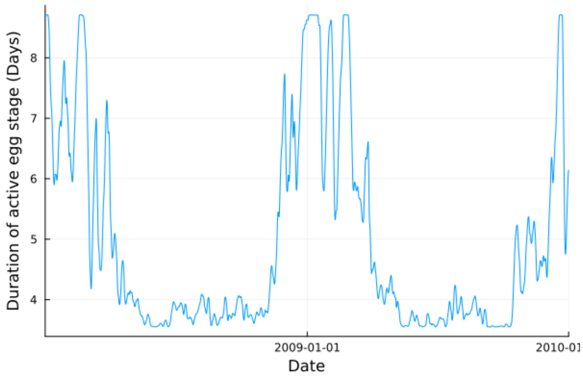

(C)

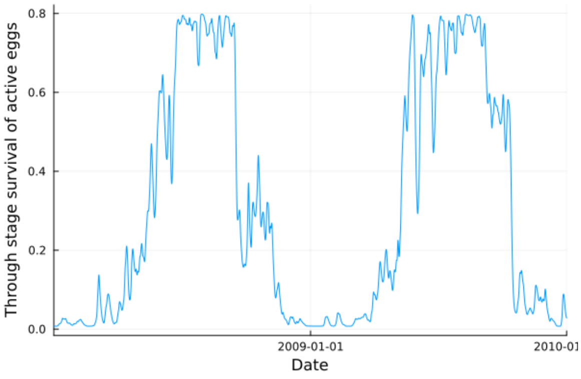

Supplementary Figure 2: Outputs from the model when simulated in Rimini, Italy for the egg stages. Source data are provided as a Source Data file. **(A)** The number of eggs predicted by the model. The blue line is the total number of eggs across all classes, the orange line is the number of active eggs, the green line represents the number of diapausing eggs, and the purple line the number of quiescent eggs. **(B)** The duration of the active egg stage, τ_{E_γ}. **(C)** The proportion of eggs that survive through the egg class, S_{E_γ}.

To compare the number of eggs predicted by the model to the number of eggs collected in ovitraps in Rimini we apply equation S.E. 1. to the predicted population dynamics to produce a prediction of oviposition activity (Supplementary Figure 3). Our predictions of total egg number in Supplementary Figure 2A and the calculated oviposition activity display different dynamics in Supplementary Figure 3. This occurs due to the varying duration of the egg stage, temperature induced fluctuations in the length of the gonotrophic cycle, and variation in adult trait, leading to different numbers of eggs accumulating in the simulated habitat than are detected in the regularly sampled ovitrap. To make the comparison between the model prediction and field observation we apply a scaling factor and time-shift to maximize R^2. The scaling factor applied is, $sf = 0.007$, and we

find that no time-shift is required and so $ts = 0$. Under these transformations the model predicts the observed oviposition activity in Rimini in 2008 accurately with $R^2 = 0.94$.

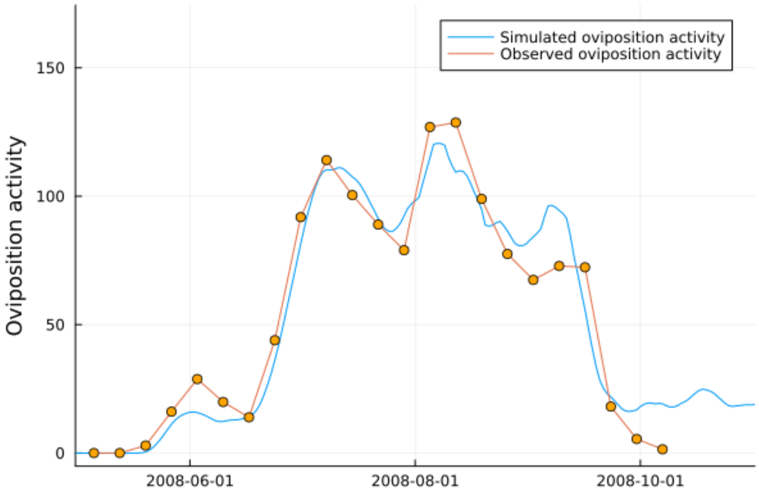

Supplementary Figure 3: A comparison of the predictions of the model (blue line) and field data (orange points and line) in the Rimini, Italy region in the year 2008

S. 1.1.3. Larvae

In Supplementary Figure 4 the abundance and traits that are predicted for larval mosquitoes in Rimini are shown. The initial generation of larvae emerges due to the release of dormant eggs from diapause resulting in the synchronised spike in larval density observed at the start of the active season (Supplementary Figure 4A). After this initial generation of pharate larvae, larval numbers increase over the course of the year, eventually reaching densities unfavourable for successful development due to intense competition. This leads to low through stage-survivals towards the latter half of the year, which is compounded by the decrease in temperature that occurs over the same period.

(A)

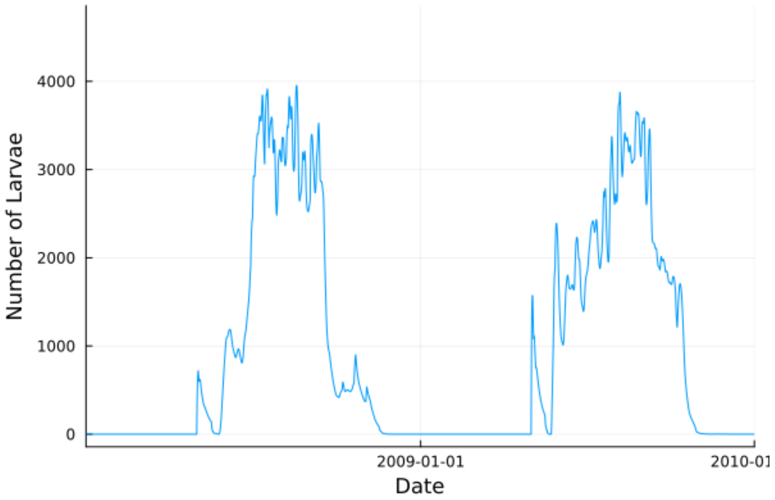

(B)

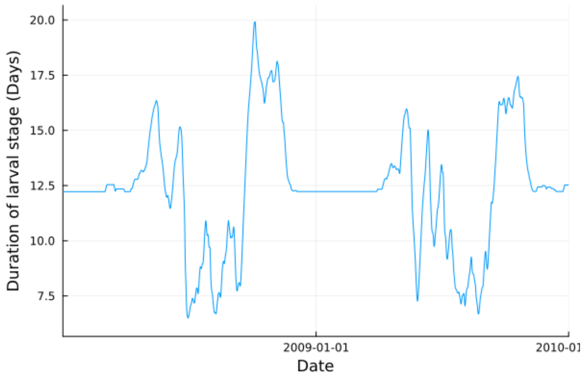

(C)

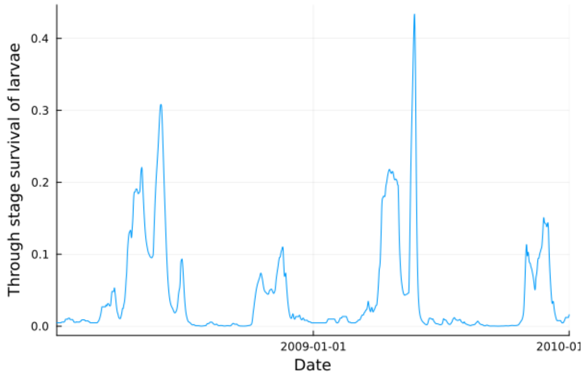

Supplementary Figure 4: Outputs from the model when simulated in Rimini, Italy for the larval stage. Source data are provided as a Source Data file. **(A)** The number of larvae predicted by the model. **(B)** The duration of the larval stage, τ_L. **(C)** The proportion of larvaer that survive through the larval stage, S_L.

S. 1.1.4. Pupae

Supplementary Figure 5 shows our predictions of the trait dynamics of pupae. Although we do not explicitly represent pupae within the model through a state equation describing their abundance, they are implicitly modelled through the pupal traits of stage duration and survival which vary seasonally with temperature.

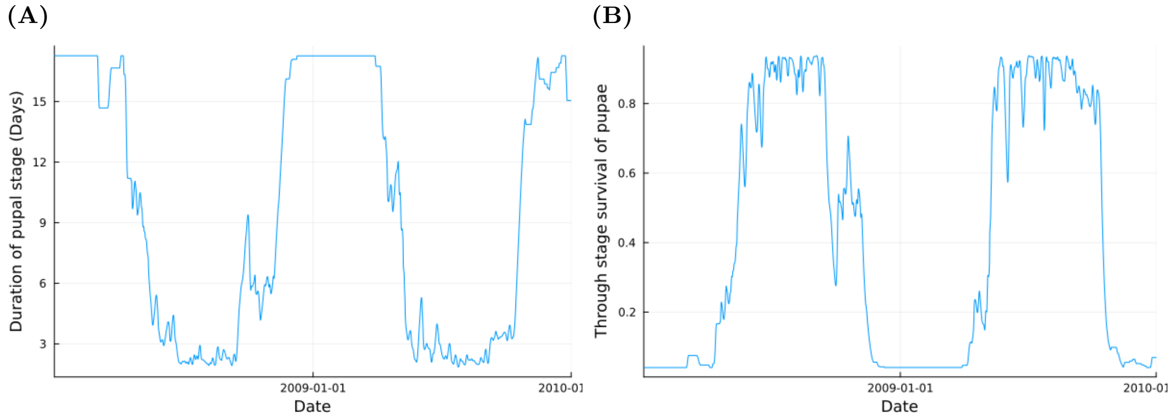

Supplementary Figure 5: Outputs from the model when simulated in Rimini, Italy for the pupal stage. Source data are provided as a Source Data file. **(A)** The duration of the pupal stage, τ_P. **(B)** The proportion of eggs that survive through the pupal stage, S_P.

S. 1.1.5. Adults

The model predictions of adult abundance track the same seasonal trends exhibited by eggs and larvae, increasing as temperatures increase through summer and then decreasing as temperatures decrease (Supplementary Figure 6A). In Supplementary Figure 6B we see that the initial generation of adults is large, expressing a wing length of around 3.3 mm, these are quickly outnumbered by subsequent generations of smaller and smaller larvae that developed under warmer conditions, experiencing higher competition and by summer the most abundant phenotype expresses a wing length of around 2.5 mm. However, we see that during this summer peak there is a defined trait structure that can be observed in the mosquito population due to the persistence of larger individuals that emerged earlier in the year. Indeed, we can see that the initial cohort of large individuals still persists. As temperatures decrease there is a small final generation of large adults before the adult population completely dies off due to being subject to unfavourable temperatures.

(A)

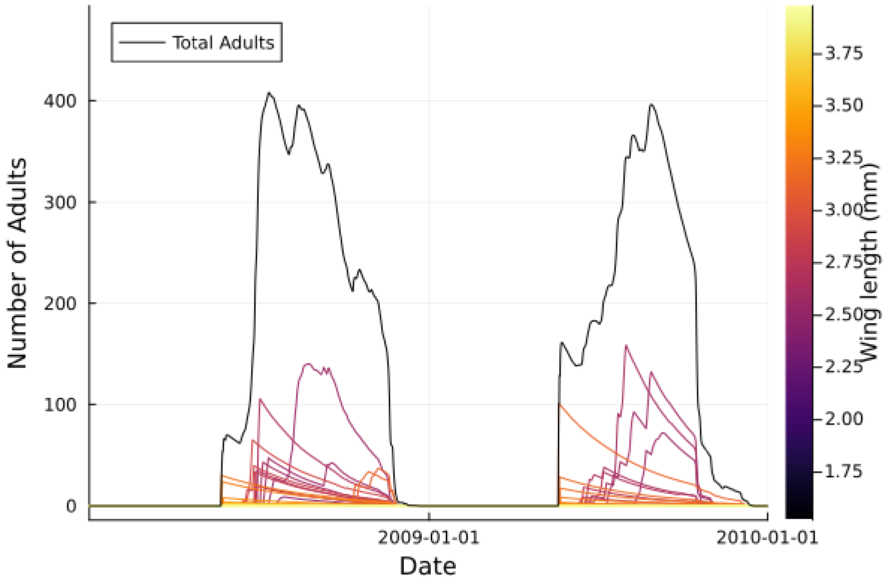

(B)

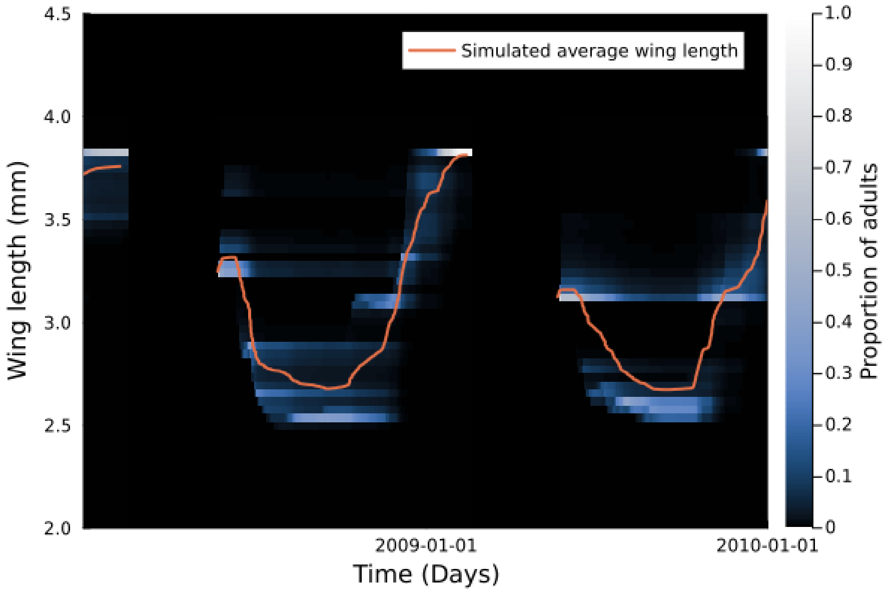

Supplementary Figure 6: Outputs from the model when simulated in Rimini, Italy for the adult stage. Source data are provided as a Source Data file. **(A)** The number of adults adults (black line), partitioned into environmental classes according to their wing length (coloured lines). **(B)** The model prediction of wing lengths expressed by adults in Rimini, the orange line is the population average trait and each blue line represents the proportion of adults within the population that express the corresponding wing length.

S. 1.1.6. Infected adults

The model's predictions of the number of infected adults and their trait distribution following the introduction of a single infected individual on the 24th of May each year (Supplementary Figure 7). We can see that the model predicts a $2-3$ times as many infected adults in 2008 than in 2009 despite a minimal difference in overall adult abundance.

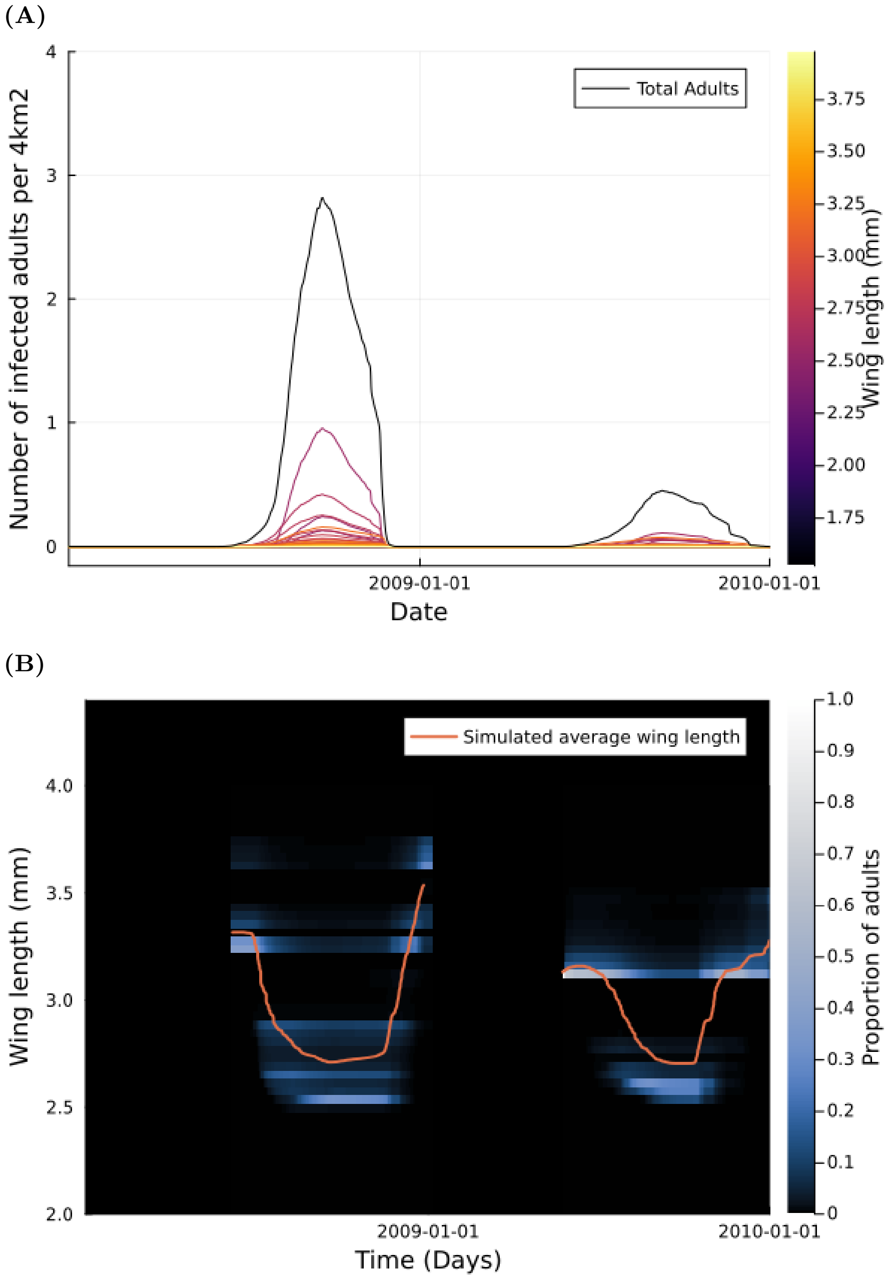

Supplementary Figure 7: Outputs from the model when simulated in Rimini, Italy for the infected adult stage. **(A)** Total number of infected adults across all environmental classes (black line) and infected adults in individual environmental classes as defined by wing length (coloured lines). **(B)** The model prediction of the wing lengths expressed by infected adults in Rimini, the orange line is the population average trait and each blue line represents the proportion of infected adults within the population that express the corresponding wing length.

S.1.1.7. Humans

Our predictions of the infection dynamics of the human population and the daily rate of infection are shown in Supplementary Figure 8. Broadly speaking Rimini during the period simulated was unsuitable for the transmission of dengue by *Ae. albopictus* and we can see that only a very small number of individuals become infected each year from the introduction of a single infected individual. This unsuitability is reflected in the daily infection rate which we can see is dominated by the introduction of the infectious individual at the start of the outbreak, and in the fact that dengue does not persist from year to year.

(A)

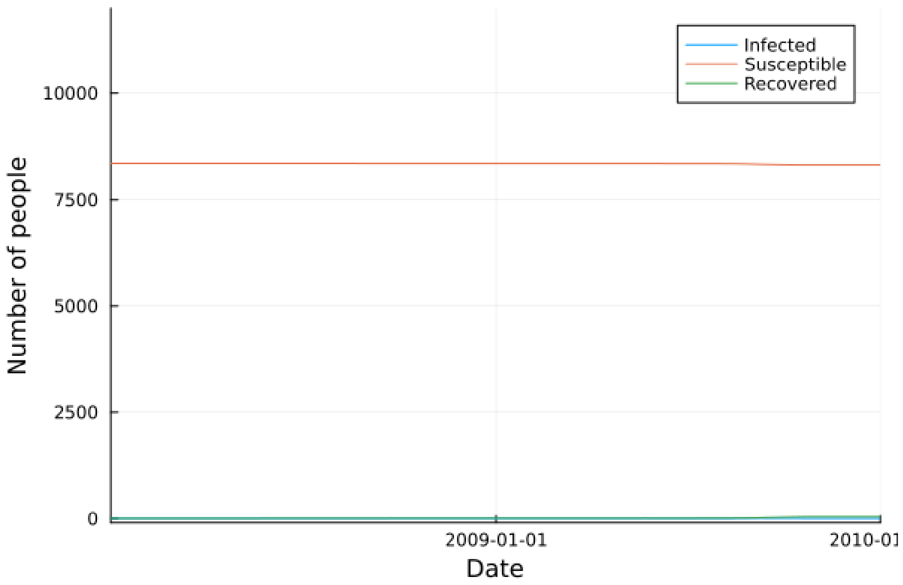

(B)

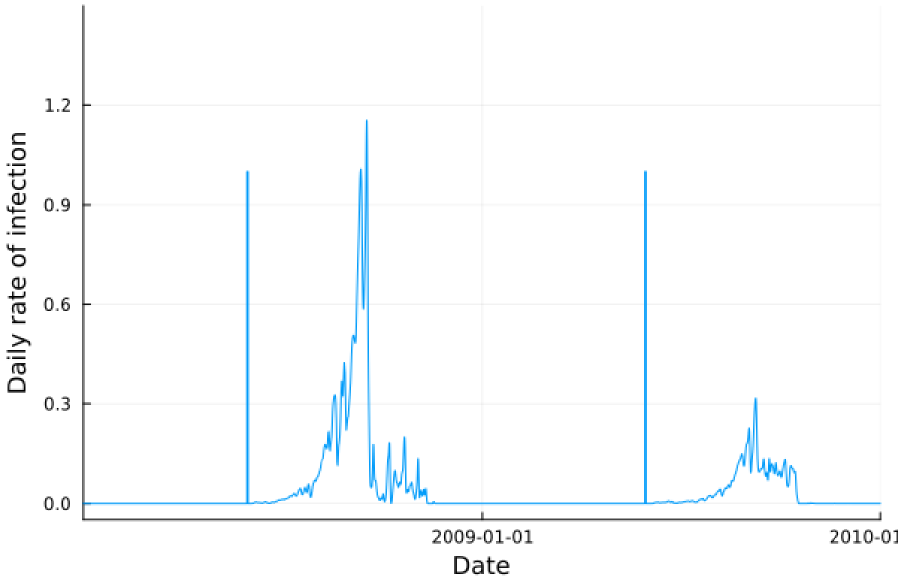

Supplementary Figure 8: Outputs from the model when simulated in Rimini, Italy for the human population. Source data are provided as a Source Data file. **(A)** The number of humans in each infection class. The blue line is the number of infected humans, the orange line is the number of susceptible humans and the green line is the number of recovered humans. **(B)** The model prediction of the number of humans that become infected each day.

S. 1.2. Europe

S. 1.2.1. Emilia-Romagna, Italy

A network of ovitraps is maintained throughout the Emilia-Romagna region of Italy and the observations from these traps have been published in the years 2008, 2014, and 2015 [2, 3]. The sampling method used in 2008 was different to that used in $2014 - 2015$ and so although observations in 2014 and 2015 are directly comparable to each other they cannot be compared to observations from 2008. We predict the observed oviposition activity in 2008 accurately with $R^2 = 0.94$ (Supplementary Figure 9A) with a scaling factor of $sf = 0.007$ and adjusted time step of $ts = 0$ days. For the data for the combined years $2014 - 2015$ the same validation process yields $R^2 = 0.69$, $ts = 2$, $sf = 0.04$, and we are able to reproduce the difference in oviposition activity between the years (Supplementary Figure 9B).

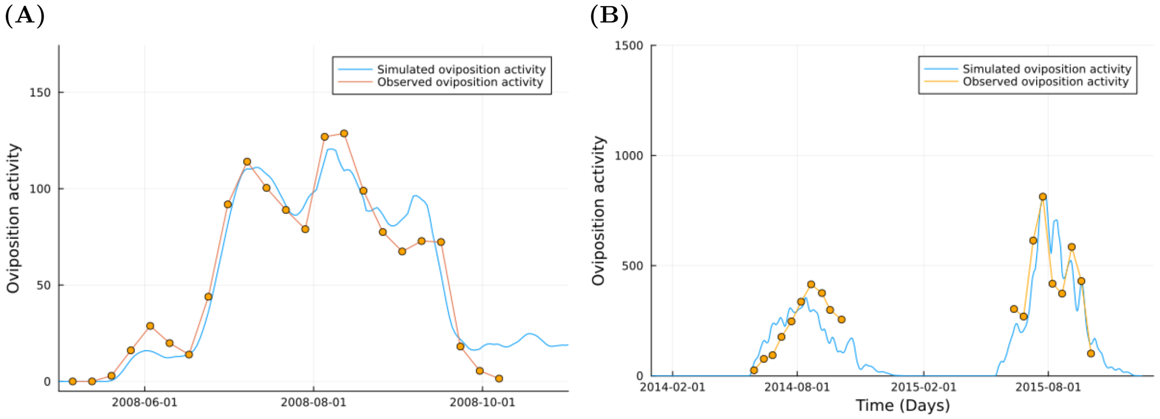

Supplementary Figure 9: A comparison of our predictions (blue line) and field data (orange points and line) in the Emilia-Romagna region in the years **(A)** 2008 **(B)** 2014-2015. Source data are provided as a Source Data file.

In addition to oviposition activity, a nuisance factor was estimated through human landing collections. To replicate this the biting rate as defined in the SEIR model is used along with the predictions of adult abundance to predict the number of bites produced per habitat per day, (i. e. $\sum_{j=1}^{m} b(t)A_j(t)$) and this is then compared to the field observations. As can be seen in Supplementary Figure 10 our prediction of number of bites per day follows the dynamics of the observed nuisance factor.

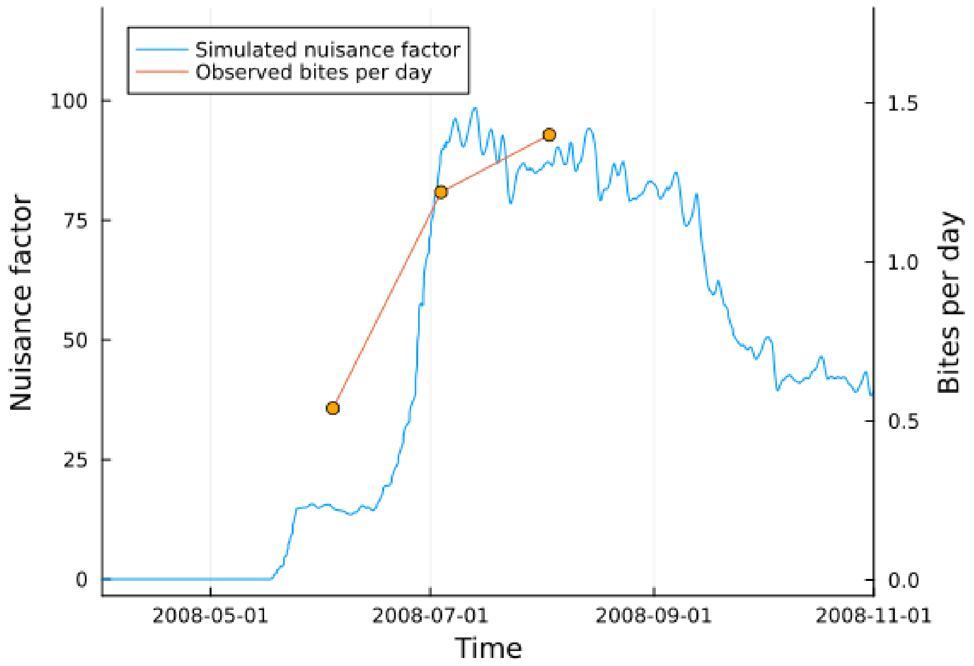

Supplementary Figure 10: A comparison of the model predictions of nuisance factor (blue line) and field data (orange points and line) in the Emilia-Romagna region in the year 2008. Source data are provided as a Source Data file.

S. 1.2.2. Rome, Italy

Toma et al. (2003) monitored the oviposition activity of *Aedes albopictus* in Rome, Italy in the year 2000 [4]. We predict the dynamics of this population with a fit of $R^2 = 0.83$, $sf = 0.0071$, $ts = 1$ (Supplementary Figure 11A). Manici et al. (2017) monitored the number of adult females in Rome, Italy during the year 2012 [5]. We predict the dynamics of this population with a fit of $R^2 = 0.43$, $sf = 0.0085$, $ts = -7$ (Supplementary Figure 11B).

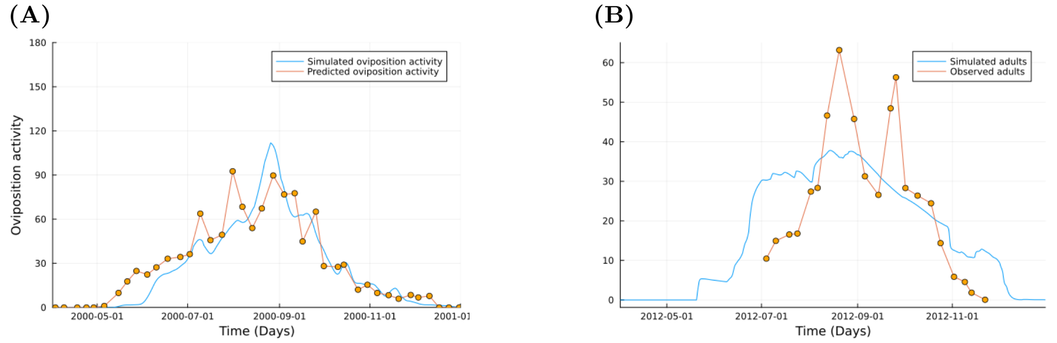

Supplementary Figure 11: A comparison of the model predictions (blue line) and field data (orange points and line) for Rome, Italy **(A)** of oviposition activity in the year 2000 **(B)** adult females in the year 2012. Source data are provided as a Source Data file.

S. 1.2.3. Como, Italy

Suter et al. (2016) monitored the oviposition activity of *Aedes albopictus* along the Swiss/Italian border in an intervention area and non-intervention area over two years [6]. We simulate the model in the non-intervention area in, Como, Italy and achieve a fit to field data of $R^2 = 0.72$, $sf = 0.024$, $ts = -2$ as shown in Supplementary Figure 12.

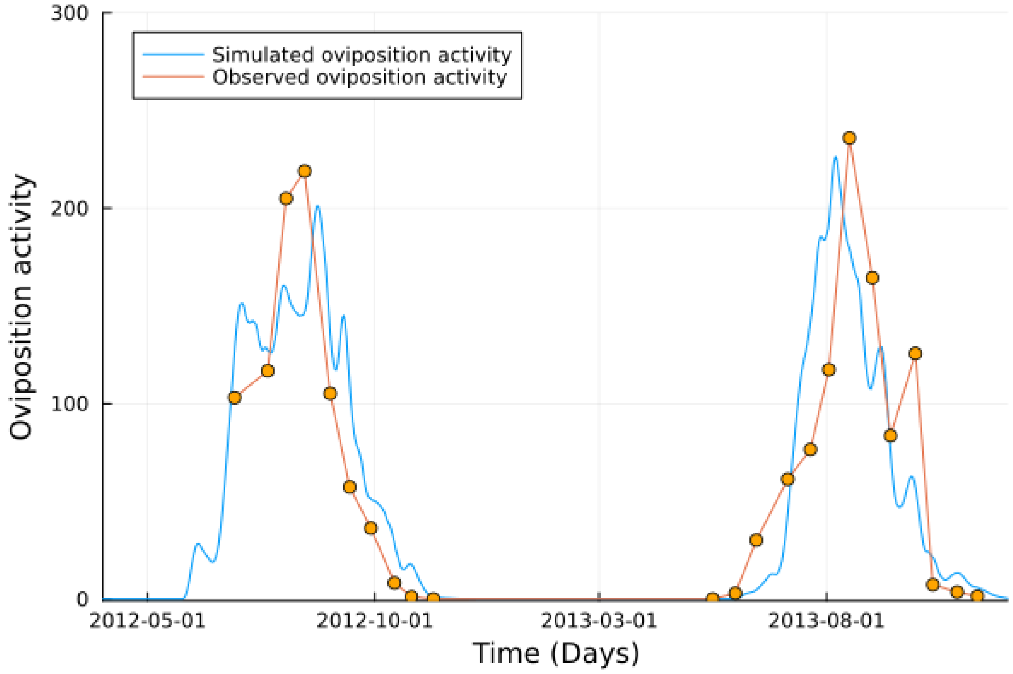

Supplementary Figure 12: A comparison of the model predictions (blue line) and field data (orange points and line) for Como, Italy for the years 2012 – 2013. Source data are provided as a Source Data file.

S. 1.2.4. Catania, Italy

Belle et al. (2018) monitored the oviposition activity in Catania, Italy in the years 2008 and 2013 [7]. We simulate the model in the citrus orchard achieving a fit of $R^2 = 0.86$, $sf = 0.26$, $ts = 21$ as shown in Supplementary Figure 13.

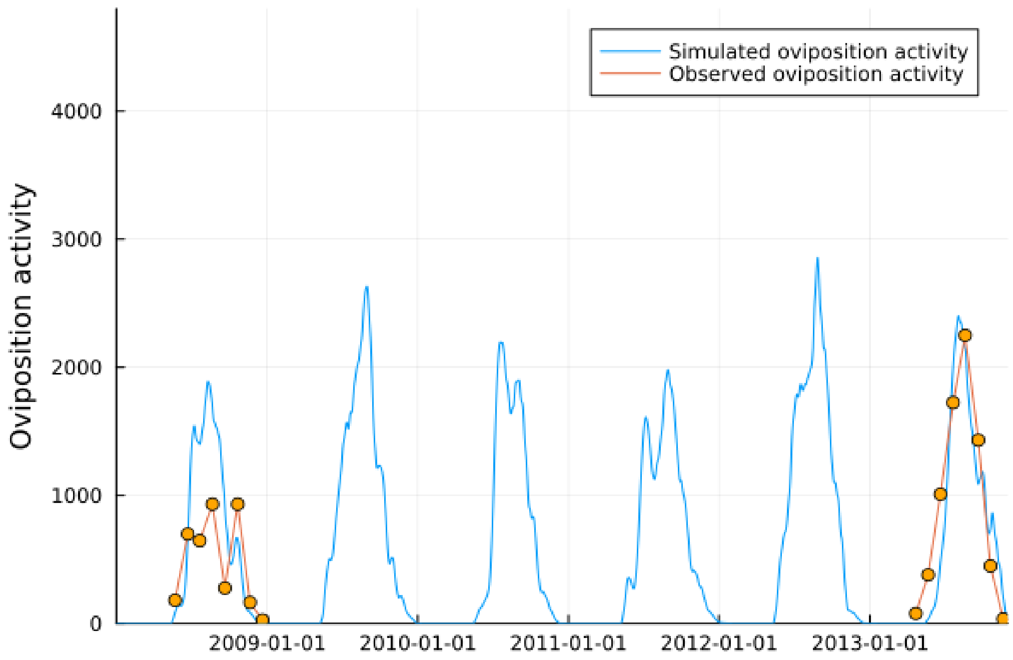

Supplementary Figure 13: A comparison of the model predictions (blue line) and field data (orange points and line) for Catiania, Italy for the years 2008 and 2013. Source data are provided as a Source Data file.

S. 1.2.5. Trentino, Italy

Roiz et al. (2011) monitored the adult activity in Arco in 2008 [8]. We predict this with $R^2 = 0.75$, $sf = 0.14$, $ts = 0$, as seen in Supplementary Figure 14A. Mairini et al. (2017) monitored adult activity in a range of towns in the region for the years $2014 - 2015$ [9]. To this data the model achieves a fit of $R^2 = 0.69$, $ts = -3$, $sf = 0.11$ as seen in Supplementary Figure 14B. Lencioni et al. monitored ovipostition activity in the years $2010 - 2020$ in Trento [10]. In Supplementary Figure 14C we achieve a good fit to the multi-year data set with $R^2 = 0.71$, $ts = -6$, $sf = 0.024$ and our predictions reflect the inter-annual differences in abundance.

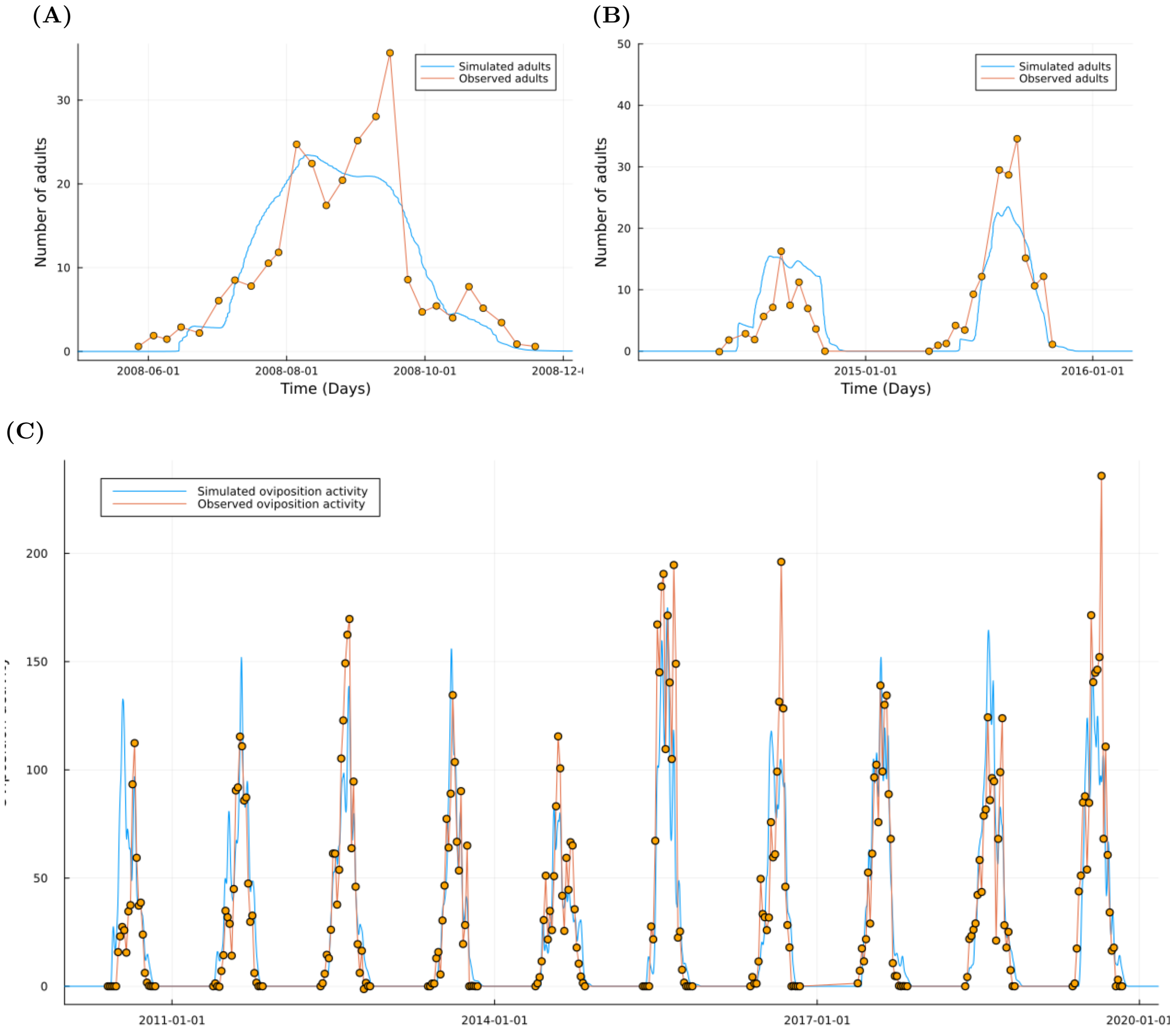

Supplementary Figure 14: A comparison of the model predictions (blue line) and field data (orange points and line) for data from the Trentino region. Source data are provided as a Source Data file. **(A)** Adult abundance in 2008. **(B)** Adult abundance in $2014 - 2015$. **(C)** Oviposition activity in $2010 - 2020$.

S. 1.2.6. Cosenza, Italy

Bonnacci et al. (2015) observed the oviposition activity of *Aedes albopictus* in Cosenza, Italy in 2013 [11]. It is unclear when exactly the observations were made as each data point corresponds to a week of the study rather than day of the year. To compare field observations with the models predictions it is therefore necessary to use contextual inferences, but this could be inaccurate by several weeks. The model fit is $R^2 = 0.21$, $sf = 0.077$, $ts = 10$ (Supplementary Figure 15).

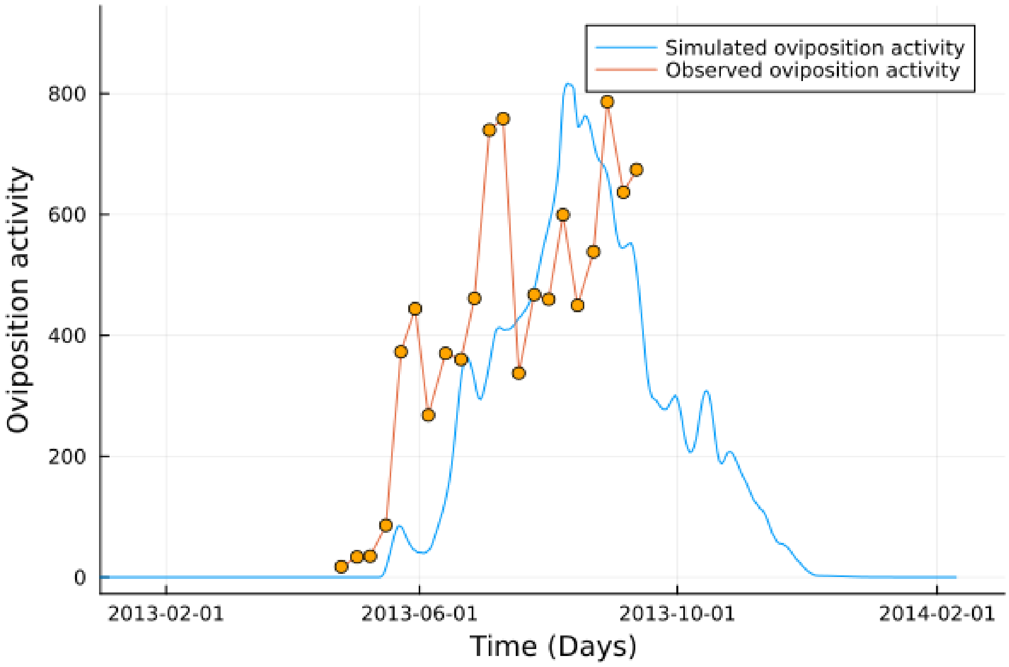

Supplementary Figure 15: The predicted oviposition activity of *Ae. albopictus* in Cosenza, Italy compared to oviposition data from Bonacci et al. (2015). Source data are provided as a Source Data file.

S. 1.2.7. Cagnes-sur-Mer, France

Lacour et al. (2015) monitored the oviposition activity of *Aedes albopictus* in Cagnes-sur-Mer, France over the years 2011 – 2013 [12]. The dynamics of this population are adequately predicted over multiple years with $R^2 = 0.63$, $sf = 0.015$, $ts = 5$ (Supplementary Figure 16).

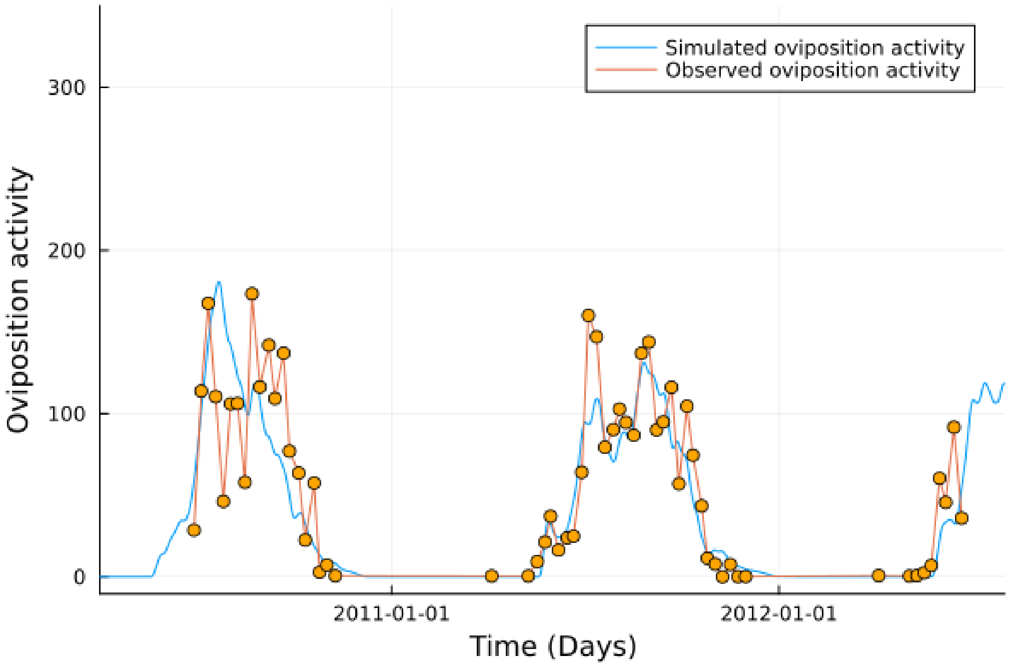

Supplementary Figure 16: A comparison of the model predictions (blue line) and field data (orange points and line) for Cagnes-sur-Mer, France in the years 2010 – 2012. Source data are provided as a Source Data file.

S. 1.2.8. Podgorica, Montenegro

Oviposition activity was monitored in Podgorica, Montenegro in 2013 [13]. This data was obtained through communication with Igor Pajović. We achieve a fit of $R^2 = 0.58$, $ts = 8$, $sf = 0.0085$ (Supplementary Figure 17).

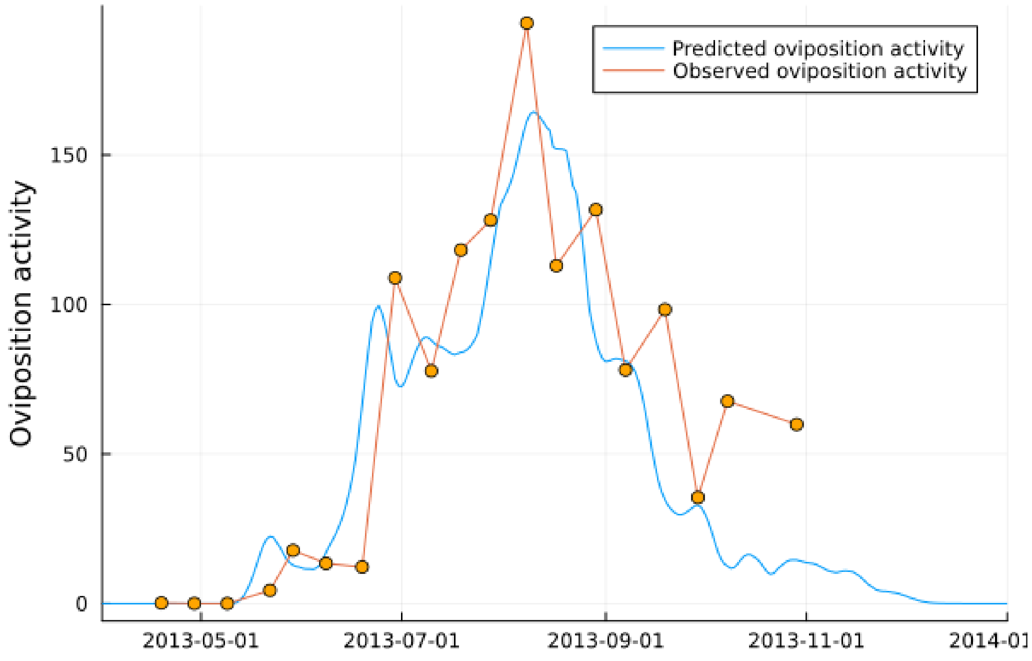

Supplementary Figure 17: A comparison of the model predictions (blue line) and field data (orange points and line) for Podgorica, Montenegro for the year 2013. Source data are provided as a Source Data file.

S. 1.2.9. Zambelici, Montenegro

Oviposition activity was monitored in Zambelici, Montenegro in the years 2018 − 2019 [13]. This data was obtained through communication with Igor Pajović. In Supplementary Figure 18 it can be observed that the we predict the data with fit $R^2 = 0.48$, $ts = 1$, $sf = 0.015$.

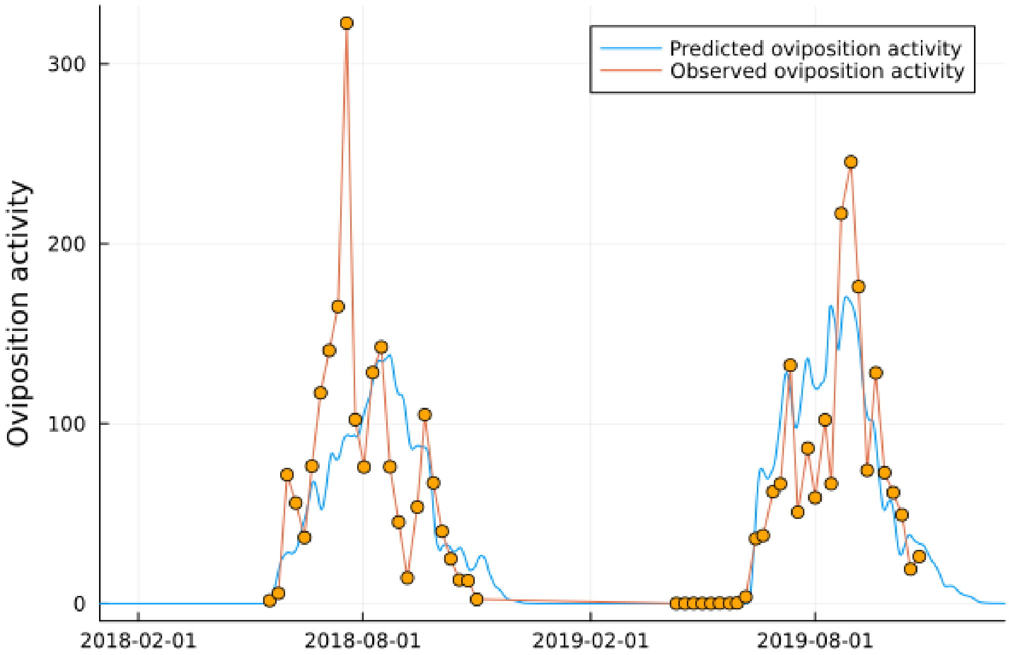

Supplementary Figure 18: A comparison of the model predictions (blue line) and field data (orange points and line) for Zambelici, Montenegro over the years 2018 − 2019. Source data are provided as a Source Data file.

S. 1.2.10. Budva, Montenegro

Oviposition activity was monitored in Budva, Montenegro in 2012 [13]. This data was obtained through communication with Igor Pajović. We predict the field data with fit $R^2 = 0.82$, $ts = -1$, $sf = 0.014$ as shown in Supplementary Figure 19.

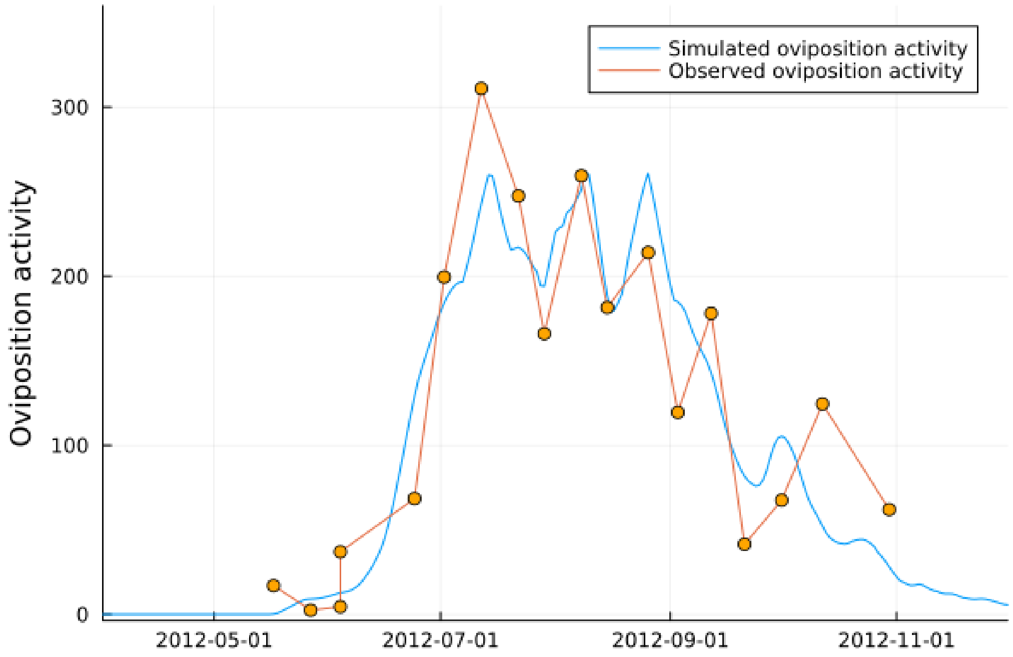

Supplementary Figure 19: A comparison of the model predictions (blue line) and field data (orange points and line) for Budva, Montenegro in 2012. Source data are provided as a Source Data file.

S. 1.2.11. Ludwigshafen, Germany

Becker et al. (2022) monitored oviposition activity in Ludwigshafen in the year 2020 [14]. In Supplementary Figure 20 we compare the model predictions to the data with a fit of, $R^2 = 0.71$, $ts = -4$, $sf = 0.024$.

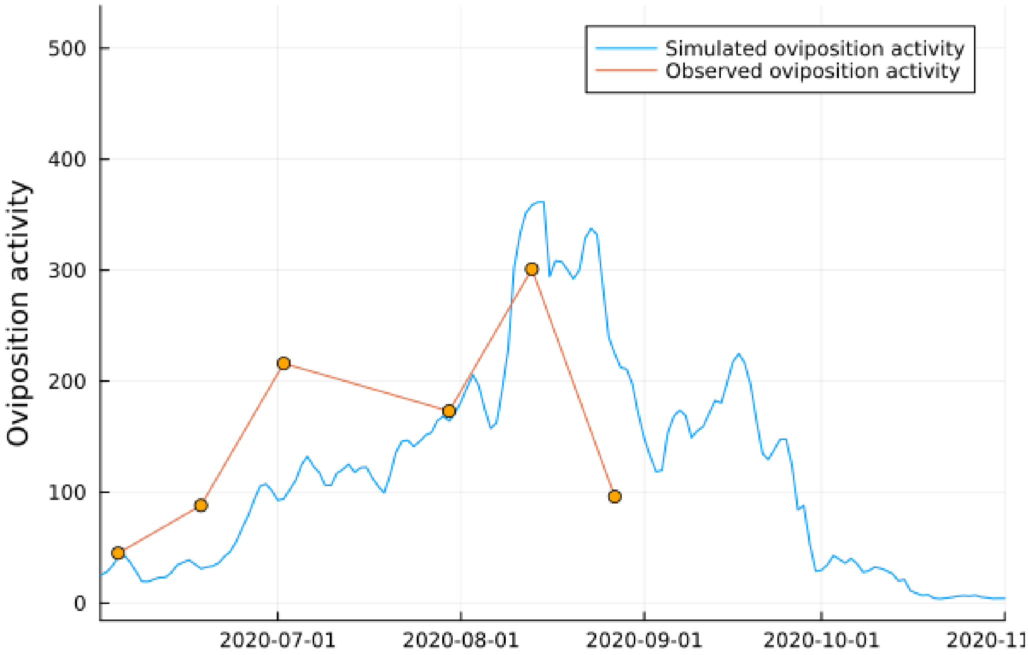

Supplementary Figure 20: A comparison of the predictions of the model (blue line) with the field data (orange points and line) for Ludwigshafen, Germany, for the year 2020. Source data are provided as a Source Data file.

S. 1.2.12. Freiburg im Breisgau, Germany

Becker et al. (2022) monitored oviposition activity in Freiburg in Breisgau in the year 2020, in an area where vector control had been implemented and in an area with no vector control. In Supplementary Figure 21 we compare our predictions to the data from the non-intervention area and find a good agreement, $R^2 = 0.98$, $ts = 2$, $sf = 0.146$.

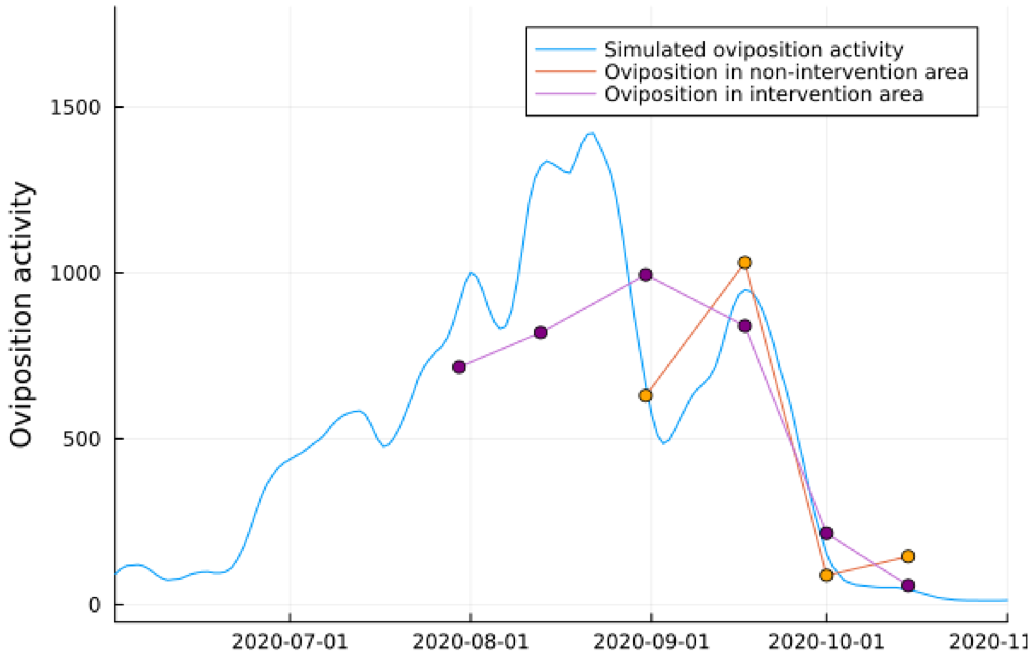

Supplementary Figure 21: A comparison of the model predictions (blue line) and field data (orange points and line) for Freiburg im Breisgau, Germany in 2020. Source data are provided as a Source Data file.

S. 1.2.13. Irun, Spain

Goiri et al. (2020) monitored oviposition activity in Northern Spain over the years 2013 − 2018, with *Ae. albopictus* being first detected in the region, Irun, in 2015 [15]. This population was actively invading the region at the time of the study and there were trials of different controls methods concurrent with the sampling. These control programmes were "non-unified" and "barely effective" and it is likely the control efforts coincided with continuous introduction events occurring throughout this period [15]. In Supplementary Figure 22 we see that the model predictions do not consistently match the observed field data, with the model only achieving a fit of $R^2 = 0.16$, $ts = -14$, $sf = 0.01$. In Goiri et al. it is observed that *Ae. albopictus* populations often take a few years to establish and we believe this combined with the control efforts in previous years explains the discrepancy between observed and predicted oviposition activity. The fit for only 2018 is $R^2 = 0.65$, $sf = 0.003$,$ts = -10$.

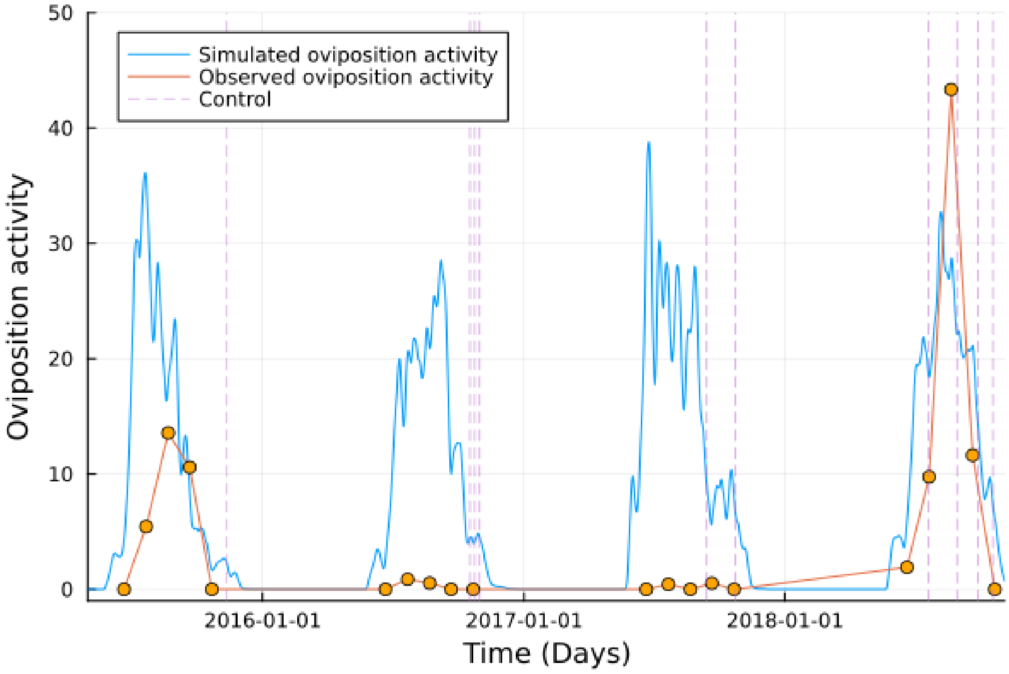

Supplementary Figure 22: A comparison of the model predictions (blue line) and field data (orange points and line) for Irun Spain over the years 2015 − 2018. The dashed purple lines indicate the times at which control efforts occurred. Source data are provided as a Source Data file.

S. 1.2.14. Baix Llobregat, Spain

Collantes et al. 2015 report the average oviposition activity observed for each week of the year in Llobregat, Spain for the years 2006 – 2014 [16]. We simulate the model for these years and calculate the average oviposition activity. In Supplementary Figure 23 we see the fit gives $R^2 = 0.82$, $sf = 0.009$,$ts = 1$.

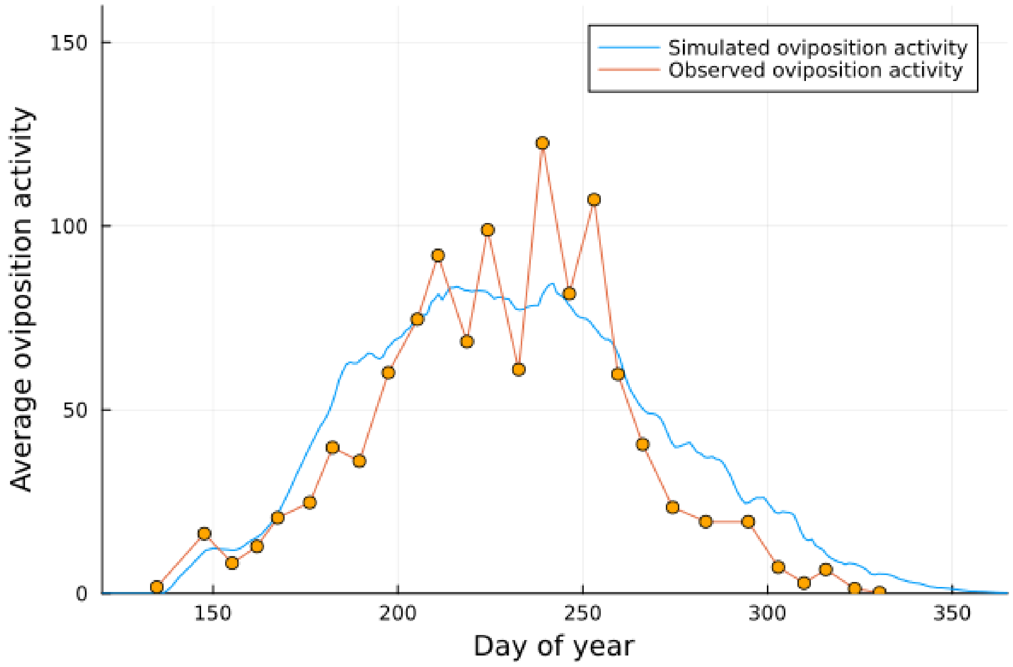

Supplementary Figure 23: A comparison of the weekly mean oviposition activity in the years 2006 – 2014 as we predict (blue line) and field data (orange points and line) for Baix Llobregat. Source data are provided as a Source Data file.

S. 1.2.15. Split, Croatia

Zitko and Merdic report oviposition data from Split, Croatia for the years 2009 – 2010 [17]. We predict the field data with a fit of $R^2 = 0.81$, $sf = 0.044$,$ts = -2$ as shown in Supplementary Figure 24.

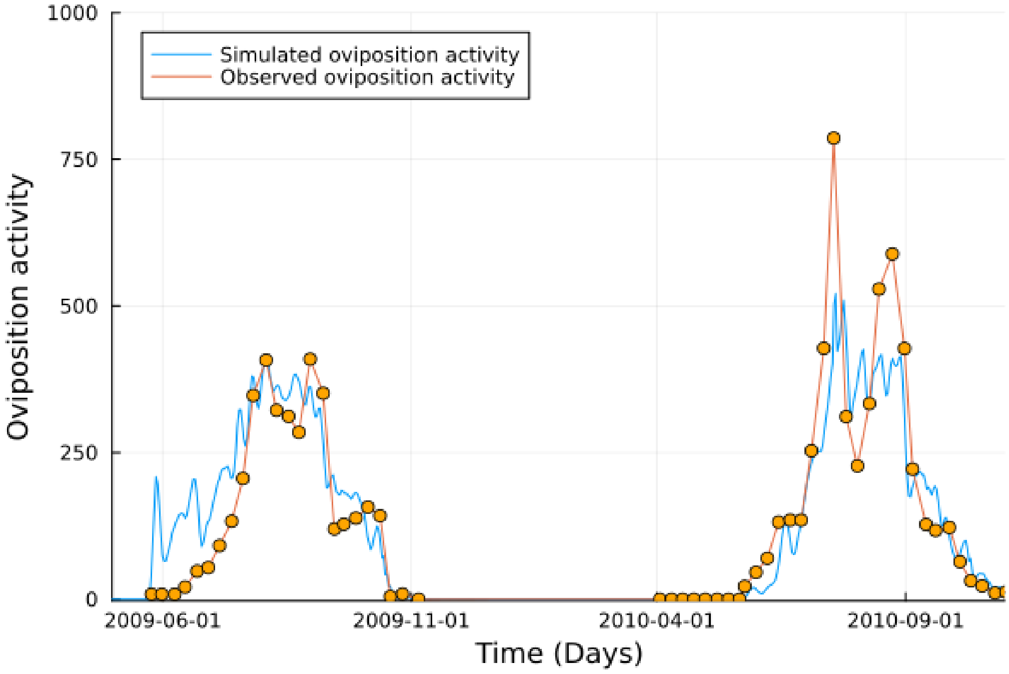

Supplementary Figure 24: A comparison of our predictions from the model (blue line) and from field data (orange points and line) for Split, Croatia in the years 2009 – 2010. Source data are provided as a Source Data file.

S. 1.2.16. Loule, Portugal

Osorio et al. (2020), monitored the oviposition activity and number of adults in Loule, Portugal in the year 2019 [18]. SDMs find this area is unsuitable for populations due to a lack of precipitation [19], but we find that a population can be maintained and predict a good fit to the observed field data for both oviposition activity in Supplementary Figure 25A, $R^2 = 0.82$, $sf = 0.66$, $ts = -15$, and adult numbers in Supplementary Figure 25B with, $R^2 = 0.66$, $sf = 0.22$, $ts = -14$.

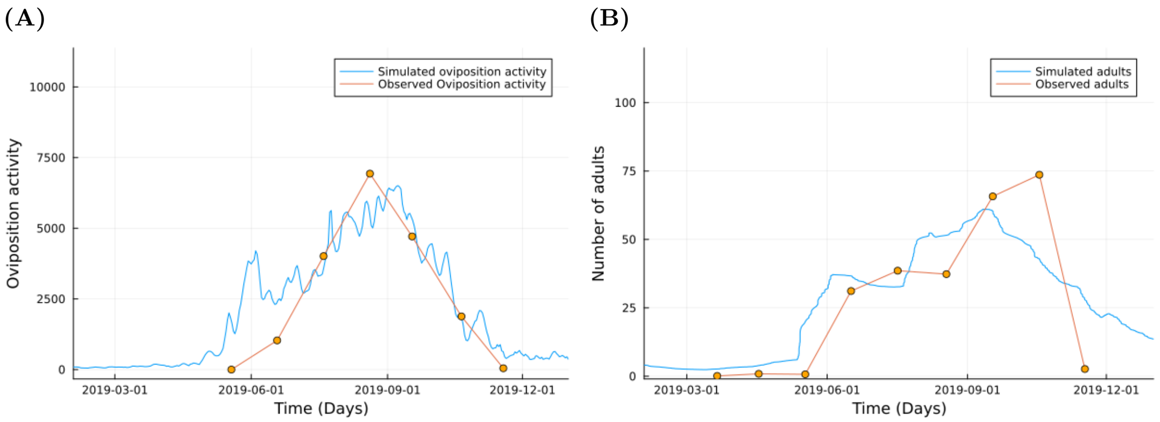

Supplementary Figure 25: A comparison of the model predictions (blue line) and field data (orange points and line) for Loule, Portugal in the years 2019. Source data are provided as a Source Data file. **(A)** Oviposition activity. **(B)** Adult numbers.

S. 1.2.17. Athens, Greece

Giatropoulos al. (2012) monitored oviposition activity in Athens, Greece in the years 2009 – 2010. Supplementary Figure 26 shows that the model fit is poor $R^2 = 0.09$, $sf = 0.014$, $ts = -4$. This may be explained by the lack of precipitation in this region making the simulated larval habitat unsuitable for the development of juveniles. It is likely, given the urban nature of Athens, that the field population observed here is at least in part reliant on sources of water that are not rain-fed.

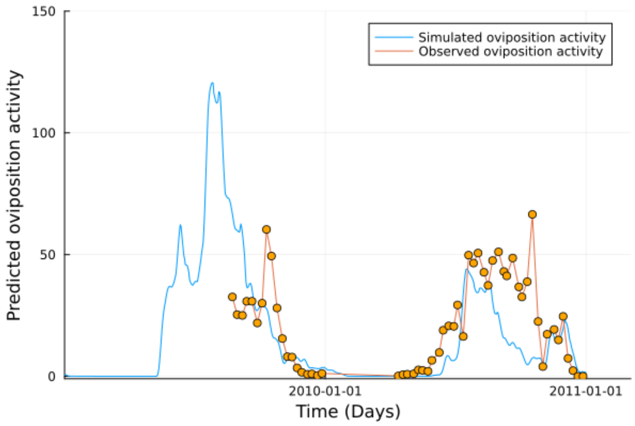

Supplementary Figure 26: A comparison of the model predictions (blue line) and field data (orange points and line) for Athens, Greece in the years 2009 – 2010. Source data are provided as a Source Data file.

S.1.3. America

S.1.3.1. Lake Charles, Louisiana

Willis and Nasci (1994) measured the abundance and average wing length of adult mosquitoes in Lake Charles, Louisiana in the year 1988 [20]. Sampling of both host-seeking adults and of adults emerging from pupae collected from tyres was undertaken and reported separately. It was found that the average wing-length of emerging and host-seeking adults were significantly different, indicating that density dependent effects contribute to the population's trait dynamics. Supplementary Figure 27A shows a comparison of our predictions wing length to the observed average wing lengths and the average wing length of emerging adults. We see that our predictions of both the average wing length of host seeking adults and the average wing length of emerging adults align well with the field data. We achieve a model fit to observed adult activity of $R^2 = 0.95$, $sf = 0.016$, $ts = 14$ as shown in Supplementary Figure 27B.

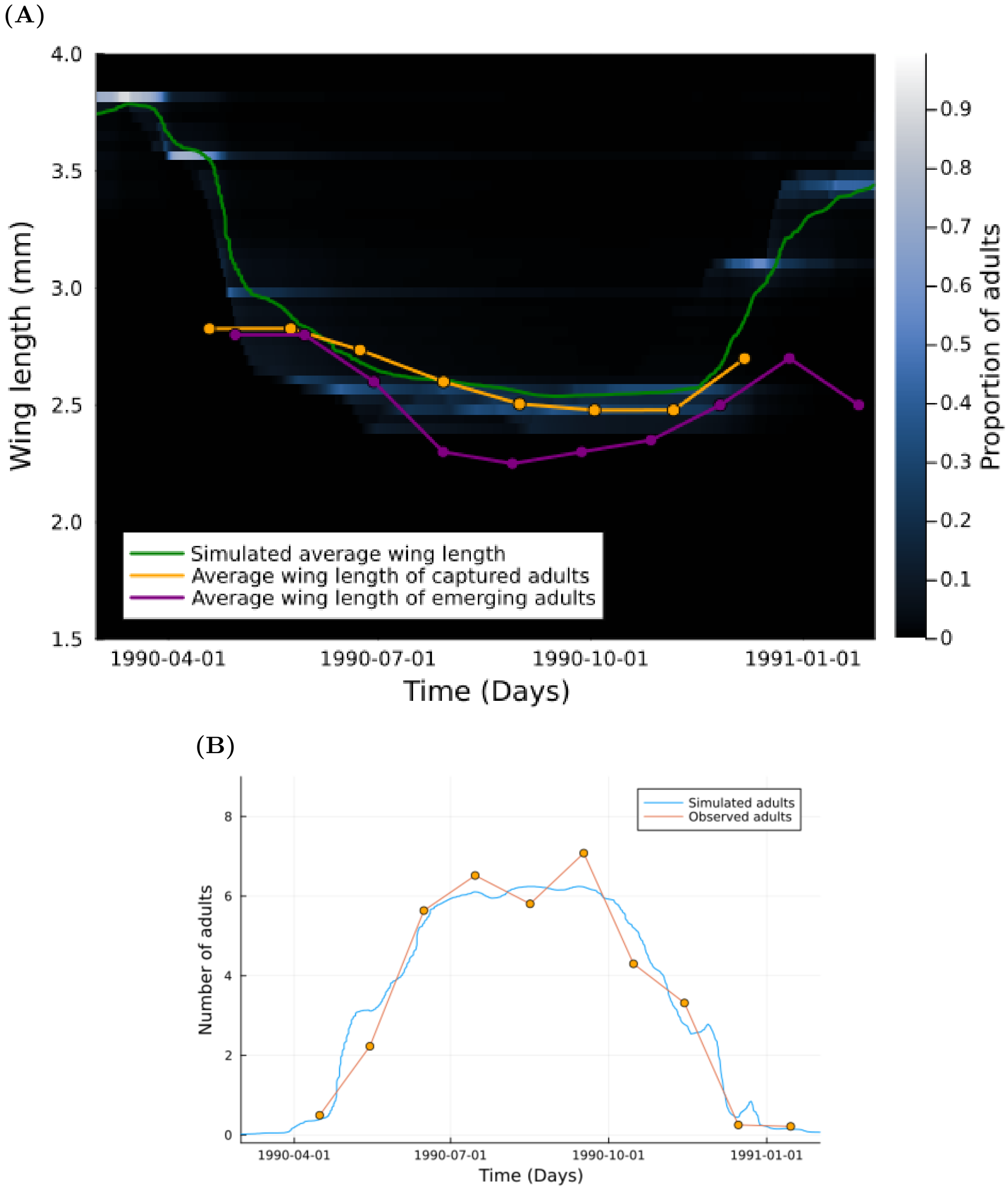

Supplementary Figure 27: Comparison of our predictions to observed field data for a population of *Aedes albopictus* in Lake Charles, Louisiana. Source data are provided as a Source Data file. **(A)** A comparison of our predictions of average wing length to that of adults in the field. The green line is our simulated average wing length with each blue line representing the proportion of adults within the simulated population that express a particular wing length. The orange line is the observed wing length of host seeking adults, and the purple line the observed average wing length of adults that emerged from pupae collected on the sampling days. **(B)** A comparison of the number of adults the model predicts are present at time t and the observed number of adults captured in the field.

S. 1.3.2. New Orleans, Louisiana

Comiskey et al. (1999) measured the wing length of *Aedes albopictus* in New Orleans in 1995. The method of trapping used is unknown and there was a prevalent infection throughout that altered both adult and larval mortality, reduced adult wing-length and had its own seasonal dynamics [21]. Supplementary Figure 28A shows that we do not completely capture the observed average wing length of the population. However, the resemblance to the population dynamics is better with the model fit to the larval dynamics being, $R^2 = 0.31$, $sf = 0.009$, $ts = -14$ (Supplementary Figure 28B), and the model fit to the adult dynamics being, $R^2 = 0.14$, $sf = 0.09$, $ts = 14$ (Supplementary Figure 28C).

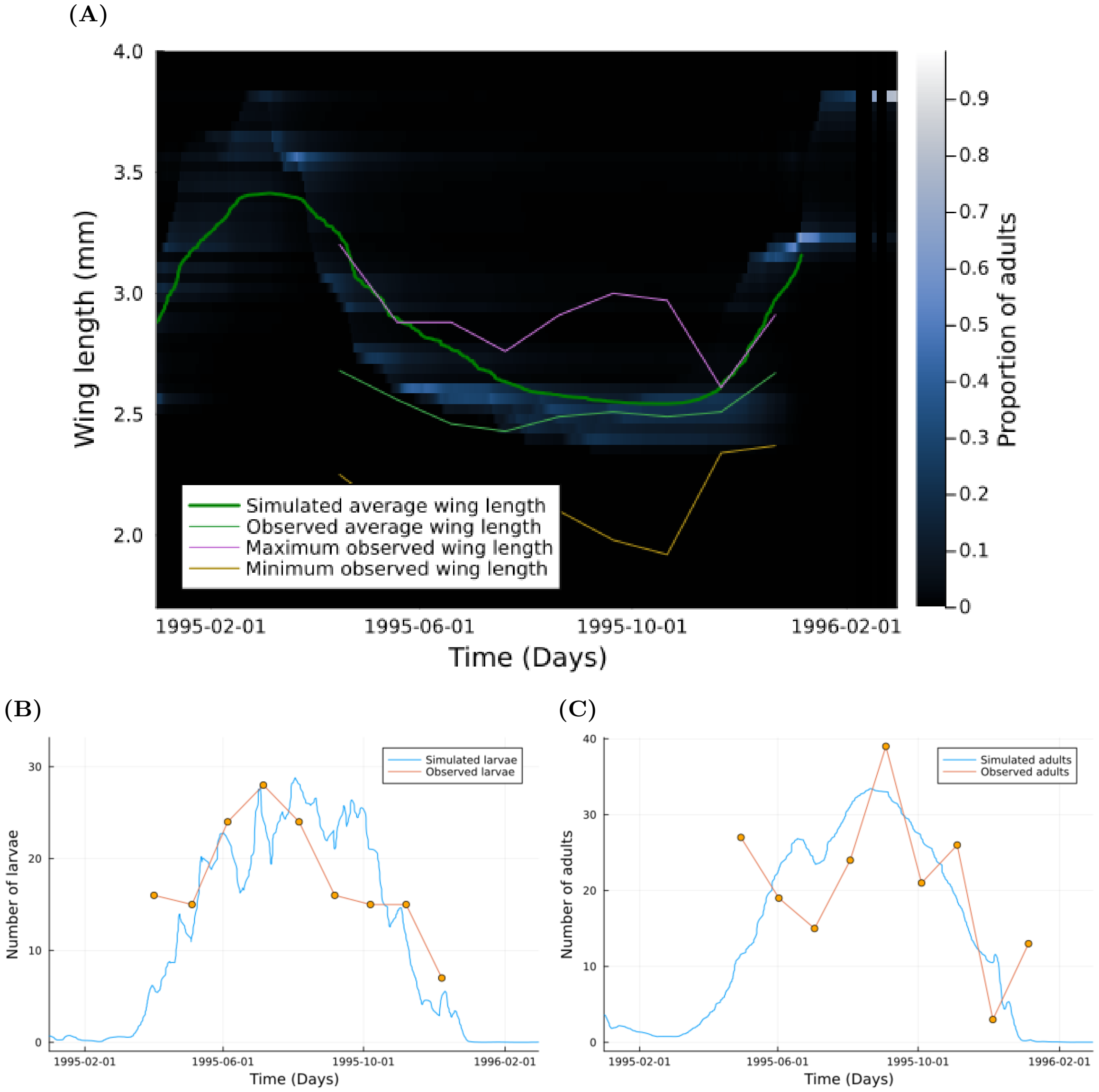

Supplementary Figure 28: Comparison of our predictions to observed field data for a population of *Aedes albopictus* in New Orleans. Source data are provided as a Source Data file. **(A)** A comparison of the observed and predicted trait dynamics. The green line is our simulated average wing length and each blue line represents the proportion of adults within the simulated population that express a particular wing length. The orange line is the observed wing length of host seeking adults, and the purple line the maximum wing length among adults sampled and the red line the minimum. **(B)** A comparison of observed and predicted larval dynamics. **(C)** A comparison of observed and predicted adult dynamics.

S. 1.3.3. Fort Worth, Texas

We compare our predictions to adult collections from CDC-gravid traps in Fort Worth, Texas with data sourced from Vectorbase (attributed to Nina Dacko, Tarrant County Public Health Department) [22]. We predict the field data with a fit of $R^2 = 0.47$, $sf = 0.002$,$ts = 14$, as shown in Supplementary Figure 29 .

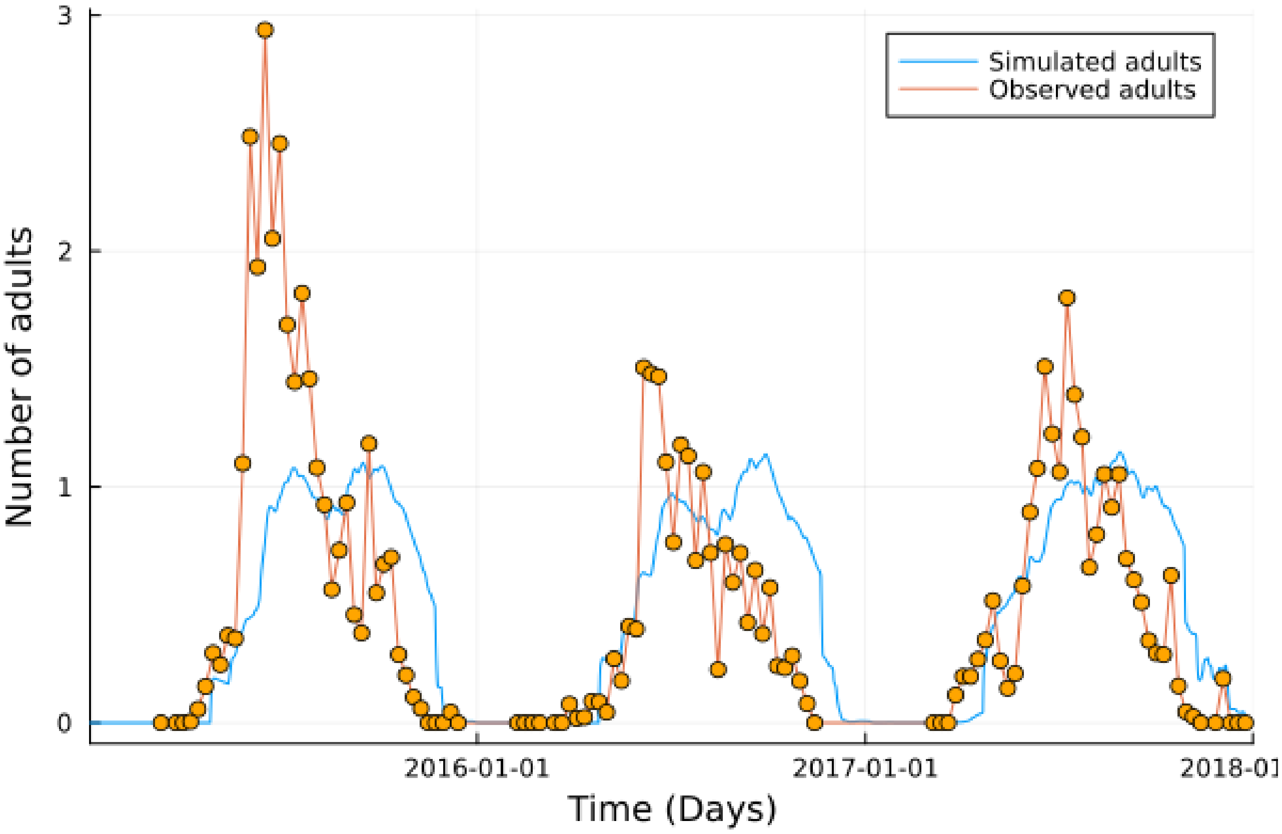

Supplementary Figure 29: Comparison of the model predictions to observed field data for a population of *Aedes albopictus* in Fort Worth, Texas in the years 2015 – 2017. Source data are provided as a Source Data file.

S. 1.3.4. Lubbock, Texas

We compare our predictions to adult collections from Texas Tech University between 2004 – 2008 as shown in Supplementary Figure 30 ($R^2 = 0.34$, $sf = 0.009$,$ts = 7$) [23].

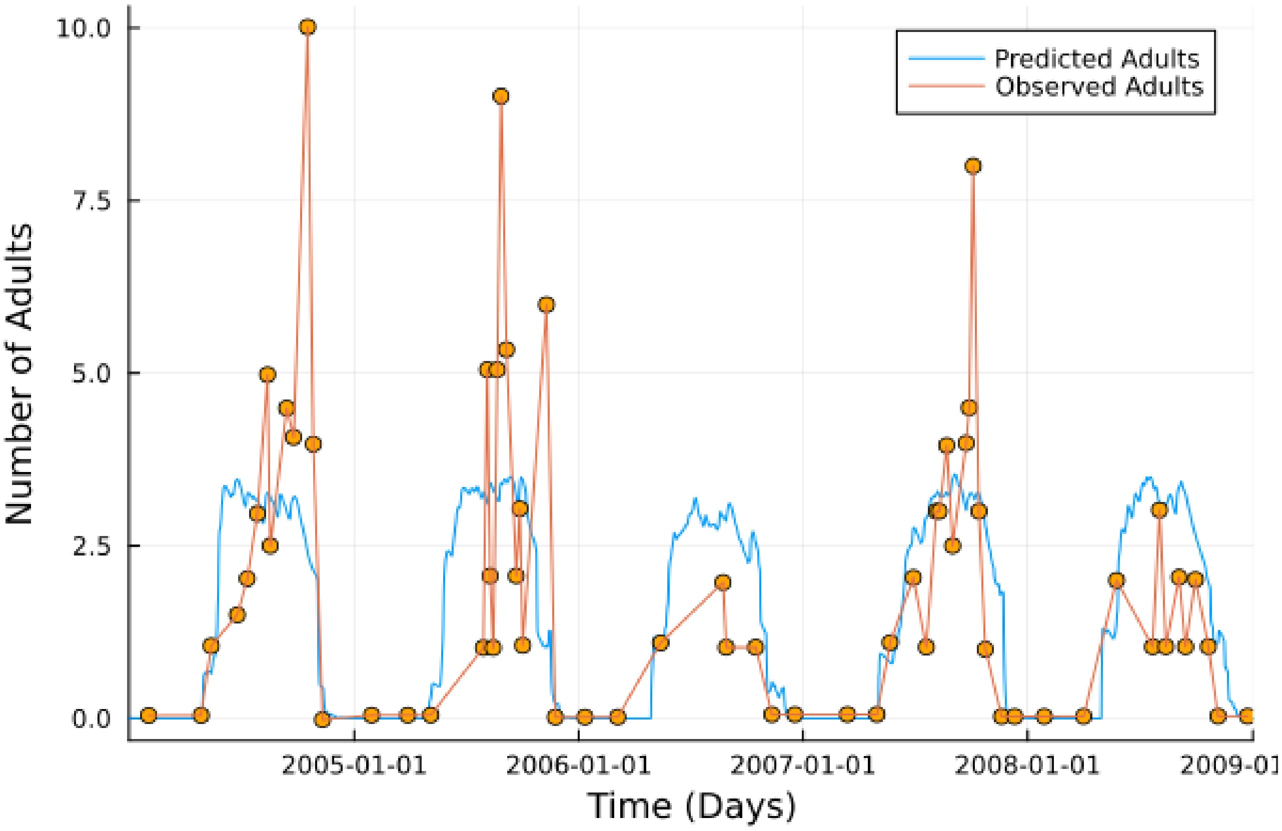

Supplementary Figure 30: Comparison of the model predictions to observed field data for a population of *Aedes albopictus* in Lubbock, Texas in the years 2004 – 2008. Source data are provided as a Source Data file.

S. 1.3.5. Stratford, Connecticut

Armstrong et al. (2017) monitored the number of adults and larvae in Stratford, Connecticut in the years 2013 – 2017 [24]. The fit for the adult data is $R^2 = 0.18$, $ts = 0$, $sf = 0.016$ (Supplementary Figure 31A), and for the larval data the fit is $R^2 = 0.25$, $ts = -1$, $sf = 0.008$ (Supplementary Figure 31B). The poor fit is potentially due to a failure to capture populations overwintering dynamics, as this population regularly experiences cold winters. Cold tolerance in *Ae. albopictus* is known to be genotypically plastic and so the disparity between model predictions and field observations would likely be improved by a more specific model. The poor fit could also be attributed to the lack of life-history data at lower temperatures meaning that cold weather survival is generally overestimated. Despite this, in the years 2013 and 2016 when the population is clearly established we adequately estimate the observed dynamics even if failing to capture the changes in relative abundance. For just these years a fit of $R^2 = 0.39$, $ts = 2$, $sf = 0.028$ is achieved to the adult data and a fit of $R^2 = 0.62$, $ts = -1$, $sf = 0.014$ to the larval data.

(A) **(B)**

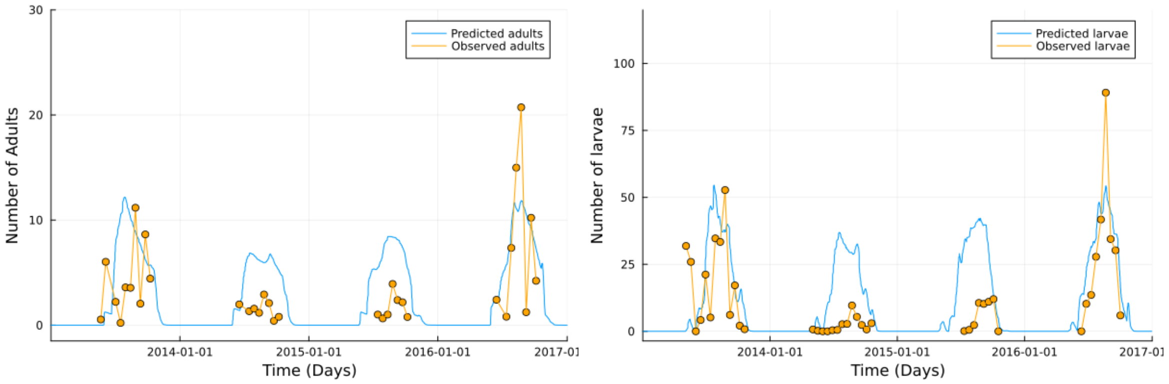

Supplementary Figure 31: A comparison of the model predictions (blue line) and field data (orange points and line) for Stratford, Connecticut in the years 2013 – 2017, for **(A)** number of adults **(B)** number of larvae. Source data are provided as a Source Data file.

S. 1.3.6. Monmouth, New Jersey

Fonesca et al. (2012) monitored adult numbers and oviposition activity in Mercer and Monmouth counties in New Jersey over the course of a year in intervention and non-intervention areas [25]. The dynamics of a population in Monmouth county are simulated and compared to the non-intervention data. We achieve a fit to the observed oviposition activity of, $R^2 = 0.59$, $sf = 0.0016$,$ts = -7$ (Supplementary Figure 32A) and to the adult activity of, $R^2 = 0.50$, $sf = 0.031$,$ts = 4$ (Supplementary Figure 32B).

(A)

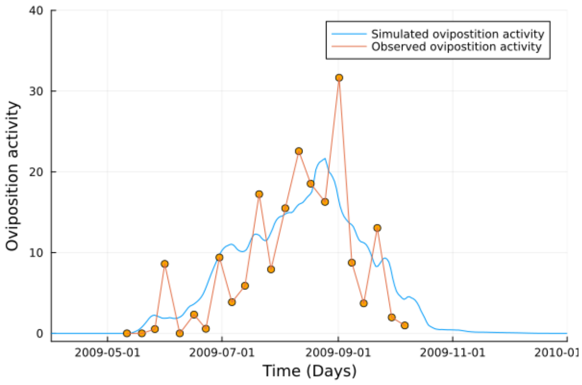

(B)

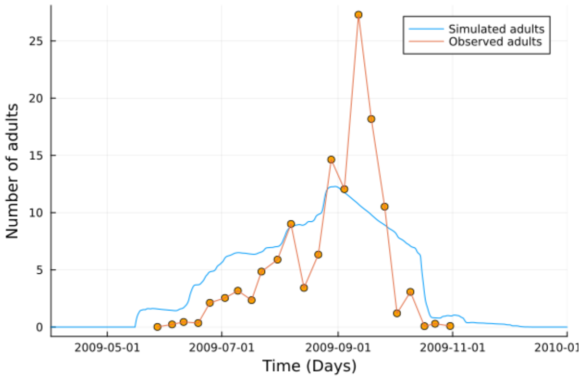

Supplementary Figure 32: A comparison of the model predictions (blue line) and field data (orange points and line) for Monmouth, New Jersey in the years 2009, of **(A)** oviposition activity **(B)** number of adults. Source data are provided as a Source Data file.

S. 1.3.7. Charlotte, North Carolina

Three different studies monitored the dynamics of *Ae. albopictus* in Charlotte, North Carolina. Mundis et al. (2021) measured the average wing-length of adults captured by gravid *Aedes* traps and our predictions align well with the observed trait dynamics (Supplementary Figure 33A) [26]. Field data for the oviposition activity of *Ae. albopictus* in the year 2016 as used in the study Reed et al. (2018) was obtained from VectorBase [22, 27]. Supplementary Figure 33B shows that oviposition activity data achieves a fit of, $R^2 = 0.64$, $ts = 14$, $sf = 0.0018$. Whiteman et al. (2018) monitored the number of gravid adults in Charlotte in 2017, to which we achieve a fit of $R^2 = 0.10$, $ts = 10$, $sf = 0.01$ (Supplementary Figure 33C) [28].

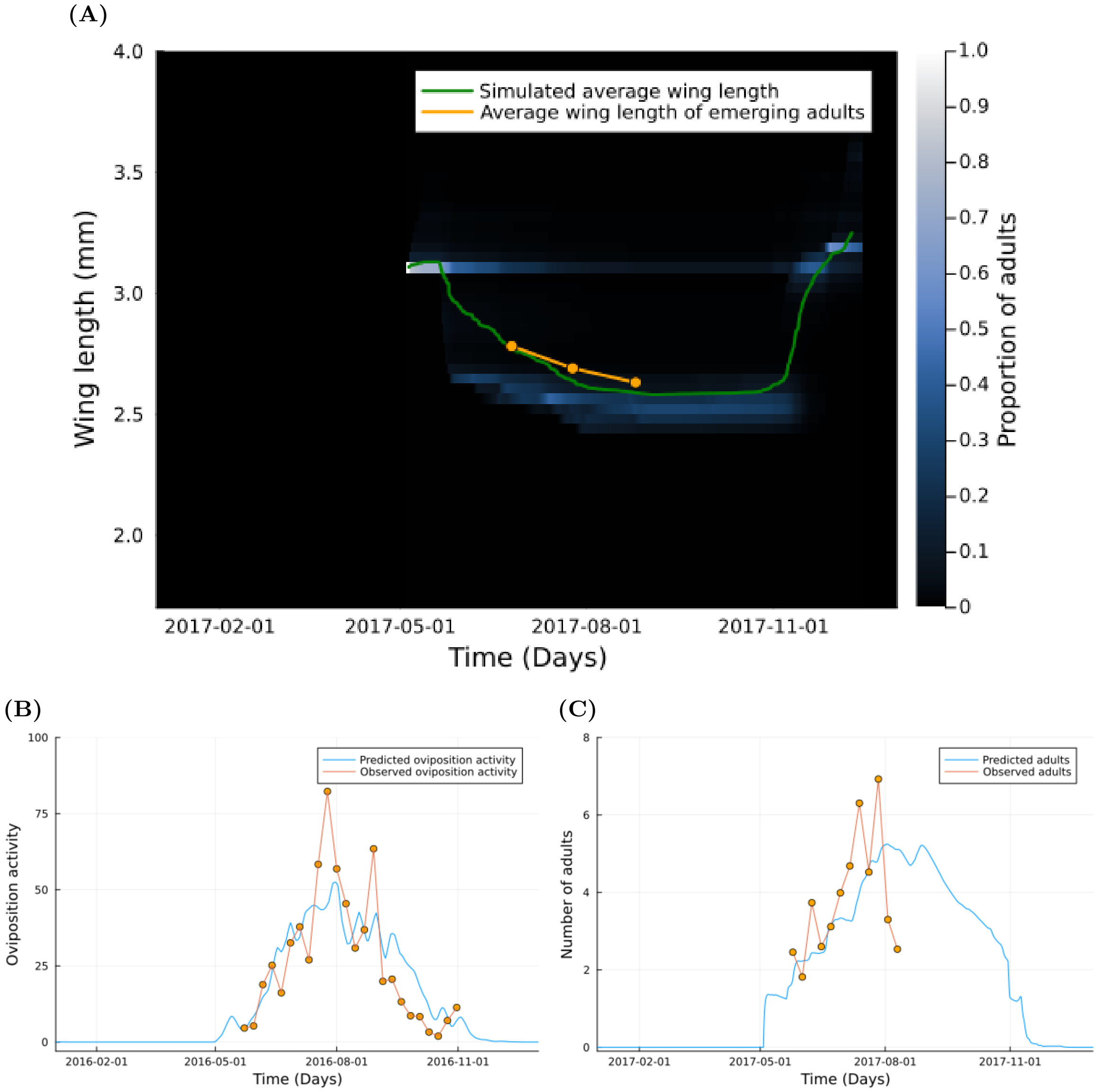

Supplementary Figure 33: A comparison of the model predictions (blue line) and field data (orange points and line) for Charlotte, North Carolina in the year 2016, for **(A)** Trait values, **(B)** Oviposition activity, **(C)** Adult numbers. Source data are provided as a Source Data file.

S. 1.3.8. Raleigh, North Carolina

Reed et al. (2019) monitored the oviposition activity in Raleigh, North Carolina. We predict the observed dynamics with a fit of, $R^2 = 0.78$, $sf = 0.003$,$ts = 6$, as shown in Supplementary Figure 34.

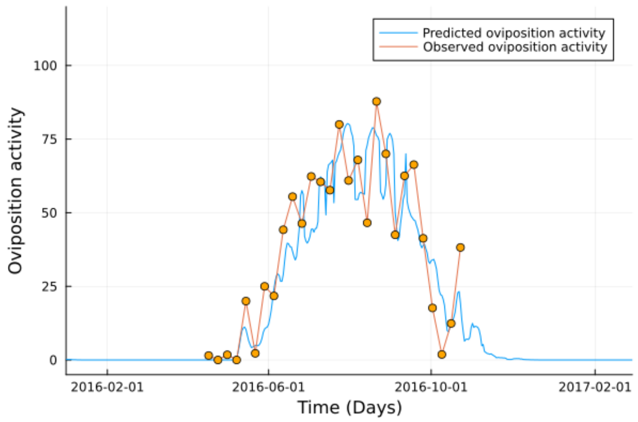

Supplementary Figure 34: A comparison of the model predictions (blue line) and field data (orange points and line) for Raleigh, North Carolina in the year 2016. Source data are provided as a Source Data file.

S. 1.3.9. Asheville, North Carolina

Reed et al. (2019) monitored the oviposition activity in Asheville, North Carolina. We predict the observed dynamics with a fit of, $R^2 = 0.63$, $sf = 0.0014$,$ts = 1$, as shown in Supplementary Figure 35.

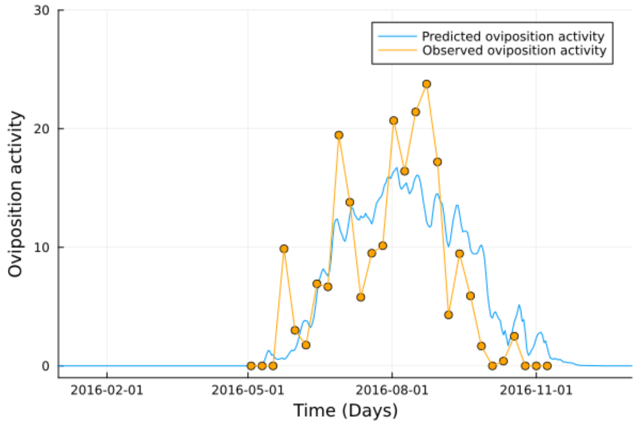

Supplementary Figure 35: A comparison of the predictions of the model (blue line) and field data (orange points and line) for Asheville, North Carolina in the year 2016. Source data are provided as a Source Data file.

S. 1.3.10.   Greenville, North Carolina

Reed et al. (2019) monitored the oviposition activity in Greenville, North Carolina.We predict the observed dynamics with a fit of, $R^2 = 0.51$, $sf = 0.002$,$ts = 6$, as shown in Supplementary Figure 36.

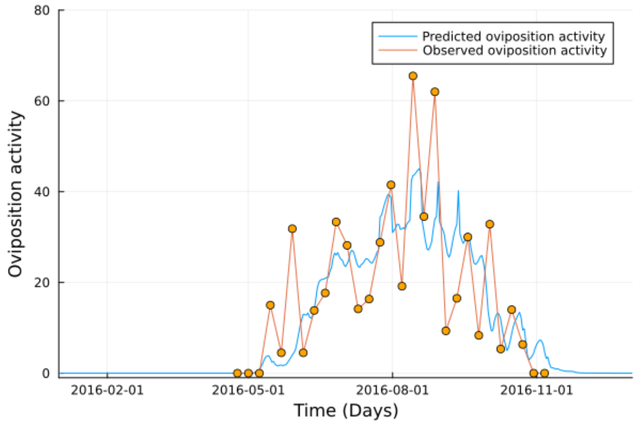

Supplementary Figure 36: A comparison of the predictions of the model (blue line) and field data (orange points and line) for Greenville, North Carolina in the year 2016. Source data are provided as a Source Data file.

S. 1.3.11. Indianapolis, Indiana

We obtained adult data from VectorBase for Indianapolis, Indiana for the years 2018 − 2020 (attributed to Sinsko Matthew from Marion County Health Department) [22]. We achieve a fit of $R^2 = 0.72$, $sf = 0.0009$, $ts = -11$ as seen in Supplementary Figure 37.

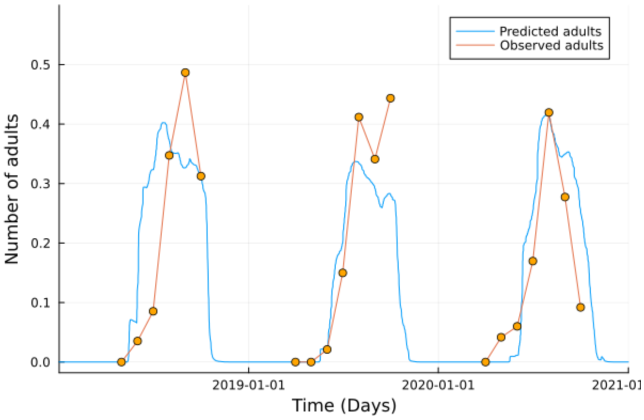

Supplementary Figure 37: A comparison of the predictions of the model (blue line) and field data (orange points and line) for Indianapolis, Indiana for the years 2019 − 2020. Source data are provided as a Source Data file.

S. 1.3.12. Washington, D.C.

Adult trapping data was obtained from Washington D. C. from VectorBase (attributed to Stephen Panossian from the Maryland Department of Agriculture Mosquito Control Section) [22]. We achieve a fit to this data of $R^2 = 0.23$, $sf = 0.06$, $ts = 12$ (Supplementary Figure 38).

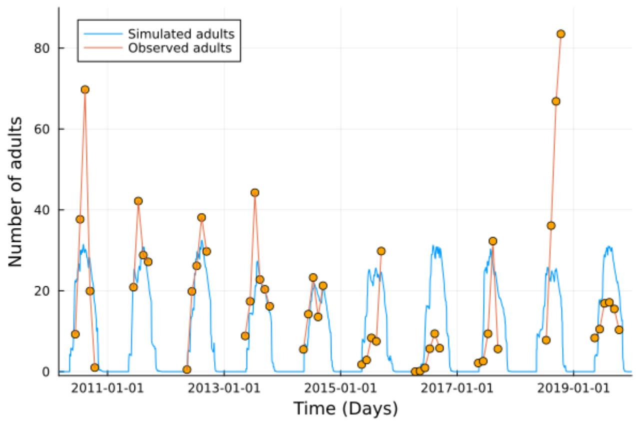

Supplementary Figure 38: A comparison of the model predictions (blue line) and field data (orange points and line) for Washington, D.C. for the years 2010 – 2014. Source data are provided as a Source Data file.

S. 1.3.13. Colombus, Ohio

We obtained adult trapping data from VectorBase for Colombus, Ohio in 2018 (attributed to Sarah Fink from Franklin County Public Health) [22]. We achieve a fit of $R^2 = 0.3$, $sf = 0.017$,$ts = -8$ as seen in Supplementary Figure 39.

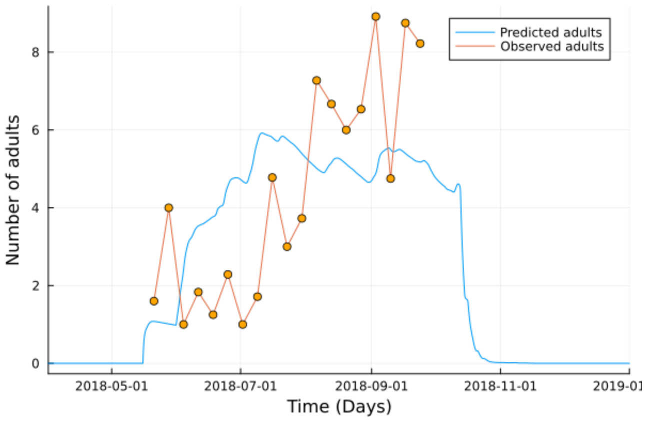

Supplementary Figure 39: A comparison of the model predictions (blue line) and field data (orange points and line) for Colombus, Ohio for the years 2018. Source data are provided as a Source Data file.

S. 1.3.14. Suffolk, Virginia

We obtained adult data from VectorBase for Suffolk, Virginia for the years 2009 – 2018 (attributed to Karen Akaratovic, City of Suffolk Mosquito Control) [22]. We achieve a fit of $R^2 = 0.64$, $sf = 0.14$, $ts = 8$ as seen in Supplementary Figure 40.

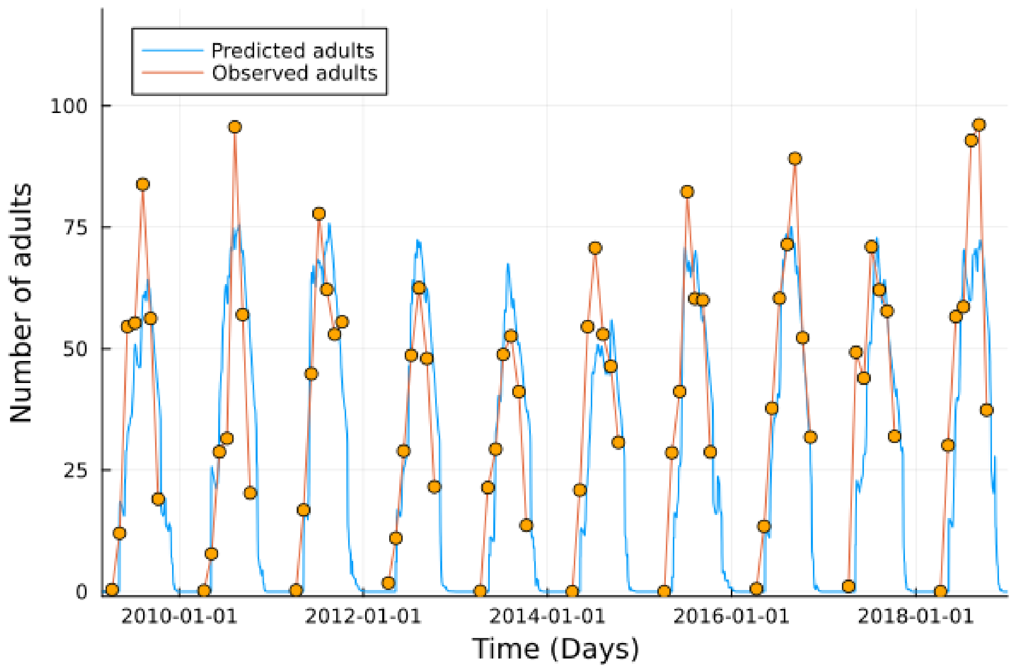

Supplementary Figure 40: A comparison of the model predictions (blue line) and field data (orange points and line) for Suffolk, Virginia in the years 2009 – 2018. Source data are provided as a Source Data file.

S. 1.3.15. Santa Rosa Beach, Florida

We obtained adult data gathered by Brabant et al. (2018) from VectorBase for Santa Rosa Beach, Florida for the years 2014 − 2017 [22, 29]. We achieve a fit of $R^2 = 0.3$, $sf = 0.0019$, $ts = 7$ as seen in Supplementary Figure 41.

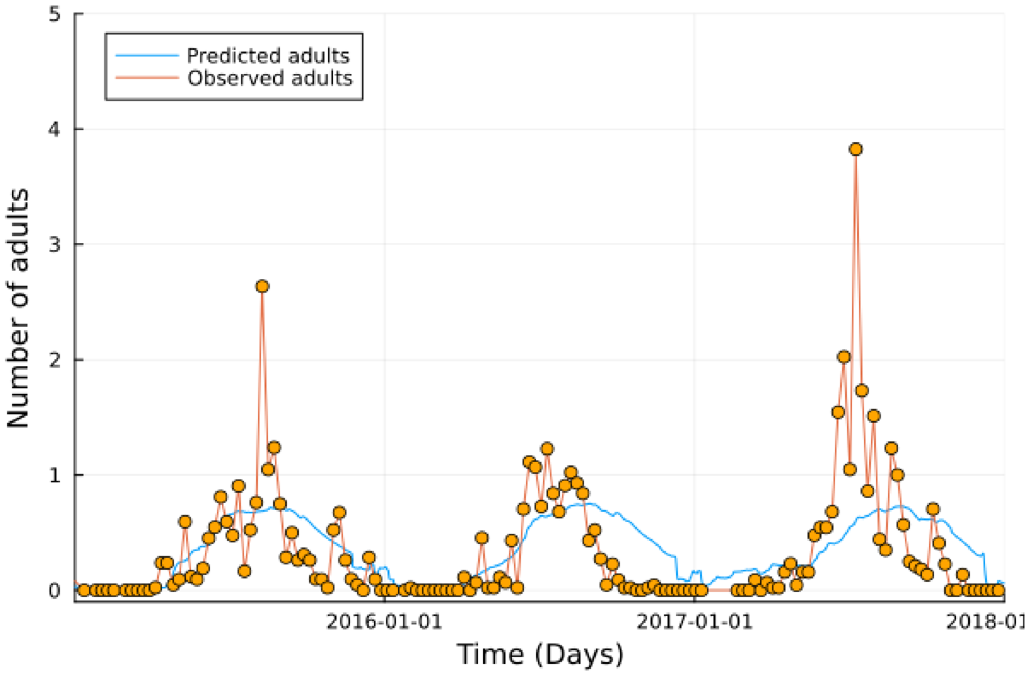

Supplementary Figure 41: A comparison of the model predictions (blue line) and field data (orange points and line) for Santa Rosa Beach, Florida in the years 2014 − 2017. Source data are provided as a Source Data file.

S. 1.4. Asia

S. 1.4.1. Naha, Japan

Toma et al. 1982 monitored the population dynamics of *Ae. albopictus* in Naha, Japan in the years 1978 − 1979 [30] . This study is notable as the only validation set to report data from all life-stages and reports the number of eggs, larvae, pupae, and adults in the years in which the study was conducted. The model achieves a fit to the observed oviposition activity of $R^2 = 0.3$, $ts = 14$, $sf = 0.02$, to the observed larval dynamics is $R^2 = 0.4$, $ts = 11$, $sf = 0.04$, and to the adult data is $R^2 = 0.41$, $ts = 14$, $sf = 0.04$.

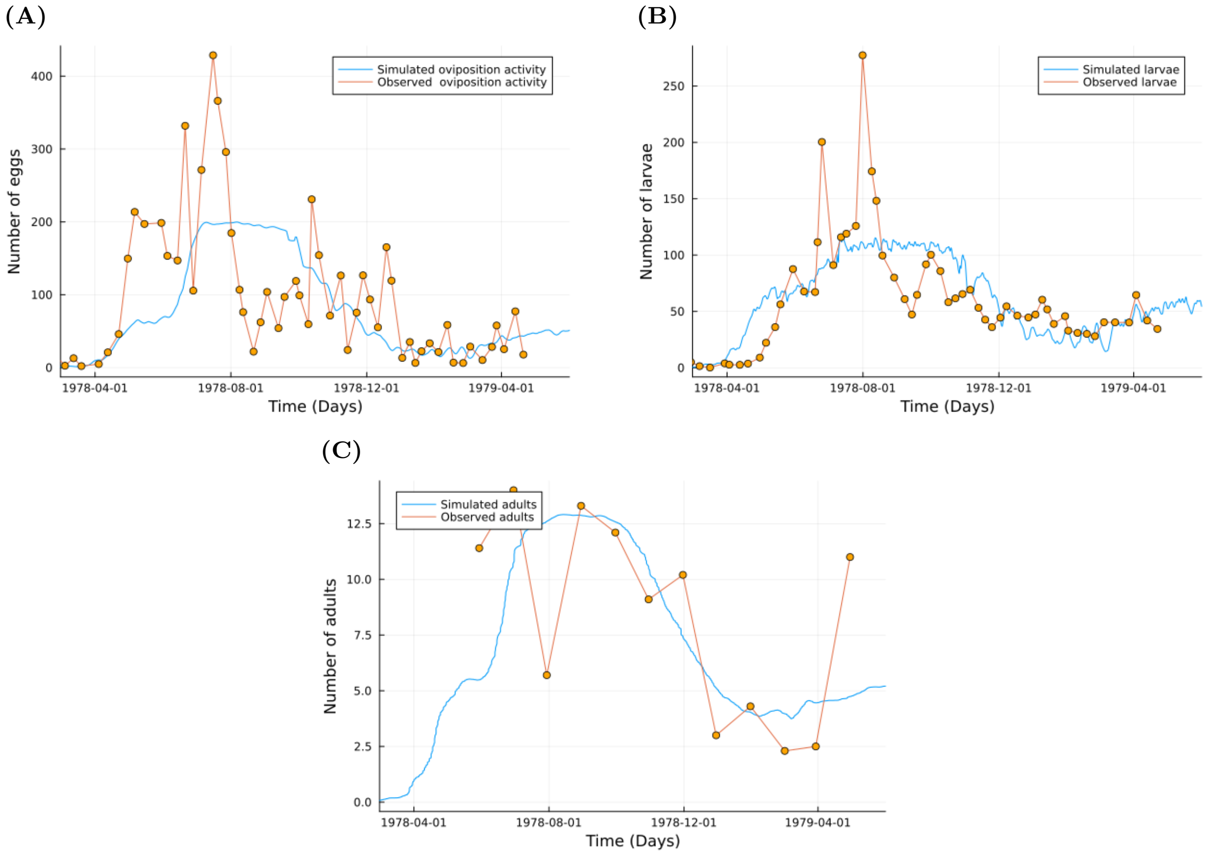

Supplementary Figure 42: A comparison of the model predictions (blue line) and field data (orange points and line) for Naha, Japan in the year 1978 for **(A)** Oviposition activity. **(B)** Number of larvae. **(C)** Number of adults. Source data are provided as a Source Data file.

S.1.4.2. Nagasaki, Japan

Suzuki et al. (1993) monitored adults in Nagasaki, Japan in (1990) [31]. In Supplementary Figure 43A we achieve a good fit to the adult data, with, $R^2 = 0.78$, $ts = 4$, $sf = 0.14$. The same authors measure the average wing lengths of adults collected during this period and the model adequately predicts the observed seasonal variation as can be seen in Supplementary Figure 43B.

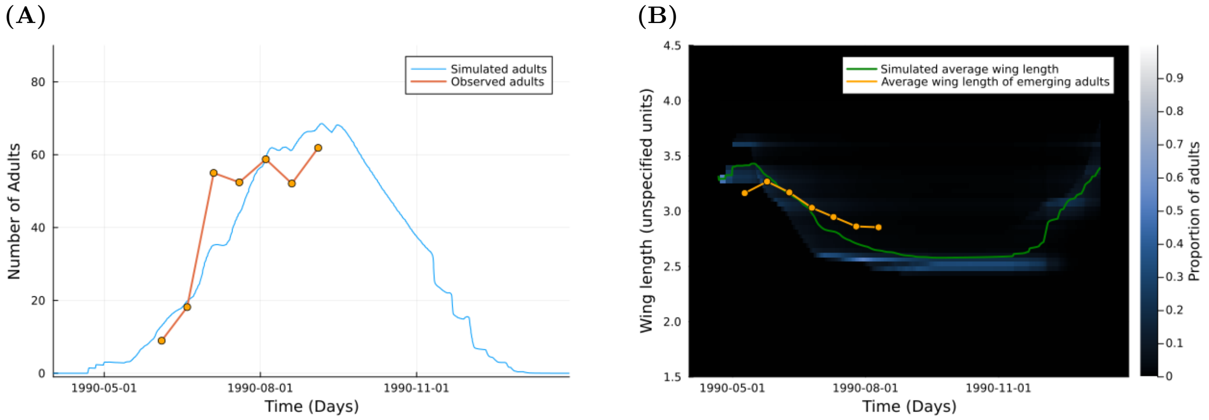

Supplementary Figure 43: A comparison of the model predictions (blue line) and field data (orange points and line) for Nagasaki, Japan in 1990 for **(A)** Adults numbers. **(B)** Average wing length. Source data are provided as a Source Data file.

S. 1.4.3. Tokyo, Japan

Kori et al. (2020) monitored the number of adults in parks around Tokyo in years between 2010 and 2018, and adult and larval numbers in Yoyogi park in the years 2015 − 2017 [32]. After a dengue outbreak in 2014 larval habitats were destroyed in the Tokyo metropolitan area and adulticides and larvicides are now regularly used in these areas and this may explain the general poor fit the model achieves to the majority of this data. To the adult collections from all major parks we achieve a fit of $R^2 = 0.42$, $ts = 3$, $sf = 0.0105$ in Supplementary Figure 44A. To the adult collections from Yoygi park after the intervention we achieve a fit of $R^2 = 0.43$, $ts = 4$, $sf = 0.22$ in Supplementary Figure 44B. To the larval collections from Yoyogi park we achieve a fit of $R^2 = 0.01$, $ts = 14$, $sf = 0.028$ in Supplementary Figure 44C.

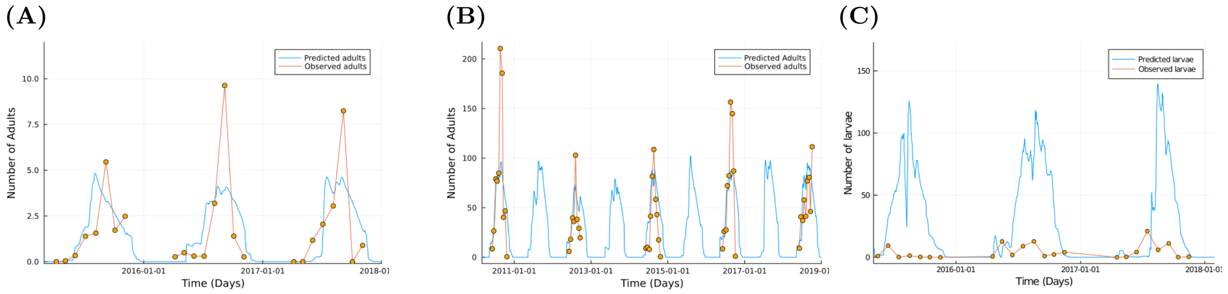

Supplementary Figure 44: A comparison of the model predictions (blue line) and field data (orange points and line) for Tokyo, Japan in years between 2010 and 2018 for **(A)** Adult numbers observed in major parks around Tokyo. **(B)** Adult numbers observed in Yoyogi park in the years 2015 − 2017. **(C)** Larval numbers observed in Yoyogi park in the years 2015 − 2017. Source data are provided as a Source Data file.

S.1.4.4. Guangzhou, China

In Guangzhou, Xia et al. (2018) measured oviposition activity in 2017 and Xu et al. (2017) monitored adult numbers in the years 2006 – 2015 [33, 34]. In Supplementary Figure 45B it should be noted that in 2015 control activities were increased following a large dengue outbreak in 2014 explaining the low observed adults that year and so this year is excluded from our model fitting. We achieve a fit of, $R^2 = 0.41$, $ts = 8$, $sf = 0.019$, to the oviposition activity data (see Supplementary Figure 45A) and a fit of, $R^2 = 0.6$, $ts = 4$, $sf = 0.0038$ to the adult data (see Supplementary Figure 45B).

(A)

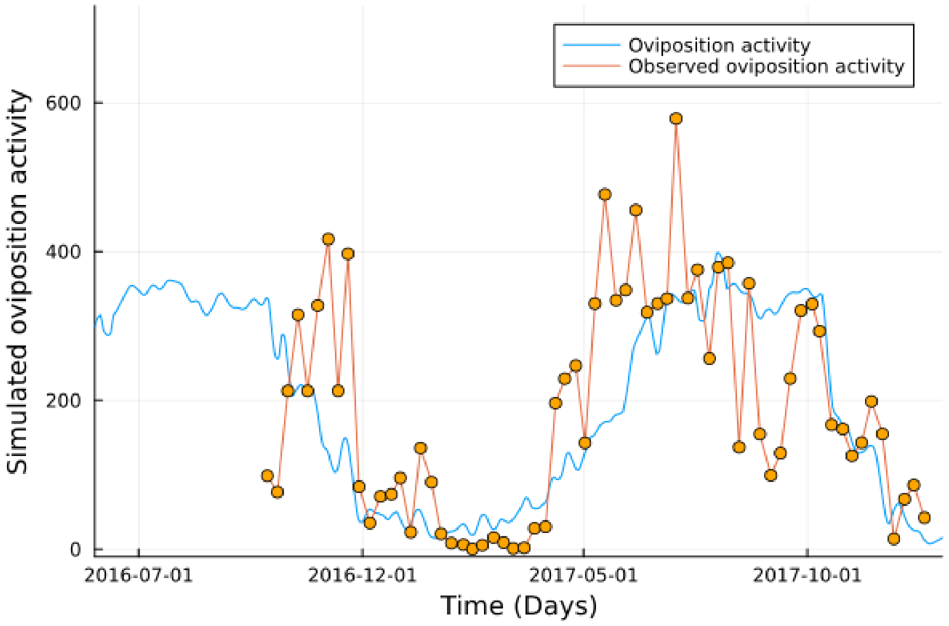

(B)

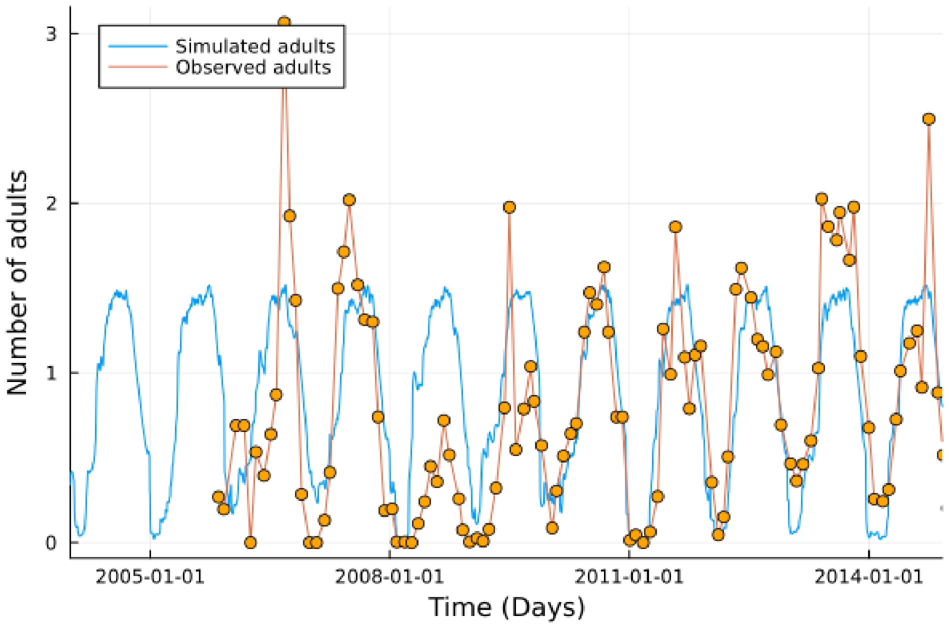

Supplementary Figure 45: A comparison of the model predictions (blue line) and field data (orange points and line) for Guangzhou, China over the years 2006 – 2018. Source data are provided as a Source Data file. **(A)** Oviposition activity for 2017 with data taken from Xia et al. (2019). **(B)** Adult abundance for the years 2006 – 2015 with data taken from Xu et al. (2017).

S. 1.4.5. Suwon, South Korea

Hwang et al. (2020) [35] monitored adult numbers in Suwon, South Korea in the year 2016. In Supplementary Figure 46 we show the model predictions achieve a fit of $R^2 = 0.93$, $ts = 3$, $sf = 0.04$.

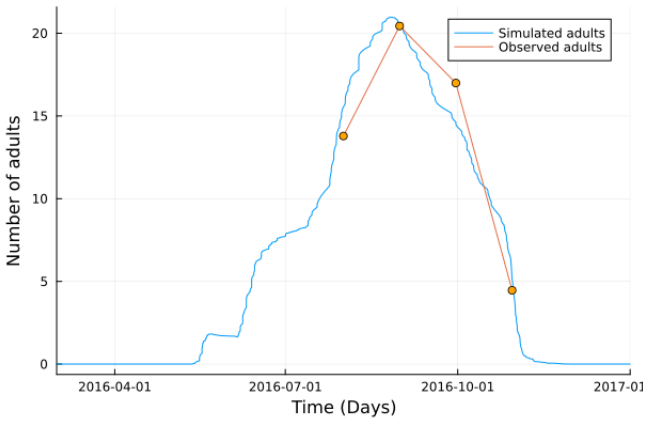

Supplementary Figure 46: A comparison of the model predictions (blue line) and field data (orange points and line) for Suwon, South Korea in the year 2016. Source data are provided as a Source Data file.

S. 1.5. Africa

S. 1.5.1. La Reunion

Gougna et al. 2010 [36] monitored oviposition activity and observed around 200 eggs per ovitrap in each sample between October to April, which we find is in line with our predictions of oviposition activity. Harambourne et al. (2020) [37] performed larval surveys in five locations across the north of La Réunion in the years 2012 and 2013. In Supplementary Figure 47 we see that the model adequately predicts observed differences in larval dynamics between locations and years. In Saint-Paul we predict the observed population dynamics with, $R^2 = 0.65$, $ts = -3$, $sf = 0.024$, see Supplementary Figure 47A. In La Possession our prediction achieves a fit of, $R^2 = 0.53$, $ts = -2$, $sf = 0.023$, see Supplementary Figure 47B. In Saint-Bennoit we predict, $R^2 = 0.14$, $ts = -3$, $sf = 0.11$, as shown in Supplementary Figure 47C. In Saint-Marie we predict, $R^2 = 0.29$, $ts = 5$, $sf = 0.026$ as shown in Supplementary Figure 47B.

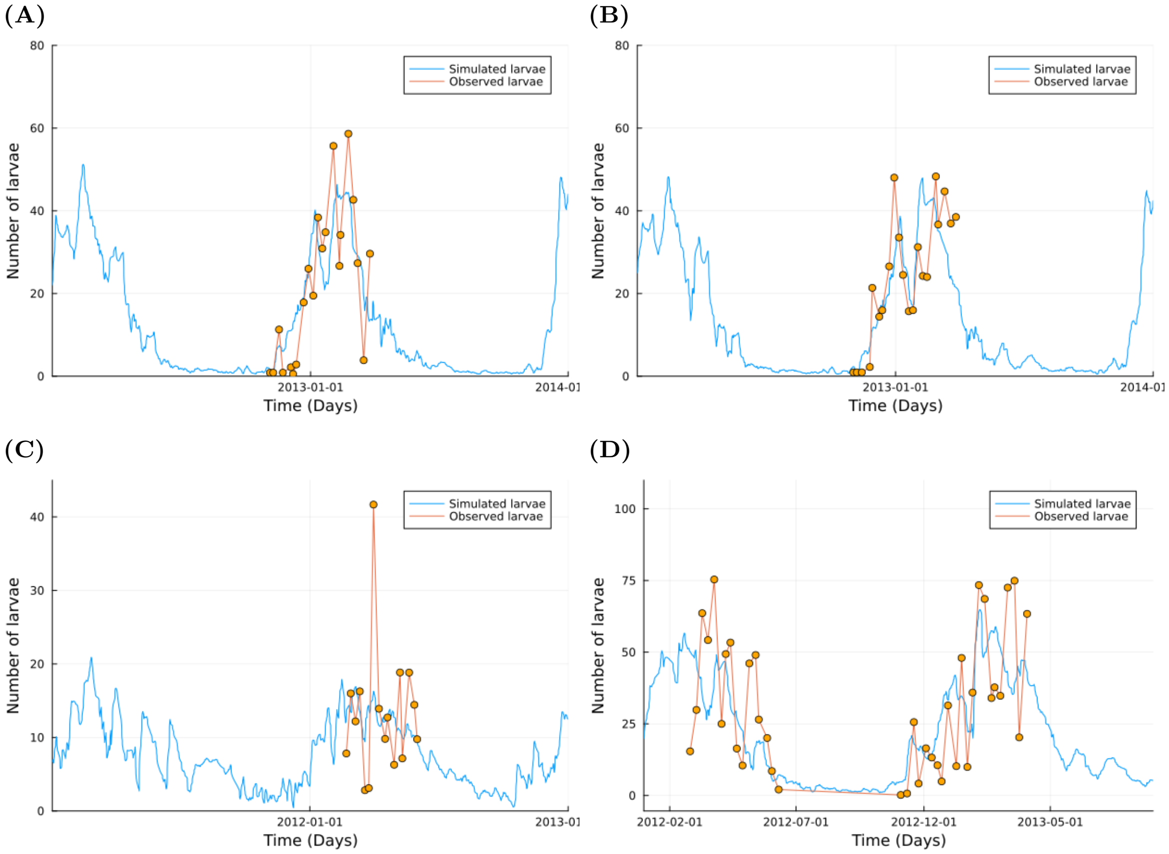

Supplementary Figure 47: A comparison of the model predictions (blue line) and field data (orange points and line) for larvae in sites around La Réunion. **(A)** Saint-Paul **(B)** La Possession **(C)** Saint-Bennoit **(D)** Saint-Marie. Source data are provided as a Source Data file.

In Saint-Marie we additionally have oviposition activity data for the years $2013 - 2014$ to which we achieve a fit of $R^2 = 0.06$, $ts = 7$, $sf = 0.015$ as shown in Supplementary Figure 48.

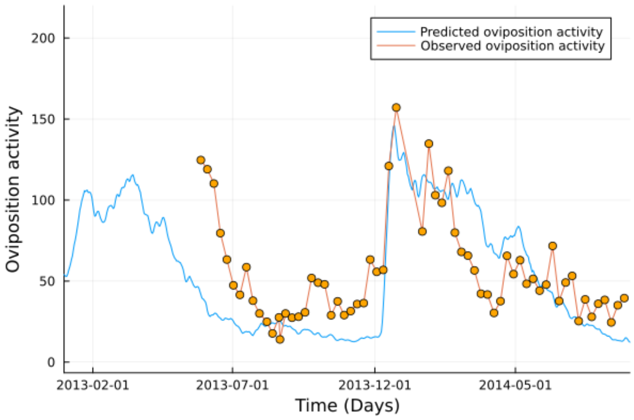

Supplementary Figure 48: A comparison of the model predictions (blue line) and field data (orange points and line) for oviposition activity in Saint-Marie in the years 2013 − 2014. Source data are provided as a Source Data file.

S. 2. Validations of the SEIR model

To validate the SEIR model we compare the daily or weekly number of dengue cases observed over the course of real-world dengue outbreaks. Unlike our validations for the model of the population dynamics of *Ae. albopictus* we do not apply any scaling factors, although we do apply a uniform time-shift of 14 days to roughly approximate the delay between when a dengue case is contracted and when it is detected. In addition to the usual environmental parameters we require an estimate of human population density, we need to define a scenario for the introduction of dengue into the human population, and choose an area over which to simulate the model. To determine human population density we use the Gridded Population of the World, Version 4 (GPWv4) [38]. To determine the introduction of infected humans into the population we look to contact tracing and imported case detection undertaken during the course of the outbreak. We determine the area an outbreak region by consulting the spatial distribution of dengue cases.

For many of the outbreaks we consider the number and timing of case introductions is not precisely known. Even for those outbreaks where considerable effort was expended on tracking introduction events the sensitivity of the model to the precise timing of these events and the stochastic nature of the initial stages of an outbreak means that there are many reasonable introduction scenarios we might choose, even when case reporting is reliable.

In each of the outbreak locations different forms of mosquito control activity were employed which we do not account for in any way. Control activities commonly include larval source reduction, the application of adulticides and larvicides, in combination with public health awareness campaigns and so we do not expect the effect of these control activities to be uniform across outbreak locations. Our aim with these validations is therefore only to demonstrate that under a reasonable set of assumptions we can predict the dynamics and magnitude of each dengue outbreak.

S. 2.1. Cagnes-sur-Mer

In this region of France there are sporadic instances of autochthonous transmission of dengue that occur between the months of August and October [39]. These outbreaks are typical of those currently experienced throughout Europe where despite the regular introduction of dengue there is little evidence of sustained cycles of disease transmission. We simulate the SEIR model in this region for the years 2018, 2019, and 2020 under an introduction scenario such that a single infected individual is introduced on the first day of the first month that a dengue transmission event was reported for human population of 2088 people per km^2. In Supplementary Figure 49 we see that in each year we predict a very limited amount of dengue spread, within the same order of magnitude as is observed in the field.

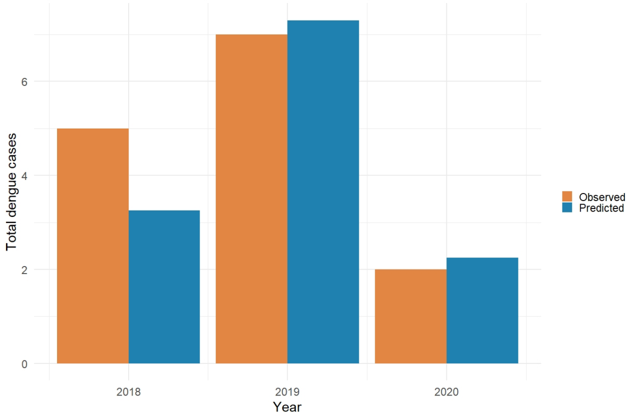

Supplementary Figure 49: A comparison of the total number of dengue cases observed in the Alpes-Maritimes department of France with predictions in the years $2018 - 2020$. Source data are provided as a Source Data file.

S. 2.2. Guangzhou

There are regular dengue outbreaks in the Guangdong region of China, for which *Ae. albopictus* is the primary vector, and here we use the SEIR model to predict dengue dynamics in the years for which detailed dengue onset data is available. We specifically consider 2013, in which there was a relatively small outbreak, and 2014 in which there was a much larger outbreak [34]. High numbers of cases were observed over an area of roughly 80 km^2 in a region in which there is an average population density of 32,113 people per km^2 [40]. Using the SEIR model we reproduce the infectious individual introduction scenarios described in Luo et al. (2017) introducing 30 infectious individuals over the region of interest through the year in 2014 with a limited initial introduction of a single individual in 2013. In Supplementary Figure 50 we see that under these conditions we produce convincing dengue dynamics over the years of interest, capturing the relative magnitude of the outbreaks in addition to the initial period of infection. In 2013 that we also capture the dynamics towards the end of the season, but not in 2014. This mismatch likely occurs due to the substantial intervention that occurred in response to the 2014 outbreak which likely prevented the continued transmission of disease. The total number of dengue cases observed in 2014 in Guangzhou city was 36,342 and a previous study, based on time series analysis, estimated that the intervention prevented an additional 23,302 cases of dengue [41]. We broadly agree with this figure and predict that the intervention prevented an additional 27,907 cases of dengue in 2014.

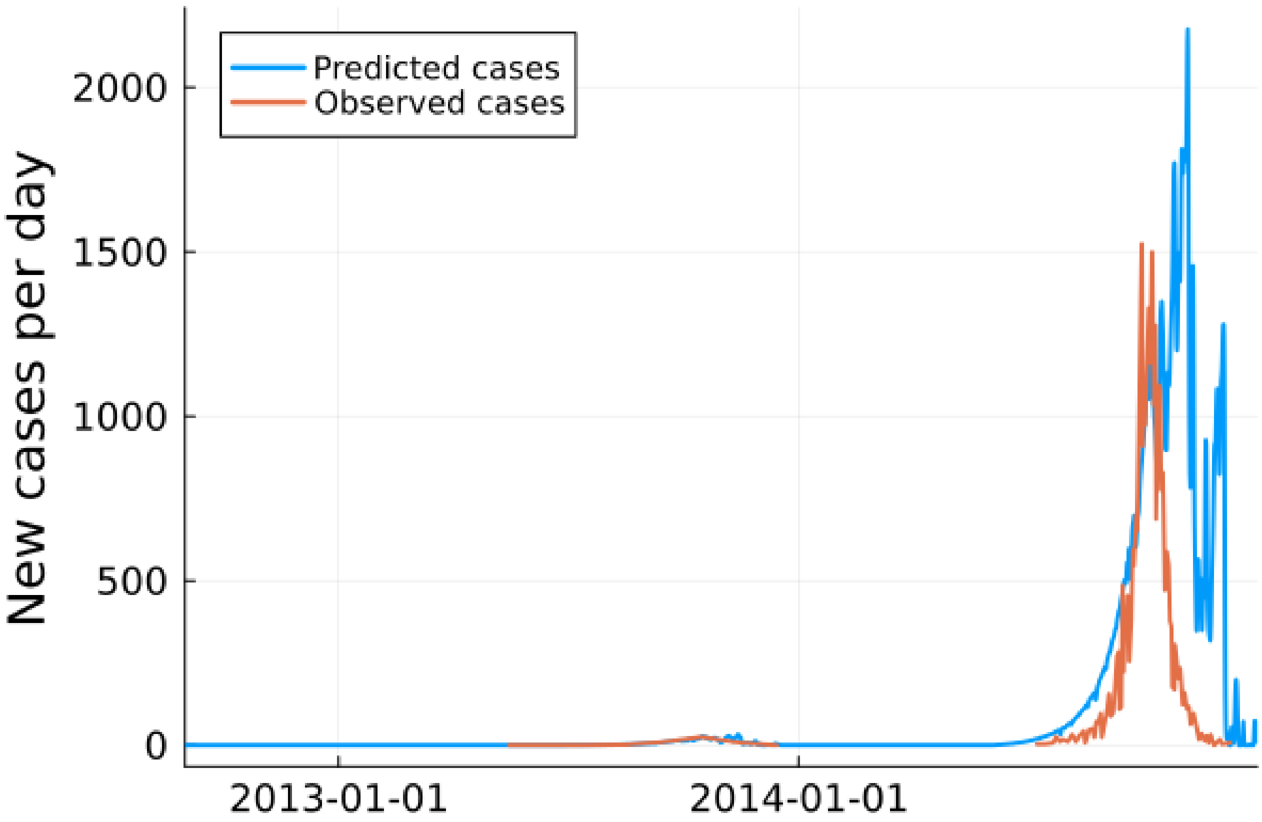

Supplementary Figure 50: A comparison of the model predictions (blue line) and disease incidence data (orange line) for Guangzhou, China for the years 2013 – 2014. Source data are provided as a Source Data file.

S. 2.3. Tokyo

In 2014 in Tokyo there was an outbreak of dengue fever centered around Yoyogi park. This outbreak coincided with an international festival that made determining the initial introduction event difficult, and so we choose an introduction scenario similar to that used in Guangzhou, with the first case introduced on the 1st day of the first month that transmission was reported into a human population of density 12254 people per km^2. In Supplementary Figure 51 we predict the dengue dynamics well, and it appears that the intervention had minimal effect on transmission dynamics.

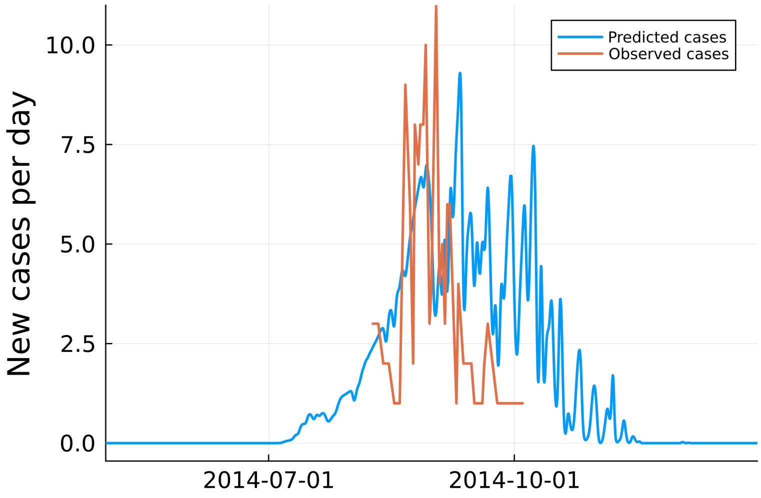

Supplementary Figure 51: A comparison of the predictions of the model (blue line) and disease incidence data (orange line) for Tokyo, Japan for the year 2014. Source data are provided as a Source Data file.

S. 2.4. Reunion

We use the SEIR model to predict the dengue dynamics of the $2017-2021$ dengue outbreak. This outbreak began in 2016 when DENV-II replaced the previously circulating DENV-I, however the first year in which DENV-II was the dominant serotype was 2017 when it circulated at low levels. After subsiding, transmission was renewed in 2018 resulting in a moderate outbreak that caused $6,770$ cases of dengue over the whole year. After once again subsiding, the outbreak continued in 2019 reaching a peak of cases and then being replaced by the end of the year by an outbreak of DENV-I that continued into 2021 [42].

For our prediction of this outbreak we define an introduction scenario where infected individuals begin entering the population in 2017, into a population of 4000 people per km^2. Previous to the $2017-2021$ outbreak the last large outbreak of dengue in on Reunion occurred in 1978 [43]. Further, in the years preceding this outbreak the dominant circulating serotype was DENV-I. We therefore assume that the population of Reunion is initially completely susceptible to the introduced dengue serotype. The introduction is at a low level, in line with the number of introduced dengue cases observed [44]. We simulate the dynamics until the end of the outbreak in 2021 and compare the number of cases we predict to those that were observed. In Supplementary Figure 52, we predict the relative magnitude of outbreaks well and appear to capture the timing of the outbreak subsidence and resurgence between years correctly but overestimate the amount of transmission during the winter months. This might be attributable to control efforts reducing adult densities by the end of the year, and so reducing the number of infected adults still in the population. The switch from DENV-I to DENV-II, in 2020 has not appeared to change the similarity between the dynamics we predict and those observed, implying that the inter-annual variation observed is driven by environmental factors rather than the human population's resistance to dengue.

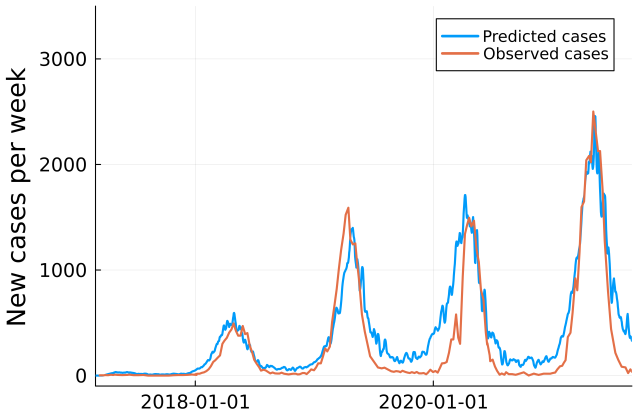

Supplementary Figure 52: A comparison of the predictions of the model (blue line) and field data (orange points and line) of the total number of dengue cases observed per week in La Réunion for the years $2017-2021$. Source data are provided as a Source Data file.

S. 2.5. Hawai'i

There was an outbreak of dengue on the island of Hawai'i between 2015 − 2016. Cases were widely distributed across the islands population centres and as there is no information available about introduction timing we follow the introduction scenario outlined for Guangzhou.

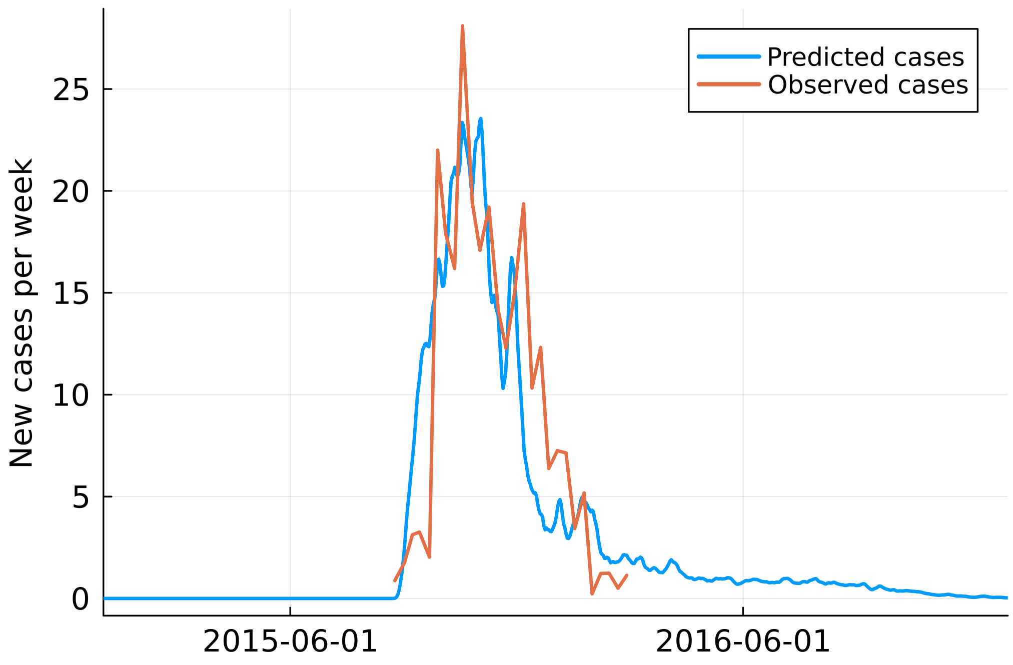

Supplementary Figure 53: A comparison of the predictions of the model (blue line) and field data (orange points and line) for the number of weekly dengue cases in Hawai'i, America. Source data are provided as a Source Data file.

S. 3. Non-plastic and unstructured models

Our model tracks the temporal evolution of the full trait structure of the population. Alternative approaches commonly make simplifying assumptions and track either mean trait over time, or do not consider trait variation at all. Here we explore the effects of these simplifying assumptions and compare their predictions to our full model. For this purpose we derive several model variants that make common simplifying assumptions about how trait interacts with environment. We compare these models over two of the outbreak locations from Figure 2 that encompass a range of different outbreak sizes. These are a small outbreak in Cagnes-sur-Mer of around 5 of dengue cases and a large outbreak in Guangzhou of 36, 342 cases. In each of these locations we were able to accurately predict both the disease and population dynamics observed in the field using the plastic model and so any substantial deviation from the models predictions will indicate a worsening of model performance.

S. 3.1. Constant wing length models

We begin considering a model that assumes that all adults express the same constant wing-length, and which we therefore refer to as the constant wing-length model. Under this assumption adult trait still varies, but only in response to the current temperature, and does not vary in response to the intensity of larval competition, nor the average temperature experienced by individuals through development. This assumption is common in mechanistic models of vector and disease dynamics and is comparable to the assumptions made by metric based R_0 models that are widely used to predict the incidence of vector borne disease (although these metric-based R_0 models often additionally assume a constant host-to-vector ratio. See Supplementary Information 4 for a comparison of our approach to these previous methods.) [45, 46]. We derive this model by setting $m = 1$ in the population dynamical model and selecting a wing-length that all individuals within the single environmental class express.

We first simulate the constant wing length model in Cagnes-sur-Mer, where the temperate environment ensures the population has a strong diapause response. The cold winter limits the number of generations of adults produced per season and this produces simple trait structures as shown in Supplementary Figure 54A. We observe in Supplementary Figure 54B that between the constant wing length models there is generally disagreement on the timing end of the active season and the abundance of adults and in general the larger the constant wing-length we select the fewer adults are produced. This occurs due to the omission of the instance of developmental plasticity that would reduce adult quality in response to high larval densities. This results in a small number of large individuals being able to maintain high larval densities that are unfavourable for development. However, besides these differences we see that in this location the population dynamics predicted by the constant wing-length models and the plastic model that includes a full trait structure are generally similar.

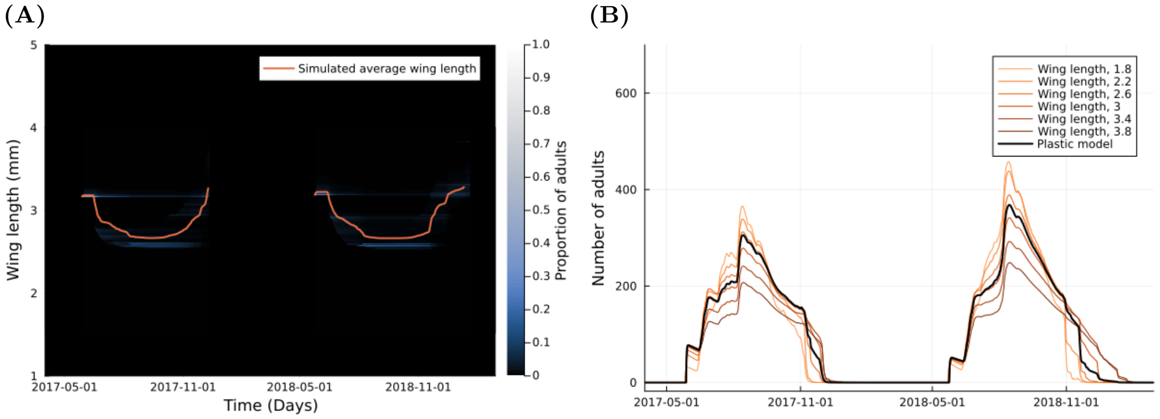

Supplementary Figure 54: **(A)** The trait dynamics of the full model in Cagnes-sur-Mer. **(B)** A comparison of the number of adults predicted by the plastic model and the constant wing-length model simulated at various wing-lengths in Cagnes-sur-Mer. Source data are provided as a Source Data file.

Consider now the dynamics of both models in Guangzhou where the plastic model predicts a much more complex trait structure (Supplementary Figure 55A). In Supplementary Figure 55B we see that although aspects of the population dynamics observed in the constant wing-length models are still observed within the dynamics of the full model, the predictions made by both are distinct. The constant wing length models disagree with each other on the ability of adults to persist through the winter, with models with low wing lengths predicting

strong seasonality in abundance, whereas models with high wing lengths predict little inter-annual variation.

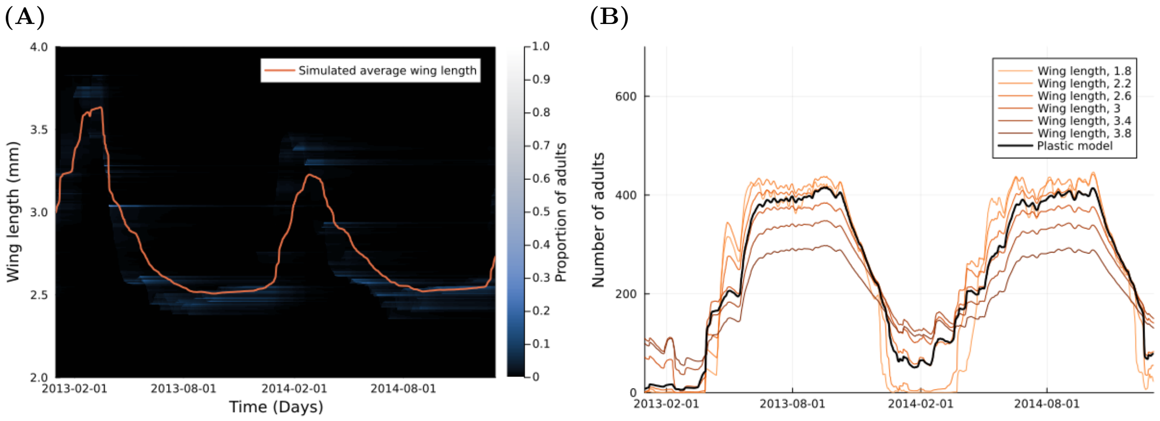

Supplementary Figure 55: (A) The trait dynamics of the full model in Guangzhou. **(B)** A comparison of the number of adults predicted by the plastic model and the non-plastic model simulated at various wing-lengths in Guangzhou. Source data are provided as a Source Data file.

These examples demonstrate that the inclusion of mechanisms of individual variation quantitatively change our predictions of population dynamics and adult abundance when compared to models that wholly omit these mechanisms. To now understand how these differences in population dynamics translate to differences in the ability of populations to transmit disease we compare the disease dynamics predicted by the constant wing lengths models to those predicted by the full model under the same introduction scenarios used in Supplementary Figure 2. In Supplementary Figure 56 we see that the differences between the reproduction number of the constant wing-length model and plastic model are comparable to the differences we previously observed in adult abundance but that outbreak size responds very differently. In Cagnes-sur-Mer, France, despite the plastic model having a reproduction number that is surpassed by many of the non-plastic models it predicts the most dengue transmission of any model considered (Supplementary Figures 56A and 56B). In Guangzhou outbreak size is highly sensitive to wing-length and between models predictions of outbreak dynamics change substantially (Supplementary Figure 56D). Furthermore, population average trait is not sufficient to predict outbreak size and the disease dynamics of the plastic model are in no way predicted by the corresponding constant wing-length model with the populations average trait value. For example, in Guangzhou the plastic model predicts a population average wing-length of 2.72 mm but the constant wing-length model where all individuals express a wing-length of 2.72 mm predicts 6 times as many cases as the plastic model. This demonstrates that disease transmission in this system is highly sensitive to vector trait and population dynamics, and that non-plastic models do not adequately account for this disparity.

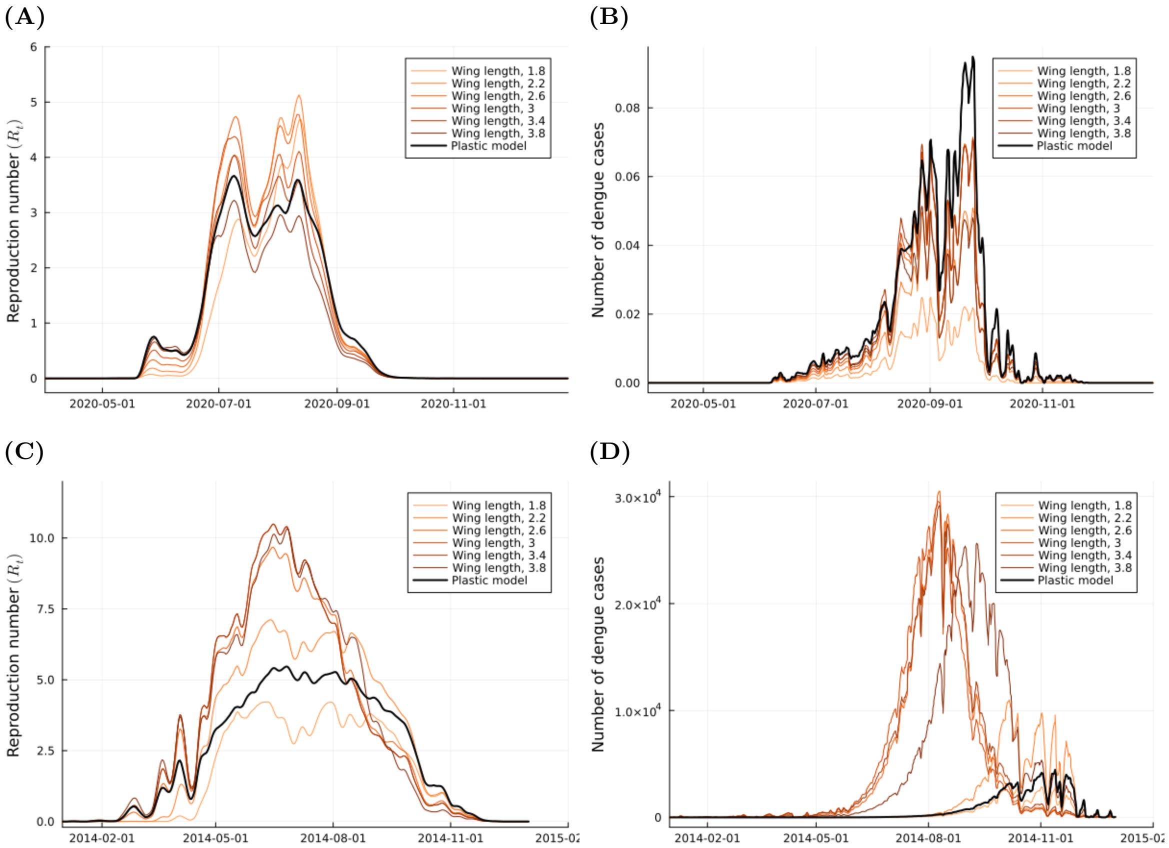

Supplementary Figure 56: A comparison of the predictions made by the full model and the constant wing length model for **(A)** R_0 in Cagnes-sur-Mer. **(B)** Number of dengue cases in Cagnes-sur-Mer. **(C)** R_0 in Guangzhou. **(D)** Number of dengue cases in Guangzhou. Source data are provided as a Source Data file.

S. 3.2. Models with instantaneously varying wing length

We have demonstrated that the inclusion of mechanisms of trait variation change our predictions, but there are many simpler ways that trait variation is commonly represented. The simplest possible way that we might decide to represent trait variation is by assuming that wing length varies instantaneously with the developmental experience of adults as they are recruited. Under this assumption although adult wing length does change in response to developmental conditions, the population average trait value does not in any way reflect the population's environmental history or trait structure. To derive a model of this type we adapt the constant wing length model such that the wing length of the single environmental class varies according to our function that uses average larval temperature and food available per larvae per day to predict the wing length of emerging adults.

When we simulate this model we see that the the instantaneous model and the plastic model produce similar population dynamics with a mismatch in abundance and trait towards the end of the season (see Supplementary Figure 57). In Supplementary Figures 57A and 57B we see that in Cagnes-sur-Mer the instantaneous model predicts similar adult dynamics and abundance to that of the plastic model despite predicting different trait dynamics. However, towards the end of the season the increase in adult trait predicted by the instantaneous model, which is induced by a brief cold spell, delays the end of the active season compared to the prediction of the full model. To see that the trait dynamics predicted by the instantaneous model variant are undesirable consider a population currently held under favourable conditions such that all adults are large. If the environment was to suddenly become unfavourable, perhaps because of a lack of precipitation inducing high larval densities, under the instantaneously varying formulation all of the large adults would shrink, as if they too had developed under these conditions. This would then cause a corresponding increase in adult mortality and individuals would begin to die off due to a spike in larval density they never experienced during development. By contrast, in the plastic model this period of high larval competition does not in any way alter the longevity of adults currently in the population.

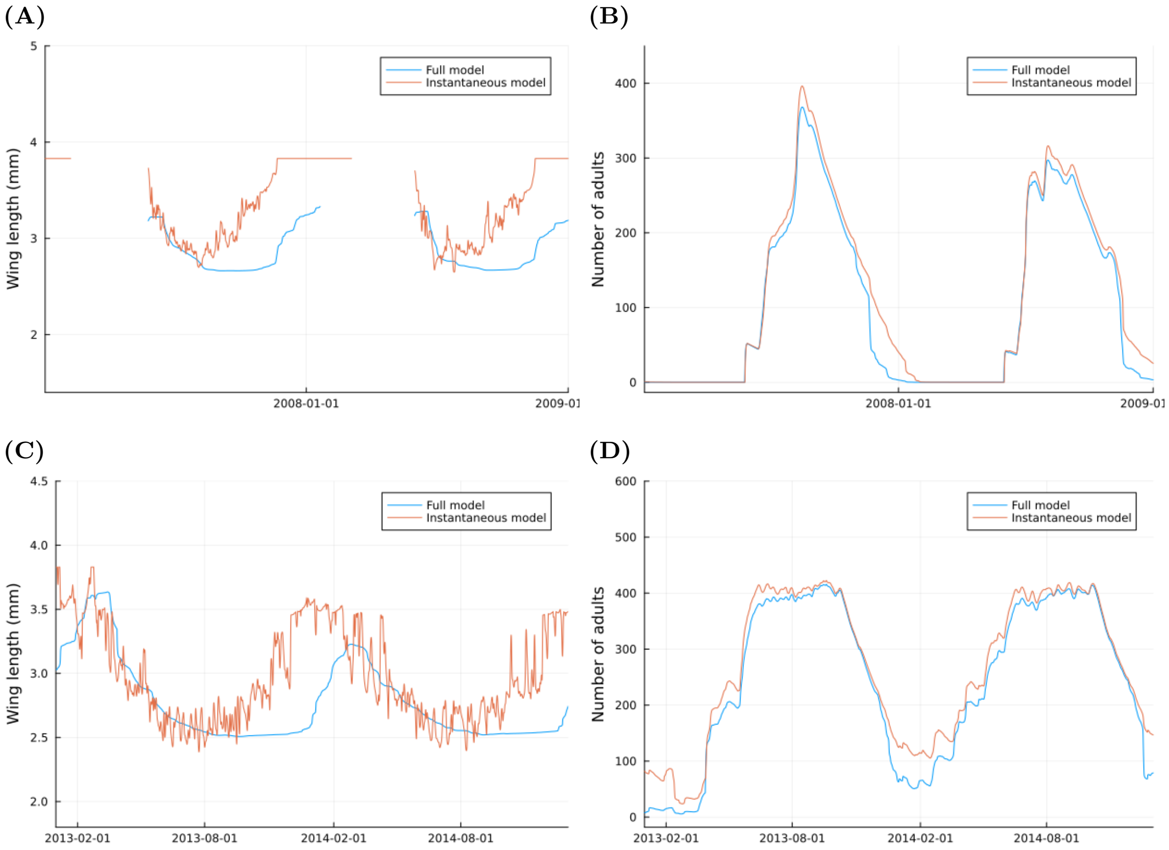

Supplementary Figure 57: A comparison of the predictions made by the full model and the model with instantaneously varying wing length model for **(A)** Wing length in Cagnes-sur-Mer. **(B)** Number of adults in Cagnes-sur-Mer. **(C)** Number of adults in Guangzhou. **(D)** Wing length in Guangzhou. Source data are provided as a Source Data file.

S. 3.3. Models with variable average wing length but without population structure

Finally, consider a model that includes all of the same mechanisms of trait variation that we consider in the plastic model but that forgoes a trait structure in favour of a population average that varies proportionally with the wing length of adults entering and leaving the population. The average wing length, denoted $w_{avg}(t)$, varies as individuals mature into the adult stage, expressing a wing-length representing their larval experience, and as adults expressing the old population average die. To track this we use differential equation that describes how the populations total wing-length, w_T, varies

$$\frac{dw_T}{dt} = R_A(t)w_l(T_{avg}(t), \bar{\alpha}(t)) - \delta_A(t)w_T(t).$$

This can then be converted to a population average wing length, $w_{avg}(t) = \frac{w_T(t)}{A(t)}$. This population average wing-length is used along with temperature in our reaction norms to allow adult traits to vary according to the current average experience of past development, and we refer to this model variant as the unstructured model. As for the previous variants we simulate this model in Cagnes-sur-Mer and Guangzhou. In Supplementary Figure 58 we see that the model with variable average wing-length but without trait-structure broadly predicts the same population dynamics as the full model (Supplementary Figure 58A and 58C). Further, the predictions of adult density made by the full model and model variant only deviate by a small amount (Supplementary Figure 58B and 58D). These similar predictions of average trait and mosquito abundance mean that any deviation between the two models in disease dynamics is directly attributable to effects of population trait structure on transmission.

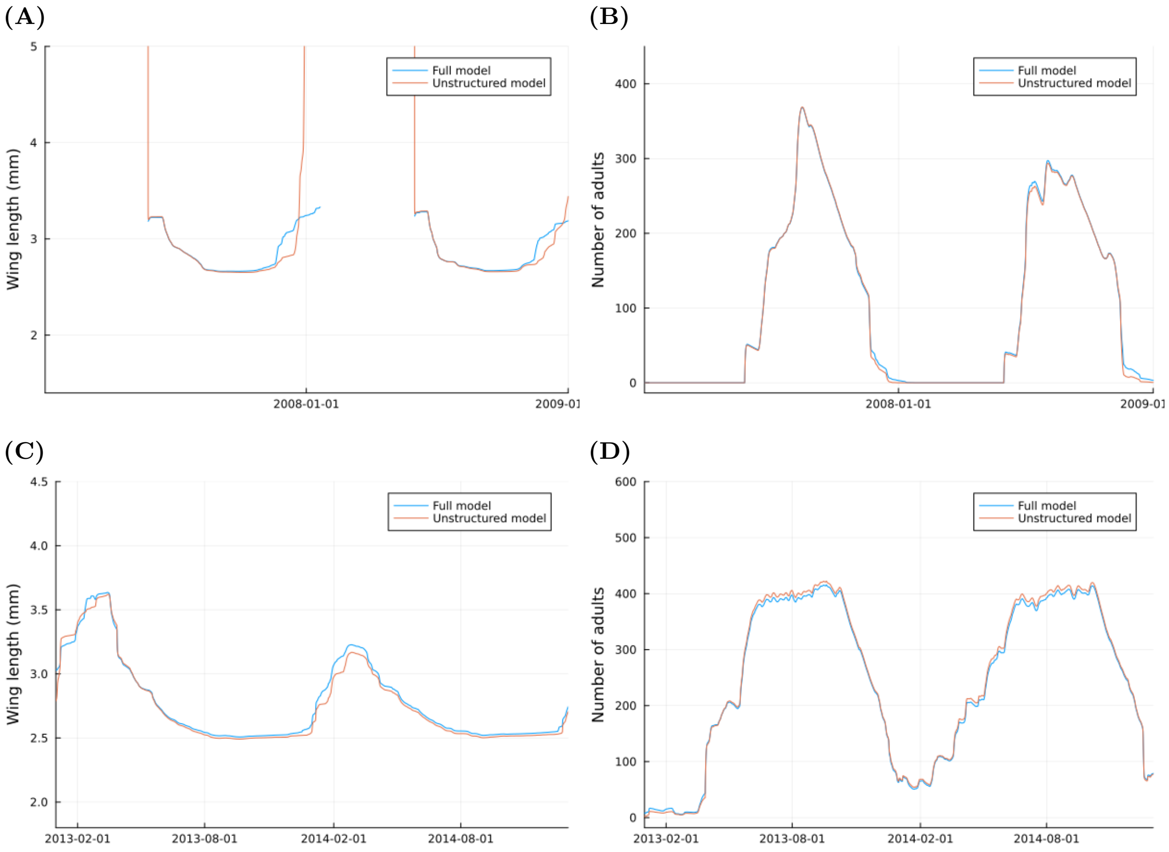

Supplementary Figure 58: A comparison of the predictions made by the plastic model and the unstructured model for **(A)** Wing length in Cagnes-sur-Mer. **(B)** Adults in Cagnes-sur-Mer. **(C)** Wing length in Guangzhou. **(D)** Adults in Guangzhou. Source data are provided as a Source Data file.

To compare the disease dynamics predicted by the unstrucutured model to those predicted by the full model we simulate both under the conditions used in the Reunion outbreak. This outbreak is chosen as a point of comparison due to it's multi-year duration which reduces the effect of our choice of introduction scenario and is therefore ideal for showing how the model's dynamics deviate from each other. We see that the constant wing length model predicts half as many instances of dengue transmission as the full model, which predicts half as many cases as the unstructured model. As the only difference between the plastic model and the unstructured

model is the presence of a trait structure we have demonstrated that the inclusion of a full trait structure in our model produces quantitatively different predictions of disease dynamics.

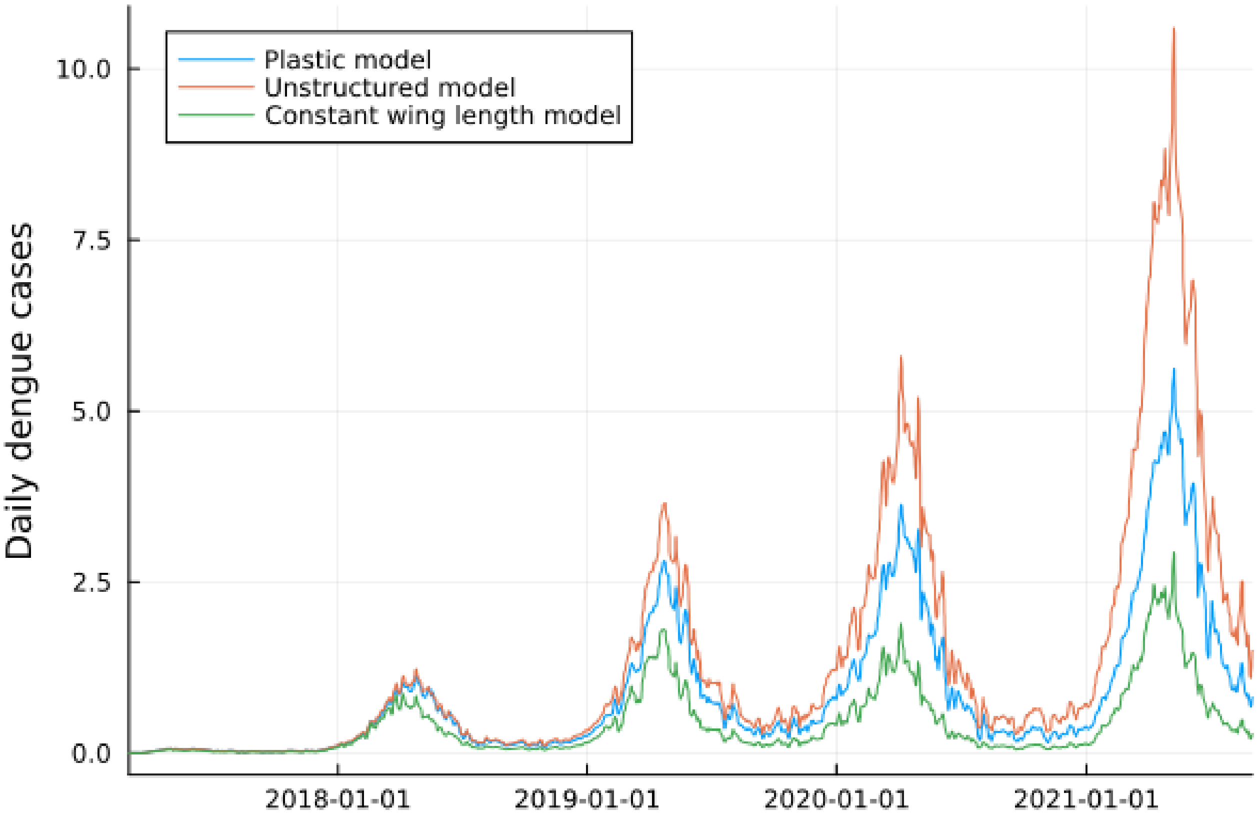

Supplementary Figure 59: A comparison of the number of dengue cases predicted in the Reunion outbreak between the full model, the unstructured model variant with dynamic average trait and a constant wing length model. Source data are provided as a Source Data file.

S. 4. The basic reproduction number, R_0

S. 4.1. Comparison to previous R_0 equations

To contextualise our predictions of the length of time regions are suitable for the autochthonous transmission of dengue by *Ae. albopictus*, we compare our predictions to those made by a previous mechanistic model by Mordecai et al. (2017). This model applies a commonly used metric to calculate a solely temperature dependent version of R_0 [46]. This model considers only the instantaneous response of each component of the transmission cycle to temperature and therefore can not consider the effect of population history on transmission dynamics. The basic reproduction number $R_0(T)$ is then defined

$$R_0(T) = \left(\frac{a(T)^2 b(T) c(T) \exp^{-\mu(t)/PDR(T)} EFD(T) p_{EA}(T) MDR(T)}{N r \mu(T)^3} \right)^{1/2} \tag{S.E. 2.}$$

In Equation S.E. 2., using the notation from Mordecai et al. $a(T)$ is the biting rate of mosquitoes, $b(T)$ is the proportion of infectious bites that successfully transmit an infection from an infected mosquito to an uninfected human, $c(T)$ is the proportion of uninfected mosquitoes that become infected upon biting an infected human. The mortality rate of adult mosquitoes is denoted $\mu(t)$, the intrinsic incubation period is $PDR(T)$, $EFD(T)$ is the number of eggs produced per female mosquito per day, $MDR(T)$ is the development rate of immature mosquitoes, N is the population density of humans, r is the rate at which humans recover from infections.

We compute the transmission length predicted by this metric over the full range we considered for the full model and observe that this non-dynamic approach predicts broad suitability for transmission of dengue over a considerably larger region than our model (Supplementary Figure 60). We observe that under this approach regions known to be at high risk of dengue outbreaks are often indistinct from those that experience little to no transmission. For example, the south of Spain where little dengue transmission has occurred appears to be at similar risk to Guangzhou, the location of the largest *Ae. albopictus* vectored outbreak of dengue.

(A)

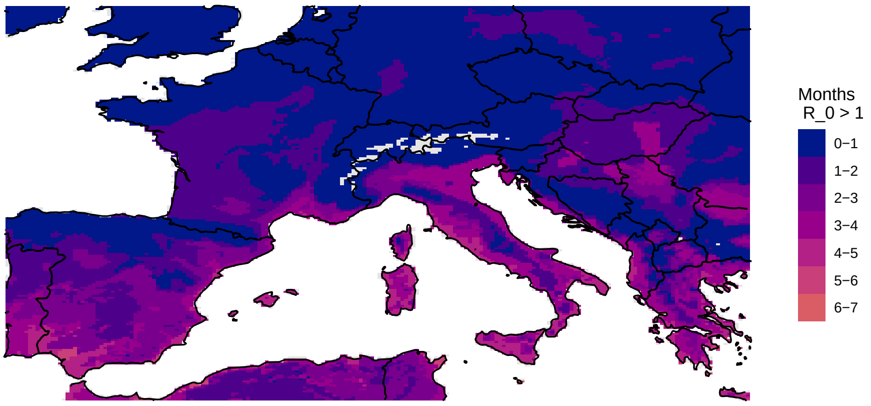

(B)

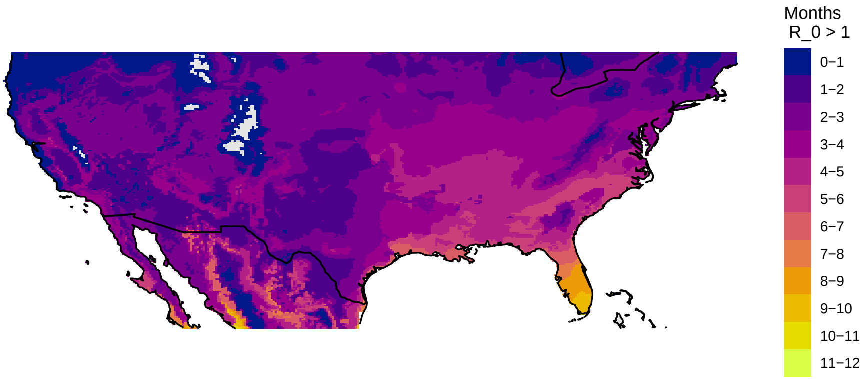

(C)

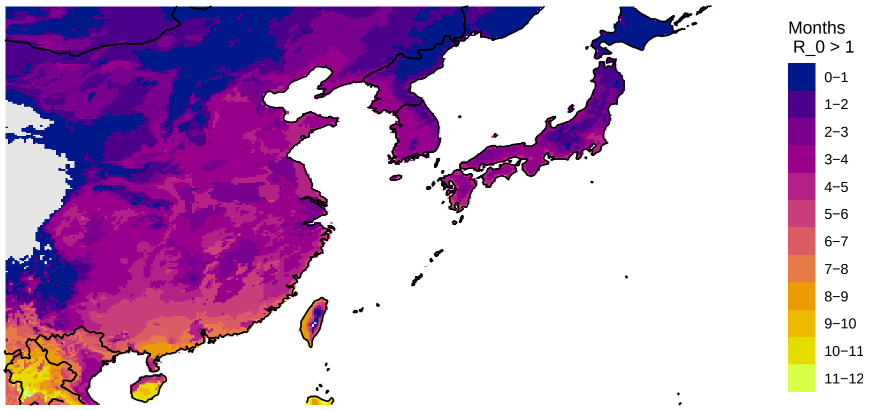

Supplementary Figure 60: The number of months for Equation S.E. 2. predicts that the autochthonous transmission of dengue by *Ae. albopictus* is possible in **(A)** Europe. **(B)** America. **(C)** Asia. Source data are provided as a Source Data file.

S. 4.2. R_t at constant temperatures

Analytically verifying the behaviour of the approximation of the time reproduction number R_t is challenging due it's complex form. For variable temperatures our expression for the time reproduction number, R_t (Equation 56), includes integrals for which we cannot find closed form solutions making it challenging to directly compare our new formulation to more standard approaches that calculate the basic reproduction number R_0. However, for a mosquito populations held at constant temperatures the adult population is at steady state, and therefore a temperature dependent expression for R_t can be derived. When this is the case our equation for R_t recovers many of the terms used in the common form of the Ross-McDonald type R_0 equation and can be expressed using the notation defined in Table 1 and throughout the rest of the manuscript by

$$R_t(T) = \left(\frac{\tau_{REC}(\frac{1}{\delta A^\star(T)}) b^2(T) h_v(T) v_h(T) 2\kappa A^\star(T) S^\star_{EIP}(T)}{H_T} \right)^{\frac{1}{2}}$$

where $A^\star(T)$ is the adult steady-state of a population held at constant temperature T, and $\delta A^\star(T)$ is the associated adult mortality rate of this population. At steady-state there is only a single trait class allowing us to dispense with the summation notation.

For a human population of 4,000 people per km^2, we plot $R_t(T)$ (Equation S. 4.2.) for a range of constant temperatures (Supplementary Figure 61A). To demonstrate that Equation S. 4.2. is accurate we simulate the model at constant temperature and without precipitation or evaporation to prevent complications arising from hydrologically induced density dependent processes. We select temperatures T_{Min} such that $R_t < 1$ for $T_{Min} - \epsilon$ and $R_t > 1$ for $T_{Min} + \epsilon$, where we choose ϵ as 0.25. If our expression for R_t is a good approximation for the time reproduction number, then when $R_t < 1$ the infection should die out and when $R_t > 1$ the number of infections should increase (although for sufficiently large time all infections will die out due to the lack of immigration or birth). Simulating the model with a single infection introduced at $t = 400$ we find that $T_{Min} = 22.00$. As we see in Supplementary Figures 61B-61C that the infection dynamics behave as expected.

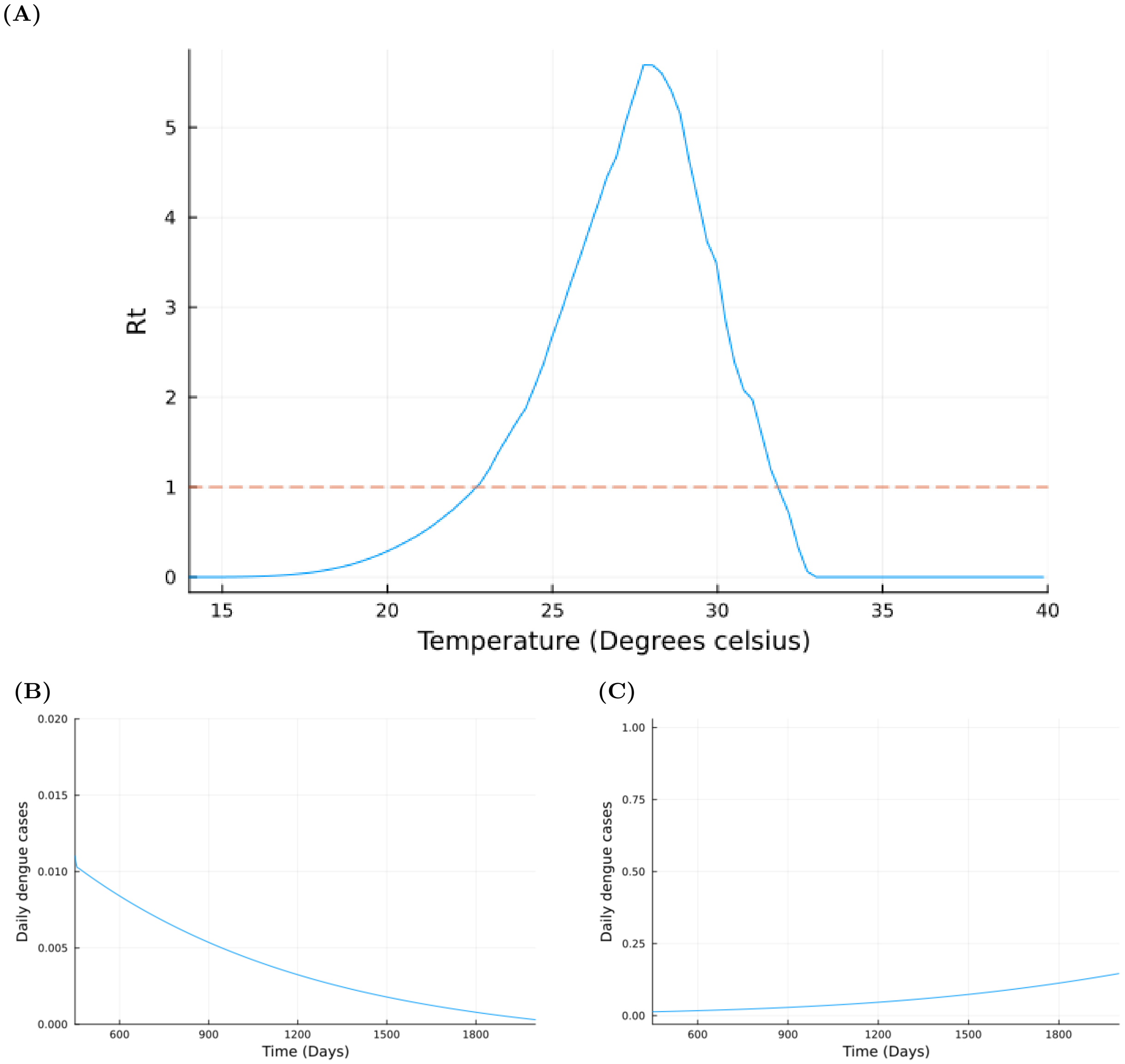

Supplementary Figure 61: (A) The value of R_t (as defined in Equation 56) when the population is held at different constant temperatures. (B) The disease dynamics when the temperature is held at $T = 22 - \epsilon$ for $\epsilon = 0.25$ giving $R_t < 1$. (C) The disease dynamics when the temperature is held at $T = 22 + \epsilon$ for $\epsilon = 0.25$ giving $R_t > 1$. Source data are provided as a Source Data file.

S. 5. Suitability maps

In addition to our predictions of the duration for which $R_t > 1$ in Figure 4 we also output other metrics of interest regarding the population, trait, and disease dynamics as predicted by our model. The outputs from our mechanistic model have to be treated with some caution as while they depend on temperature, precipitation, evaporation, and latitude, they are independent of many of other important environmental factors. For example, we do not account for humidity, habitat availability, land-use type or competitor species when we model mosquito dynamics. Instead, we make the assumption that there is always a suitable water body and sufficient adult resource, factors that are unlikely to be true in arid environments, even if there is sufficient rainfall to allow a population to persist. Humidity is important in determining adult mortality rates, and regions of low humidity or unlikely to be able to support populations of the species regardless of other environmental dynamics. For this reason we add a requirement that within the active season, as predicted by our model, that the average relative humidity is above 55%. This means that the maps that follow are more akin to an environmental suitability index than a true distribution model.

S.5.1. First day of adult activity

Predictions for the first day of the year adult activity begins. The first day of activity is defined as the first day there is more than one adult in the population.

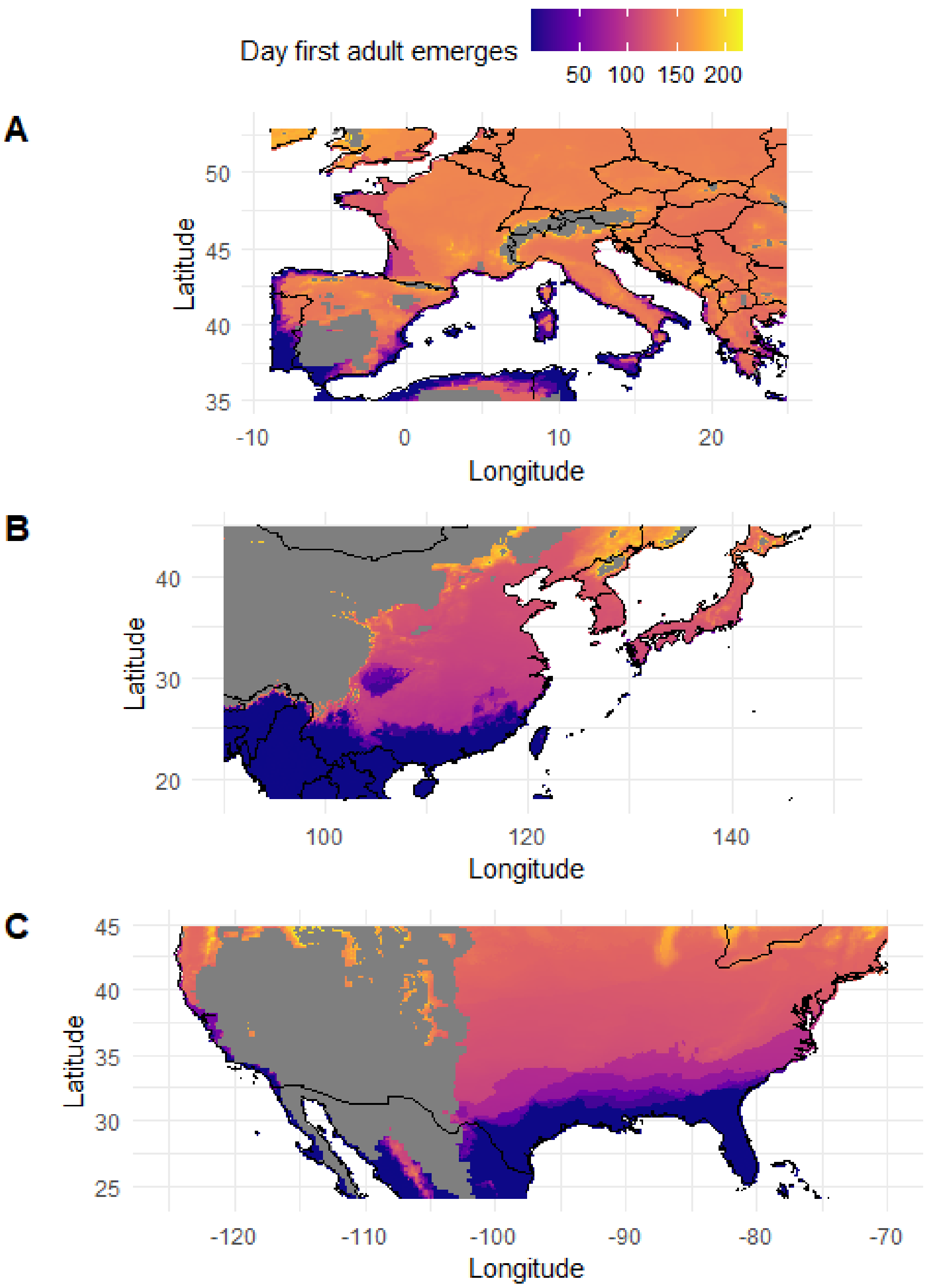

Supplementary Figure 62: The day which the first adult emerges in each location over the length of the active season. Source data are provided as a Source Data file.

S.5.2. Last day of adult activity

Predictions for the last day of the year adult activity occurs. The last day of activity is defined as the last day there is more than one adult in the population.

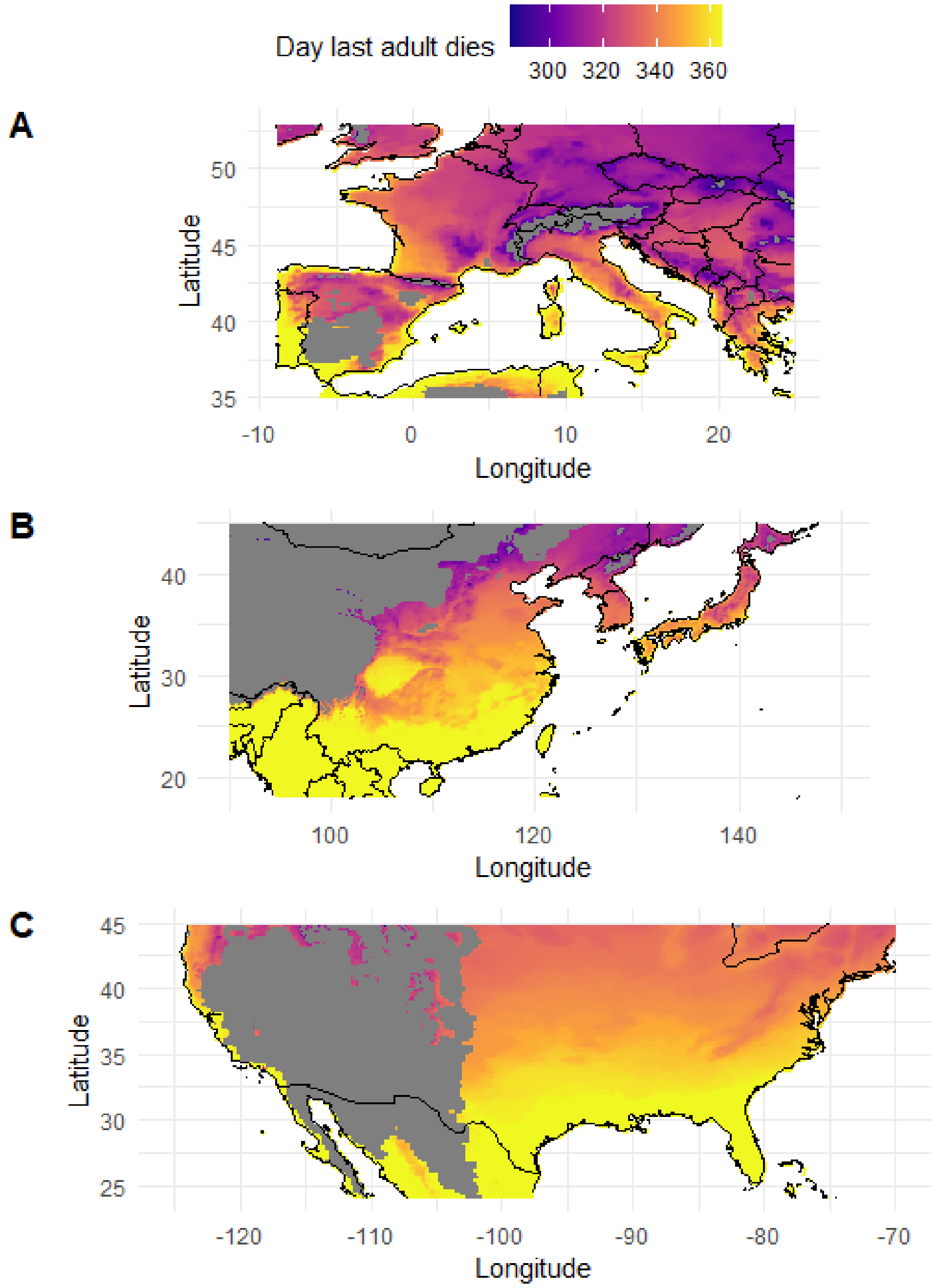

Supplementary Figure 63: The day which the last adult dies in each location over the length of the active season. Source data are provided as a Source Data file.

S.5.3. Duration of adult activity

The number of days over which adults are active each year. Defined as the difference between the first and last day of activity.

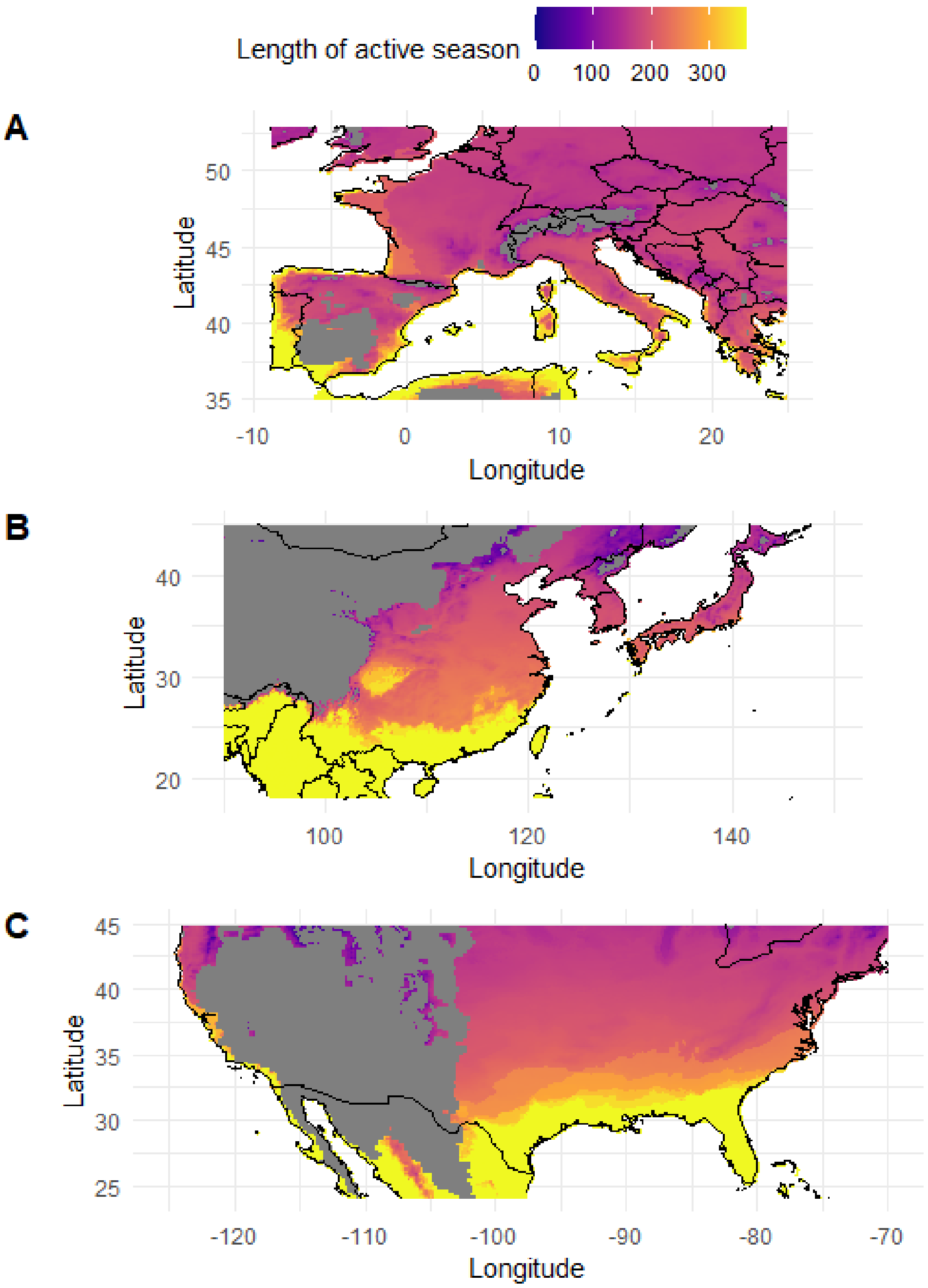

Supplementary Figure 64: The number of days for which there are adults. Source data are provided as a Source Data file.

S. 5.4. Average adult density over active season

The average density of adults over the active period. Defined as the total number of adults that are present over the year divided by the number of days over which there are active adults.

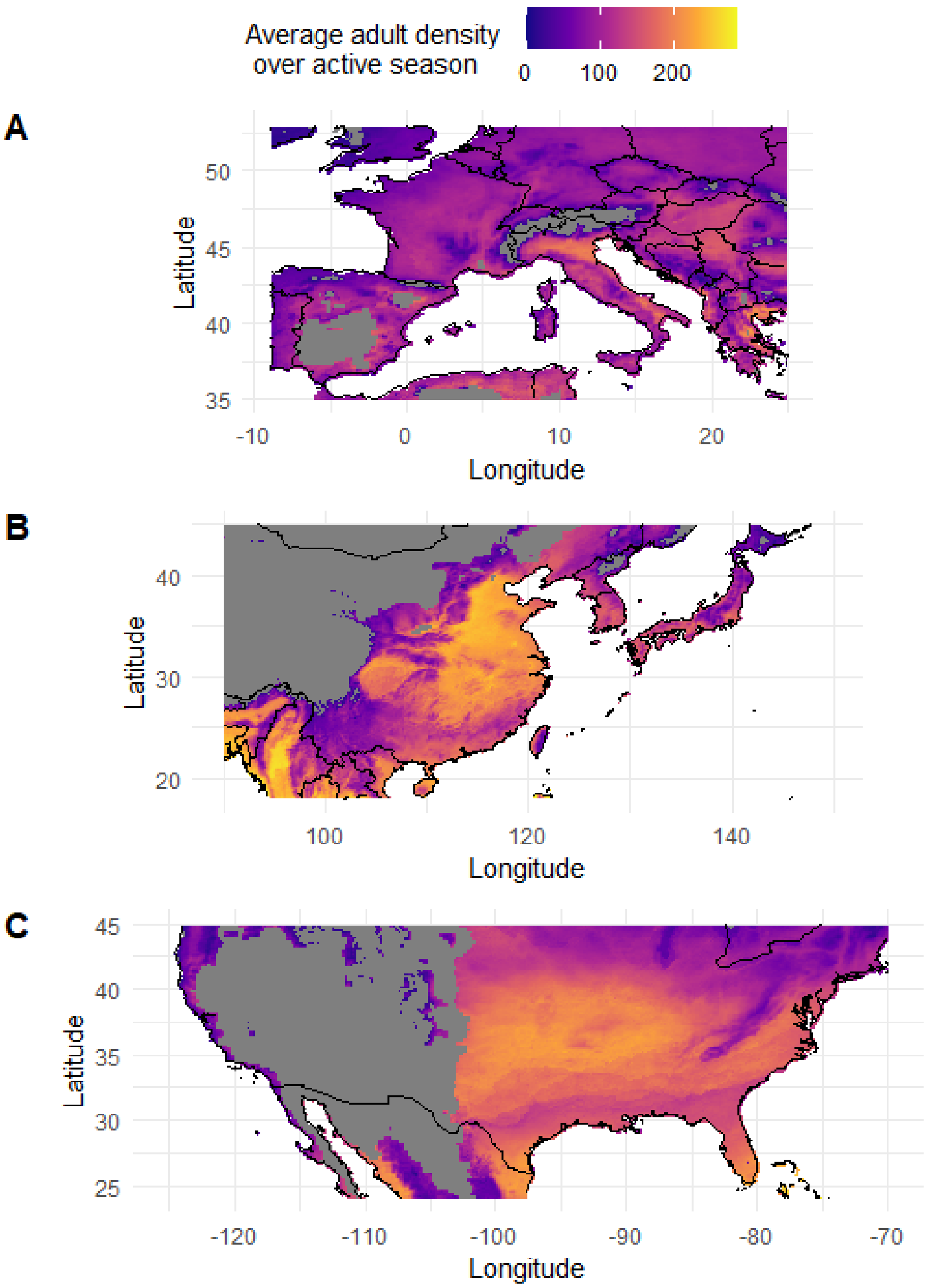

Supplementary Figure 65: The average number of adults present in each location over the length of the active season. Source data are provided as a Source Data file.

S.5.5. Average adult density over whole year

The average density of adults over the whole year. Defined as the total number of adults that are present over the year divided by the number of days in a year.

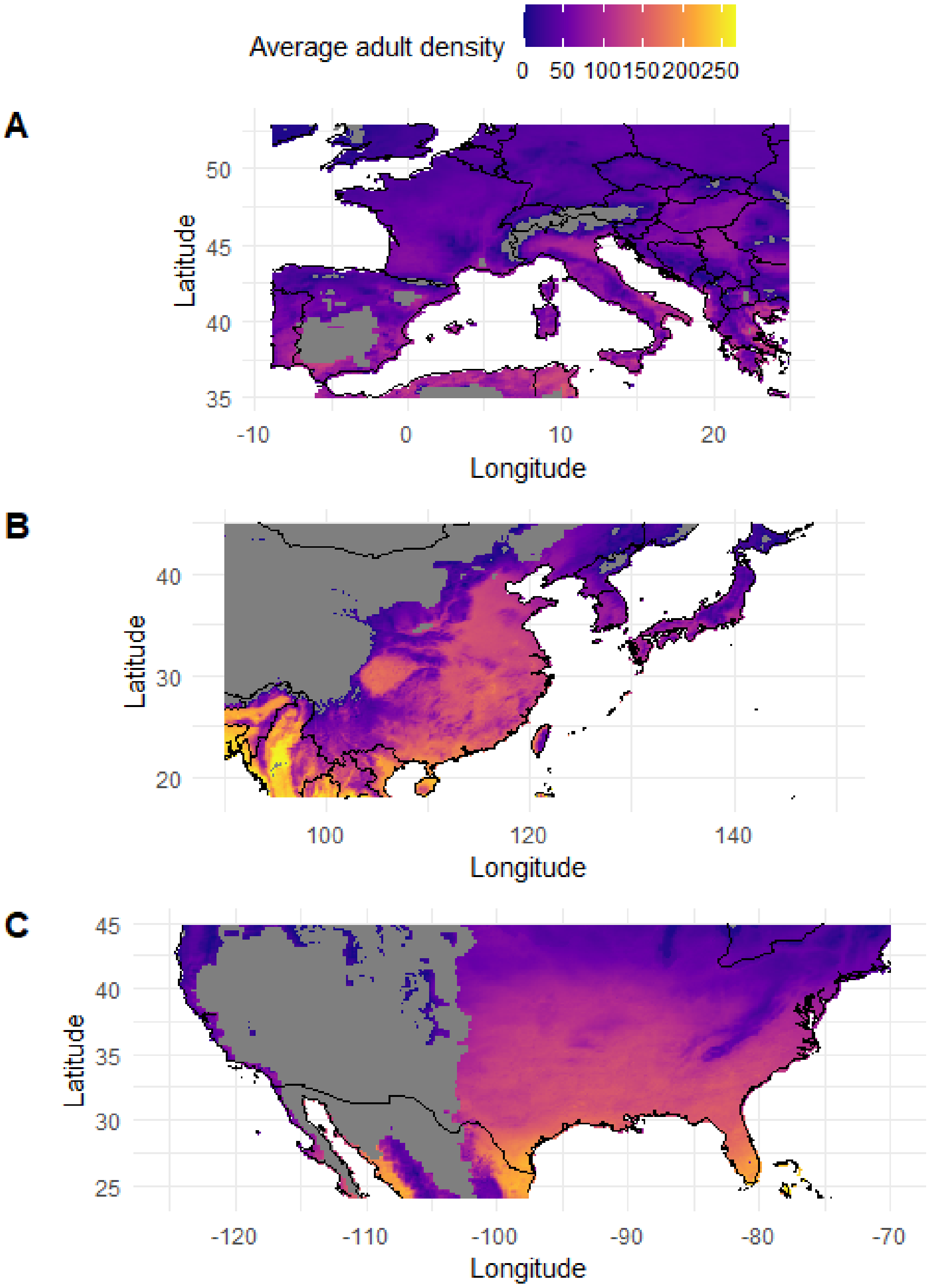

Supplementary Figure 66: The average number of adults present in each location over the length of the active season. Source data are provided as a Source Data file.

S. 5.6. Maximum adult density

The peak adult density observed over the year. Defined as the maximum adult abundance observed over the course of the year.

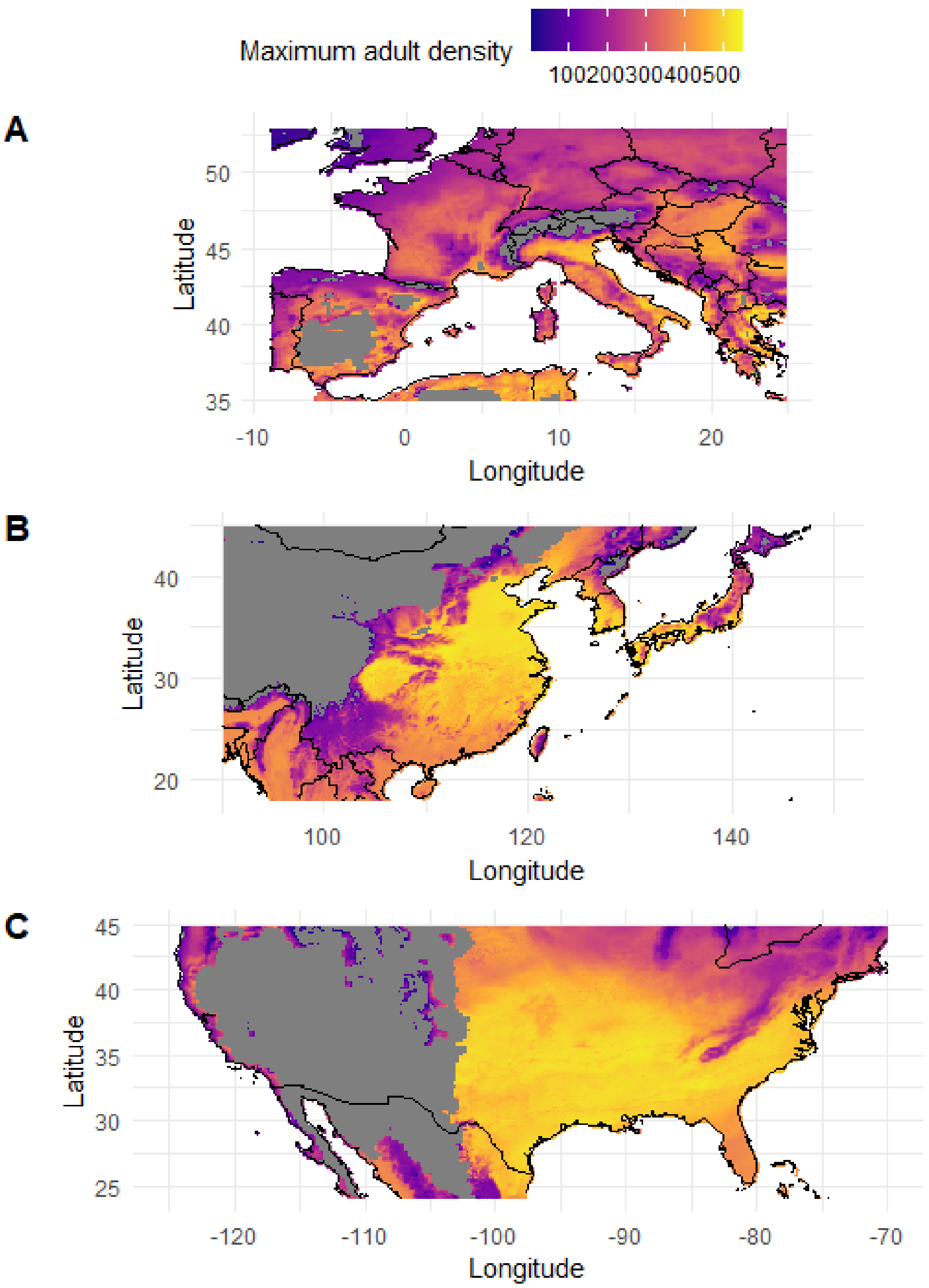

Supplementary Figure 67: The peak adult density. Source data are provided as a Source Data file.

S.5.7. Time of maximum adult density

The time that peak adult density is observed. Defined as the day that the maximum adult abundance is observed.

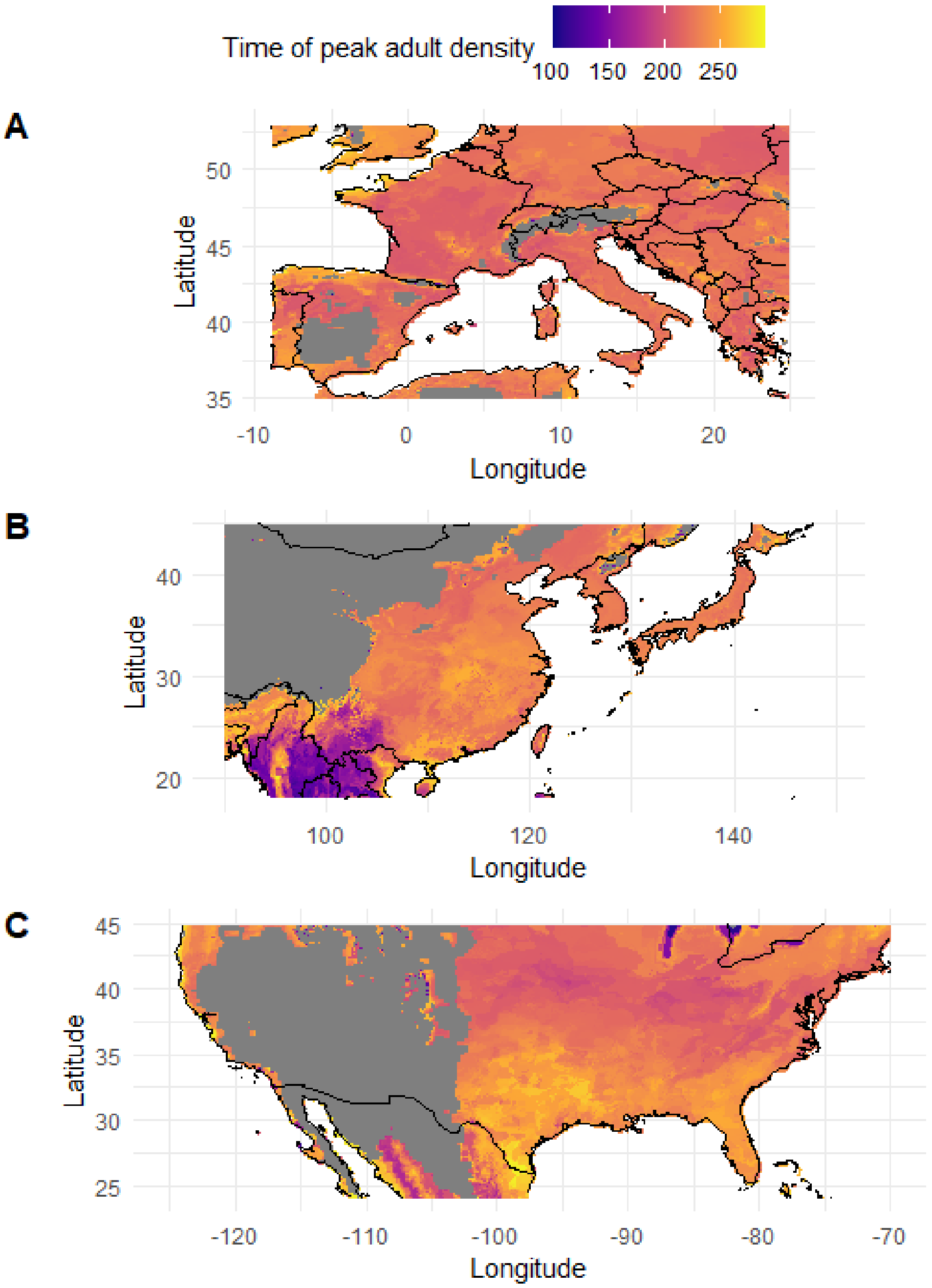

Supplementary Figure 68: The day on which peak adult density occurs. Source data are provided as a Source Data file.

S.5.8. Average trait

The average wing length of adults over the active period.

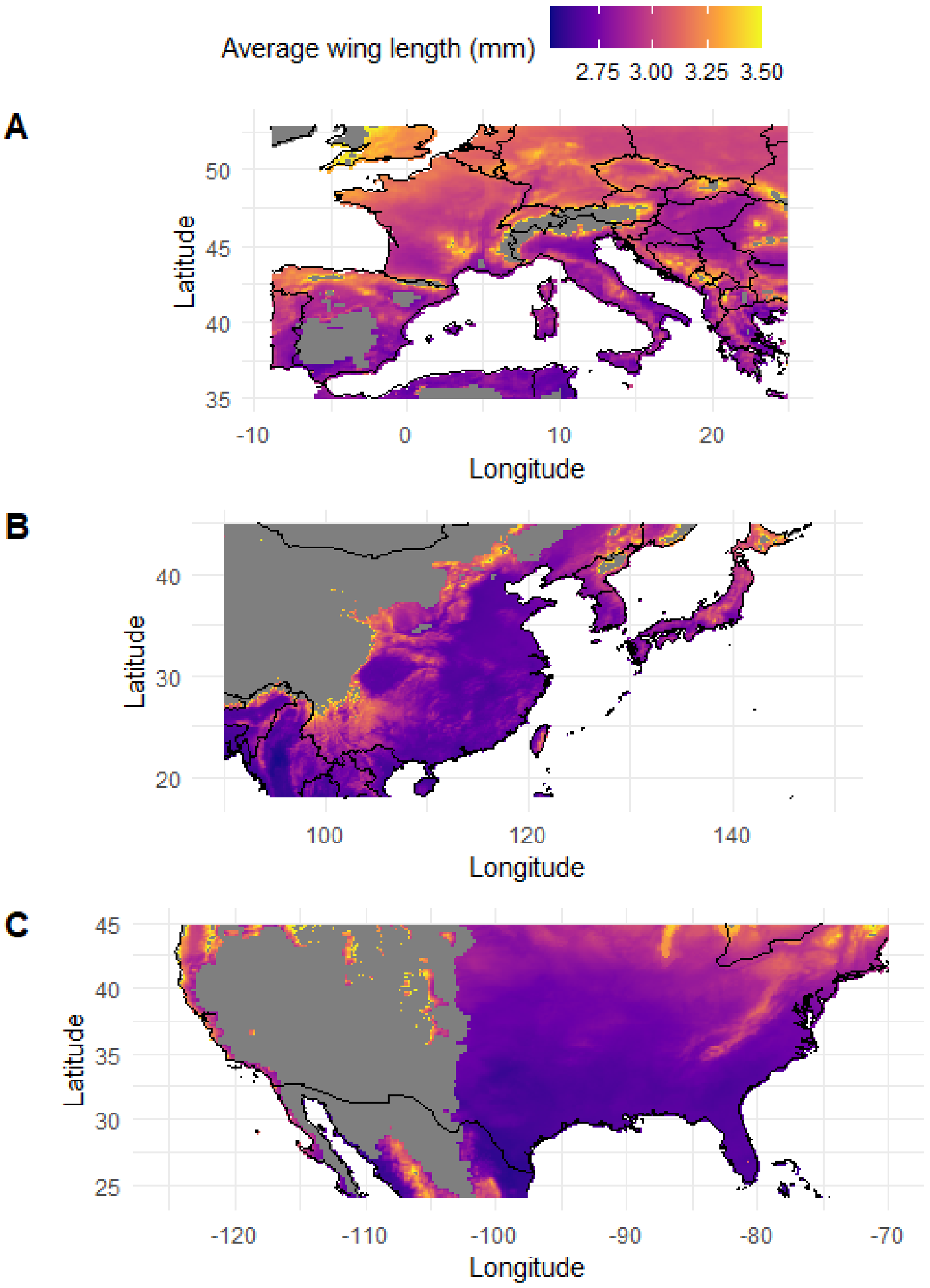

Supplementary Figure 69: The average trait value of populations. Source data are provided as a Source Data file.

S. 5.9. Total dengue cases

Predictions of the total number of dengue cases transmitted under conditions of a constant low-level introduction throughout the year.

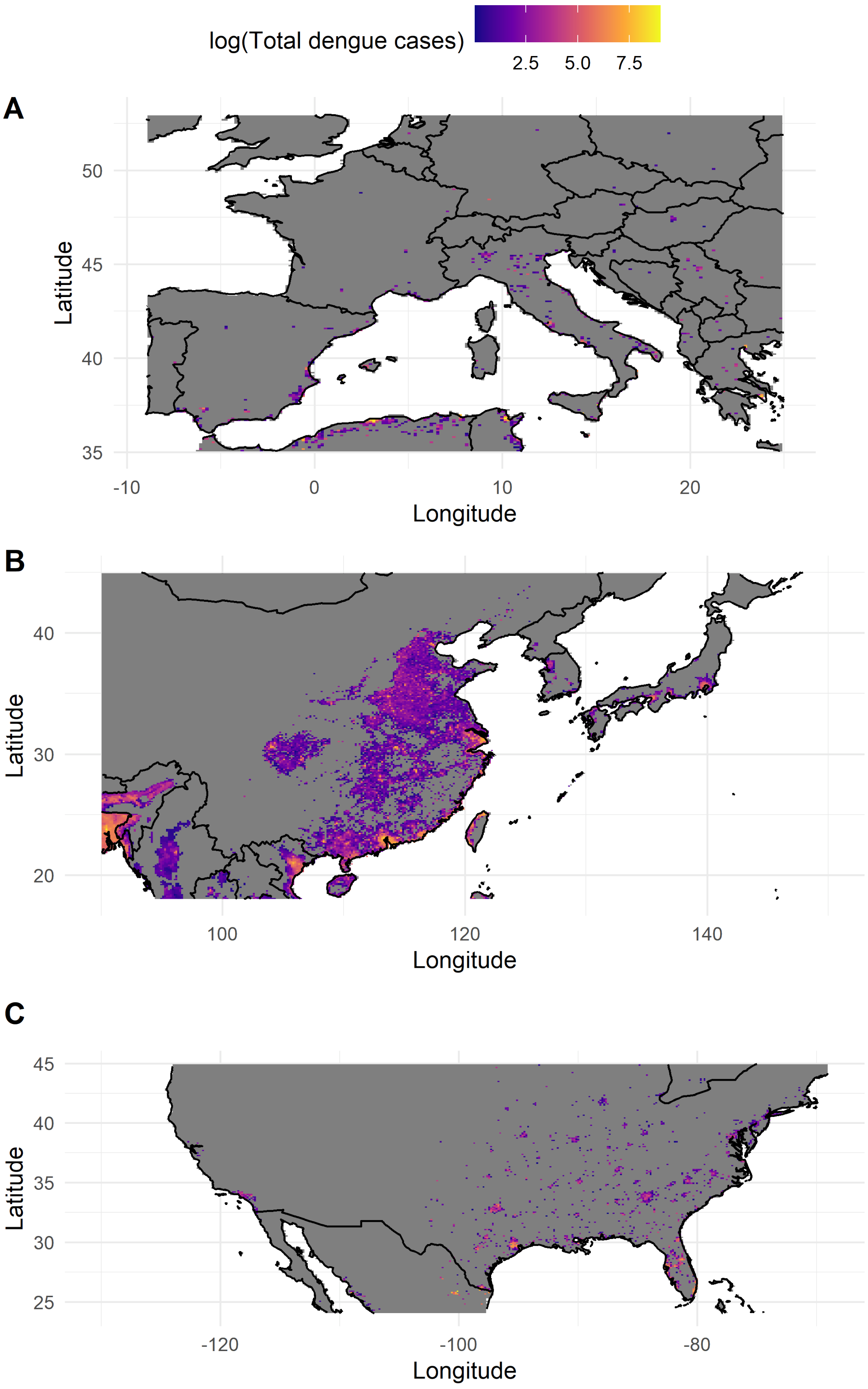

Supplementary Figure 70: The total number of dengue cases transmitted. Source data are provided as a Source Data file.

S. 5.10. First time $R_t > 1$

The first day on which $R_t > 1$.

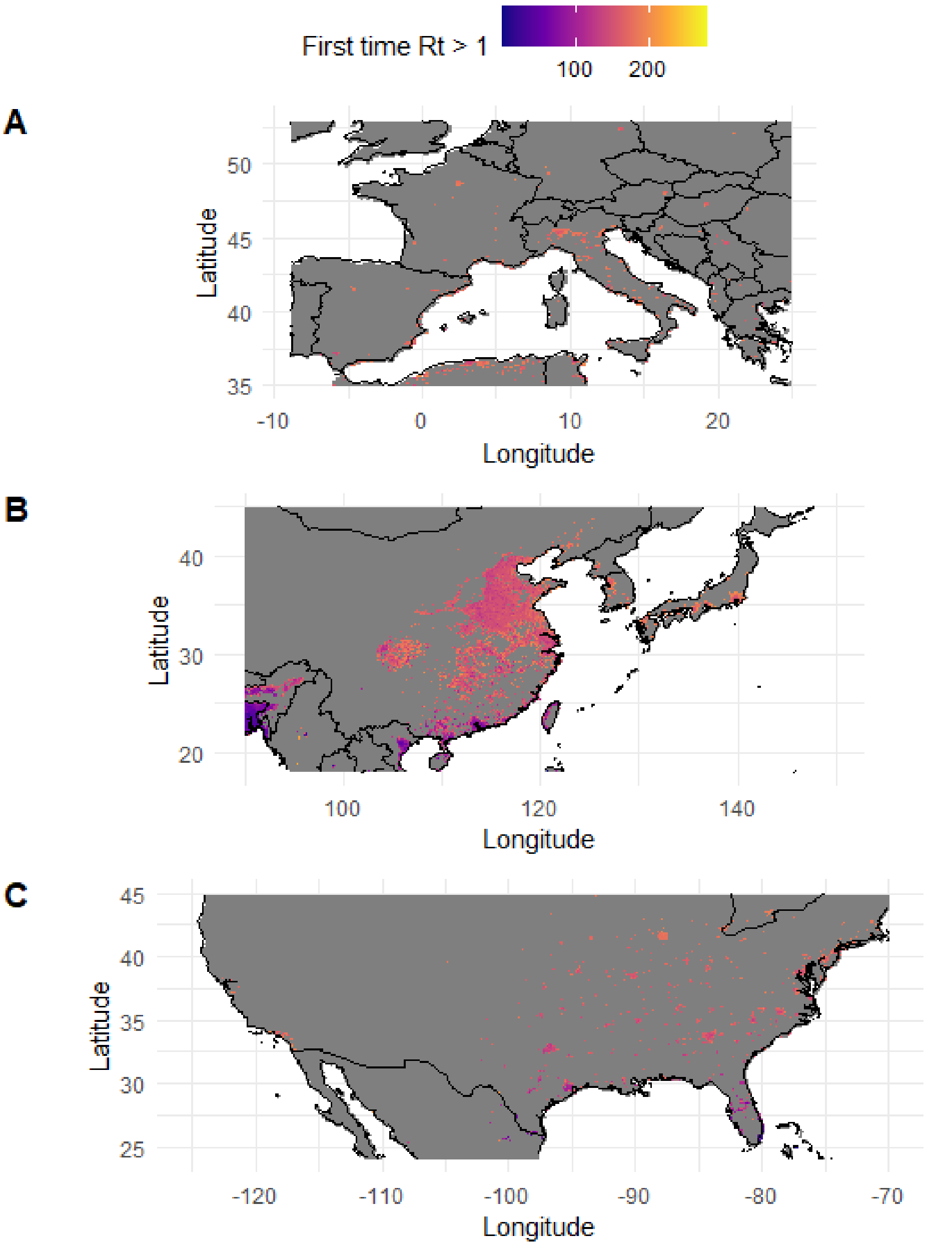

Supplementary Figure 71: The first day on which $R_t > 1$. Source data are provided as a Source Data file.

S. 5.11. Last time $R_t > 1$

The last day on which $R_t > 1$.

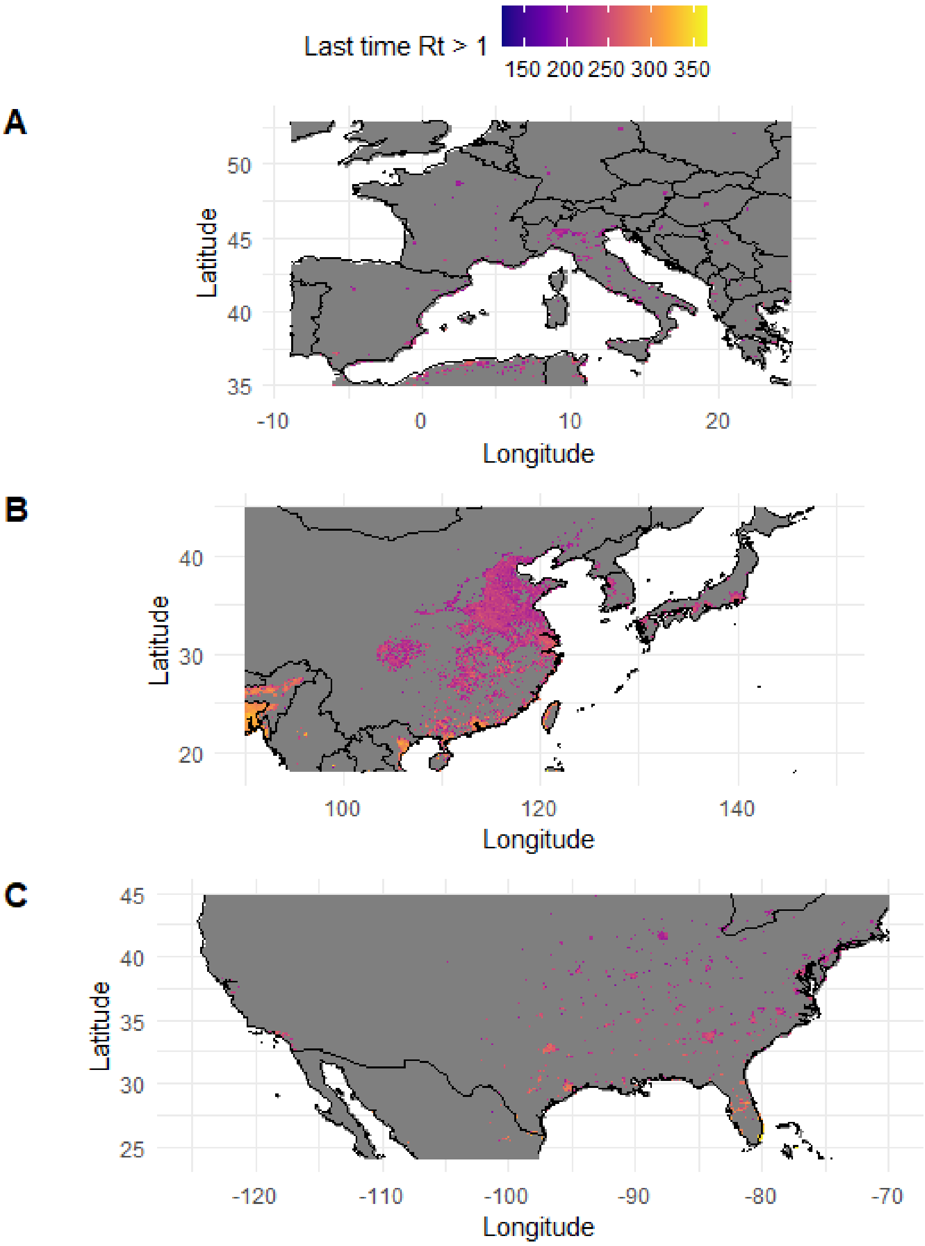

Supplementary Figure 72: The last day on which $R_t > 1$. Source data are provided as a Source Data file.

S. 5.12. Duration of time for which $R_t > 1$

Duration of time for which $R_t > 1$.

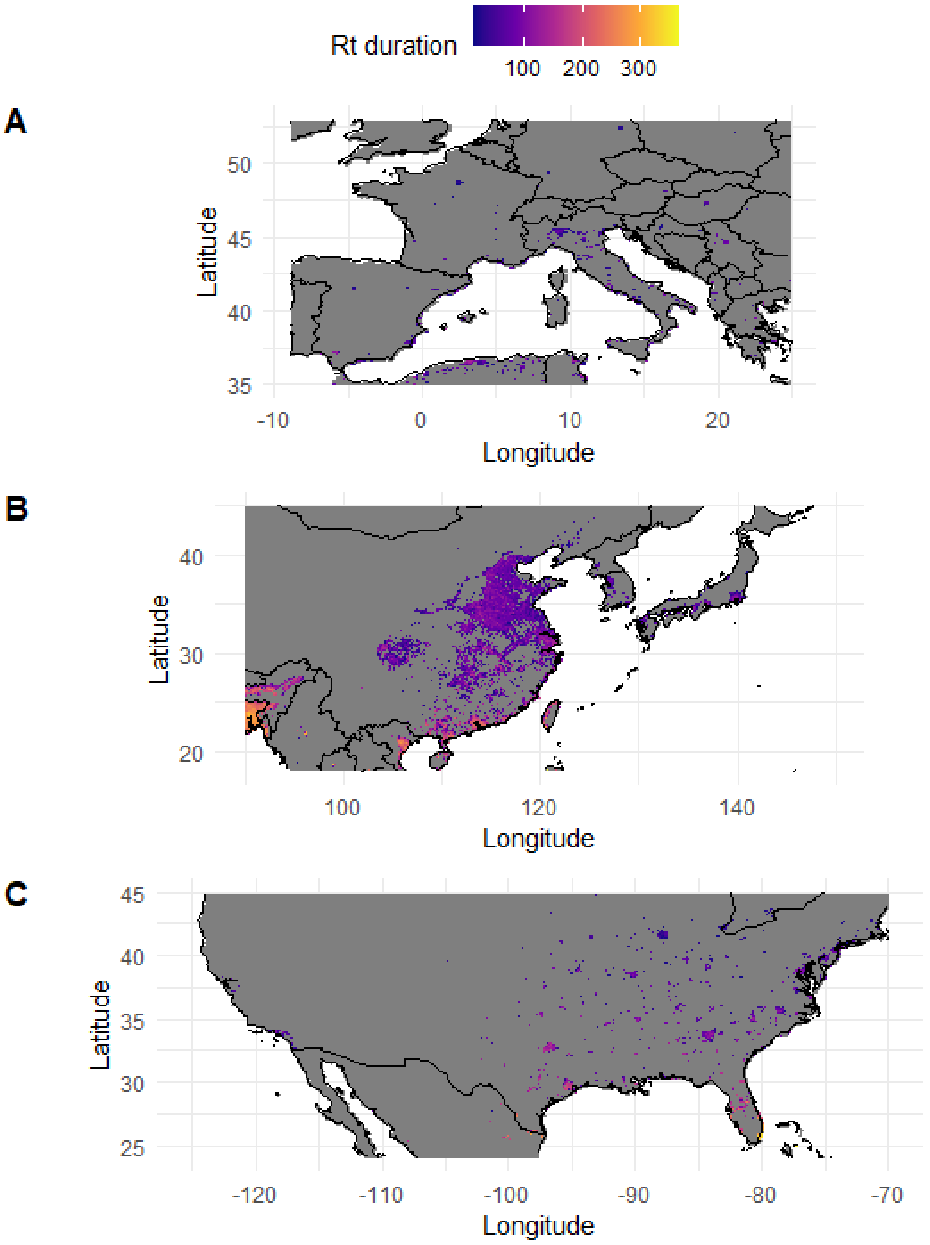

Supplementary Figure 73: Length of time for which $R_t > 1$. Source data are provided as a Source Data file.

S. 5.13. Humidity layer

The average annual relative humidity of each grid cell we consider. Areas with less then 55% humidity are excluded as too dry for the species.

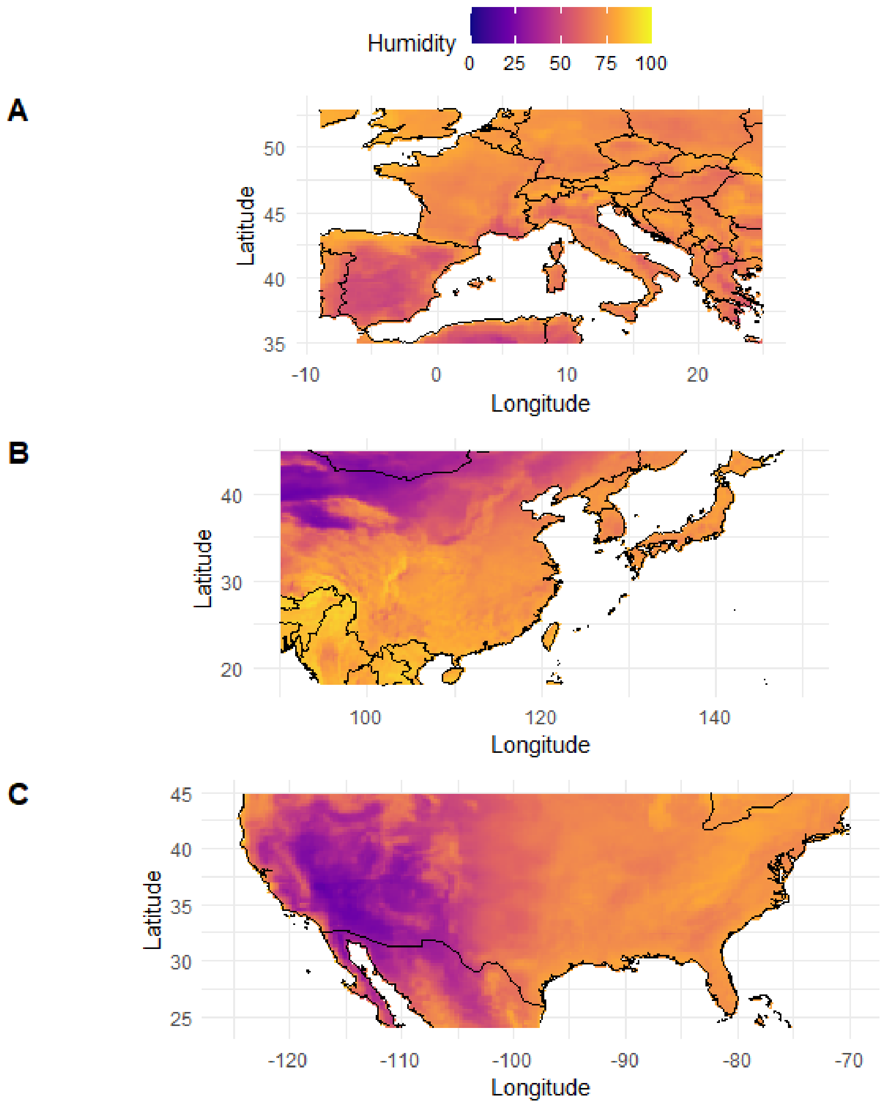

Supplementary Figure 74: The average number of adults present in each location over the length of the active season. Source data are provided as a Source Data file.

S.6. Glossary and parameter values

**Parameter**	**Interpretation**	**Values**	**Source**
**Environmental variables**			
t	Time (days)	Variable	N/A
T	Temperature (°C)	Variable	ERA5 climate reanalysis
T_{avg}	Average temperature of the larval period (°C)	Variable	ERA5 climate reanalysis
ψ	Photoperiod (hours)	Variable	N/A
ρ	Precipitation (mm per cm^{-2})	Variable	ERA5 climate reanalysis
η	Evaporation (mm per cm^{-2})	Variable	ERA5 climate reanalysis
μ	Surface area of developmental habitat (cm^2)	$130cm^2$	Tomo et al. (1982)
V	Volume of developmental habitat (ml)	500ml	Tomo et al. (1982)
W	Volume of water in developmental habitat (ml)	Variable	Calculated
l	Latitude (°)	Variable	N/A
F	Food in developmental habitat	Variable	N/A
f_d	Detritus in larval habitat	80	N/A
**Eggs**			
E_γ	Active eggs per habitat	Variable	N/A
E_Q	Quiescent eggs per habitat	Variable	N/A
E_D	Diapausing eggs per habitat	Variable	N/A
g_{E_γ}	Development rate of active eggs (day^{-1})	Variable	Various, see main text
σ_{11}	Parameter in g_{E_γ}	-0.0008256	Fitted
σ_{12}	Parameter in g_{E_γ}	0.0334072	Fitted
σ_{13}	Parameter in g_{E_γ}	-0.0557825	Fitted
τ_{E_γ}	Stage duration of active eggs (days)	Variable	Inverse of g_{E_γ}
S_{E_γ}	Through stage survival proportion of active eggs (dimensionless)	Variable	Various, see main text
σ_{21}	Parameter in S_{E_γ}	12.217	Fitted
σ_{22}	Parameter in S_{E_γ}	6.115	Fitted
σ_{23}	Parameter in S_{E_γ}	24.672	Fitted
δ_{E_γ}	Mortality rate of active eggs (day^{-1})	Variable	$-\log(S_{E_\gamma})/\tau_{E_\gamma}$
Q	Proportion of eggs produced that are quiescent (dimensionless)	Variable	Assumed
h_Q	Rate eggs are released from quiesence (day^{-1})	Variable	Assumed
D	Proportion of eggs produced that are diapausing (dimensionless)	Variable	Lacour et al. (2015)
ϕ	Critical photoperiod (hours)	Variable	Armbruster et al. (2016)
h_D	Rate eggs are released from diapause (day^{-1})	Variable	Assumed
δ_{E_D}	Mortality rate of diapausing eggs (day^{-1})	Variable	Various, see main text
**Larvae**			
L	Larvae per habitat	Variable	N/A
α	Food per larvae per day (mg day$^{-1}L^{-1}$)	Variable	N/A
$\bar{\alpha}$	Average food per larvae per day over developmental period (mg day$^{-1}L^{-1}$)	Variable	N/A
g_L	Development rate of larvae (day^{-1})	Variable	Various, see main text
τ_L	Stage duration of larvae (days)	Variable	Inverse of g_L
S_L	Through stage survival proportion of larvae (dimesnionless)	Variable	N/A
$\hat{S}_L$	Through stage survival proportion of larvae independently of density and hydrological processes (dimensionless)	Variable	Various, see main text
δ_L	Mortality rate of larvae (day^{-1})	Variable	Various, see main text
δ_d	Mortality rate of juveniles when the habitat dries out (day^{-1})	0.99	Assumed
δ_f	Mortality rate of juveniles during overspill (day^{-1})	0.2	Dieng et al. (2012)
**Pupae**			
P	Pupae per habitat	Variable	N/A
g_P	Development rate of pupae (day^{-1})	Variable	Various, see main text
σ_{31}	Parameter in g_P	$2.916e-05$	Fitted
σ_{32}	Parameter in g_P	$1.008e+01$	Fitted
σ_{33}	Parameter in g_P	$4.768e+01$	Fitted
σ_{34}	Parameter in g_P	$8.317e-01$	Fitted
τ_P	Stage duration of pupae (days)	Variable	Inverse of g_P

S_P	Through stage survival proportion of pupae (dimensionless)	Variable	N/A
$\hat{S}_P$	Through stage survival proportion of pupae independently of density and hydrological processes (dimensionless)	Variable	Various, see main text
σ_{41}	Parameter in $\hat{S}_P$	-0.0070628	Fitted
σ_{42}	Parameter in $\hat{S}_P$	0.3331028	Fitted
σ_{43}	Parameter in $\hat{S}_P$	-2.9878761	Fitted
δ_P	Mortality rate of pupae (day^{-1})	Variable	Various, see main text
**Adults**			
A_j	Adults in environmental class j per habitat	Variable	N/A
w_L	Wing length of adults (mm)	Variable	N/A
$\tau_{A_{50}}$	Time until 50% mortality of adults (days)	Fitted	Various, see main text
w_{min}	Minimum wing length of adults (mm)	1.5mm	N/A
w_{max}	Maximum wing length of adults (mm)	4mm	N/A
w_j	Transition function into environmental class j	4mm	N/A
q	Number of eggs produced per gonotrophic cycle	Variable	Blackmore and Lord (2000)
σ_{51}	Parameter in q	2.35	Fitted
σ_{52}	Parameter in q	0.69	Fitted
q_j	Rate at which an adults in environmental class j produce eggs (day^{-1})	N/A	
G	Length of gonotrophic cycle (days)	Variable	Mordecai et al. (2017)
σ_{61}	Parameter in G	$1.93e-04$	Fitted
σ_{62}	Parameter in G	10.25	Fitted
σ_{63}	Parameter in G	38.32	Fitted
δ_{A_j}	Mortality rate of adults in environmental class j (day^{-1})	Variable	Various, see main text
**Model variables**			
R_X	Recruitment of individuals into life-stage X	Variable	N/A
M_X	Maturation of individuals out of life-stage X	Variable	N/A
m	Number of environmental classes	Variable	N/A
j	Environmental class index	Variable	N/A
C	Impulse to initiate dynamics	Variable	N/A
**Dengue transmission variables**			
b	Biting rate (day^{-1})	Variable	Inverse of G
h_v	Proportion of uninfected mosquitoes that become infect after biting an infected human	Variable	Liu-Helmerson et al. (2014)
v_h	Proportion of uninfected humans that become infect after being bitten by an infected mosquito	Variable	Liu-Helmerson et al. (2014)
τ_{EIP}	Length of the extrinsic incubation period (EIP) (days)	Variable	Brady et al. (2014)
τ_{IIP}	Length of the intrinsic incubation period (IIP) (days)	4	Chan and Johansson (2012)
τ_{rec}	Time taken for infected humans to recover from infection (days)	4	Mordecai et al. (2017)
H_S	Number of susceptible humans per 4 km^2	Variable	NASA SEDAC
H_I	Number of infected humans per 4 km^2	Variable	N/A
H_R	Number of resistant humans per 4 km^2	Variable	N/A
H_b	Size of buffer population per 4 km^2	15000	Various, see main text
H_T	Total population per 4 km^2	Variable	N/A
κ	Number of larval habitats per km^2	80	Various, see main text
I_j	Number of infected mosquitoes in environmental class j per habitat	Variable	N/A
P_{EIP}	Proportion of adults in environmental class j that survive through the EIP (dimensionless)	Variable	Various, see main text

Table 1: Table of parameters and variables.

Bibliography

[1] Carrieri, M., Angelini, P., Venturelli, C., Maccagnani, B. & Bellini, R. *Aedes albopictus* (Diptera: Culicidae) Population Size Survey in the 2007 Chikungunya Outbreak Area in Italy. I. Characterization of Breeding Sites and

Evaluation of Sampling Methodologies. *Journal of Medical Entomology* (2011).

[2] Carrieri, M., Angelini, P., Venturelli, C., Maccagnani, B. & Bellini, R. *Aedes albopictus* (Diptera: Culicidae) Population size survey in the 2007 Chikungunya outbreak area in Italy. II: Estimating epidemic thresholds. *Journal of Medical Entomology* (2012).

[3] Carrieri, M. *et al.* Quality control and data validation procedure in large-scale quantitative monitoring of mosquito density: the case of *Aedes albopictus* in Emilia-Romagna region, Italy. *Pathogens and Global Health* **111**, 83–90 (2017).

[4] Toma, L., Severini, F., Di Luca, M., Bella, A. & Romi, R. Seasonal patterns of oviposition and egg hatching rate of *Aedes albopictus* in Rome. *Journal of the American Mosquito Control Association* **19**, 19–22 (2003).

[5] Manica, M. *et al.* Transmission dynamics of the ongoing chikungunya outbreak in Central Italy: From coastal areas to the metropolitan city of Rome, summer 2017. *Eurosurveillance* **22**, 18–41 (2017).

[6] Suter, T. T. *et al.* Surveillance and control of *Aedes albopictus* in the Swiss-Italian border region: differences in egg densities between intervention and non-intervention areas. *PLoS Neglected Tropical Diseases* **10**, 1–14 (2016).

[7] Bella, S., Russo, A. & Suma, P. Monitoring of *Aedes albopictus* (Skuse) (Diptera, Culicidae) in the city of Catania (Italy): seasonal dynamics and habitat preferences. *Journal of Entomological and Acarological Research* **50**, 25–30 (2018).

[8] Roiz, D., Neteler, M., Castellani, C., Arnoldi, D. & Rizzoli, A. Climatic factors driving invasion of the tiger mosquito (*Aedes albopictus*) into new areas of Trentino, Northern Italy. *PLoS ONE* **6** (2011).

[9] Marini, G. *et al.* The effect of interspecific competition on the temporal dynamics of *Aedes albopictus* and *Culex pipiens*. *Parasites and Vectors* (2017).

[10] Lencioni, V. *et al.* Multi-year dynamics of the *Aedes albopictus* occurrence in two neighbouring cities in the alps. *The European Zoological Journal* **90**, 101–112 (2023).

[11] Bonacci, T., Mazzei, A., Hristova, V. K. & Ahmad, M. A. Monitoring of *Aedes albopictus* (Diptera, Cilicidae) in Calabria, Southern Italy. *International Journal of Scientific and Engineering Research* **6**, 290–293 (2015).

[12] Lacour, G., Chanaud, L., L'Ambert, G. & Hance, T. Seasonal synchronization of diapause phases in *Aedes albopictus* (Diptera: Culicidae). *PLoS ONE* **10**, 1–16 (2015).

[13] Pajovic, I., Petrić, D., Bellini, R., Dragićević, S. & Pajović, L. *Stegomyia albopicta* skuse, 1894 (Diptera: Culicidae) on Luštica peninsula 2011-2012 (Montenegro). *Archives of Biological Sciences* **65**, 829–838 (2013).

[14] Becker, N. *et al.* Integrated control of *Aedes albopictus* in Southwest Germany supported by the Sterile Insect Technique. *Parasites and Vectors* **15**, 1–19 (2022).

[15] Goiri, F. *et al.* Progressive invasion of *Aedes albopictus* in Northern Spain in the period 2013–2018 and a possible association with the increase in insect bites. *International Journal of Environmental Research and Public Health* **17** (2020).

[16] Collantes, F. *et al.* Review of ten-years presence of *Aedes albopictus* in Spain 2004-2014: known distribution and public health concerns. *Parasites and Vectors* **8** (2015).

[17] Žitko, T. & Merdić, E. Seasonal and spatial oviposition activity of *Aedes albopictus* (Diptera: Culicidae) in Adriatic Croatia. *Journal of Medical Entomology* **51**, 760–768 (2014).

[18] Osório, H. C. *et al.* Seasonal dynamics and spatial distribution of *Aedes albopictus* (Diptera: Culicidae) in a temperate region in europe, southern portugal. *International Journal of Environmental Research and Public Health* **17**, 1–11 (2020).

[19] Petrić, M. *et al.* Seasonality and timing of peak abundance of *Aedes albopictus* in Europe: Implications to public and animal health. *Geospatial Health* **16** (2021).

[20] Willis, F. S. & Nasci, R. S. *Aedes albopictus* (Diptera: Culicidae) population density and structure in southwest Louisiana. *Journal of Medical Entomology* **31**, 594–599 (1994).

[21] Comiskey, N. M., Lowrie, R. C. & Wesson, D. M. Role of habitat components on the dynamics of *Aedes albopictus* (Diptera: Culicidae) from New Orleans. *Journal of Medical Entomology* **36**, 313–320 (1999).

[22] Álvarez Jarreta, J. *et al.* Veupathdb: the eukaryotic pathogen, vector and host bioinformatics resource center in 2023. *Nucleic Acids Research* **52** (2023).

[23] Erickson, R. A., Presley, S. M., Allen, L. J., Long, K. R. & Cox, S. B. A stage-structured, *Aedes albopictus* population model. *Ecological Modelling* **221**, 1273–1282 (2010).

[24] Armstrong, P. M., Andreadis, T. G., Shepard, J. J. & Thomas, M. C. Northern range expansion of the Asian tiger mosquito (*Aedes albopictus*): Analysis of mosquito data from Connecticut, USA. *PLoS Neglected Tropical Diseases* **11**, 1–13 (2017).

[25] Fonseca, D. M. *et al.* Area-wide management of *Aedes albopictus*. Part 2: Gauging the efficacy of traditional integrated pest control measures against urban container mosquitoes. *Pest Management Science* **69**, 1351–1361 (2013).

[26] Mundis, S. J. *et al.* Examining wing length–abundance relationships and pyrethroid resistance mutations among *Aedes albopictus* in a rapidly growing urban area with implications for mosquito surveillance and control. *International Journal of Environmental Research and Public Health* **18** (2021).

[27] Reed, E. M. X. *et al.* A statewide survey of container *Aedes* mosquitoes (Diptera: Culicidae) in North Carolina, 2016: A multiagency surveillance response to Zika using ovitraps. *Journal of Medical Entomology* **56**, 483–490 (2019).

[28] Whiteman, A. *et al.* A novel sampling method to measure socioeconomic drivers of *Aedes albopictus* distribution in Mecklenburg county, North Carolina. *International Journal of Environmental Research and Public Health* **15** (2018).

[29] Brabant, P., Lawson, D. & VectorBase. South Walton County Mosquito Control District entomological monitoring 2015 (2018). URL `https://doi.org/10.5281/zenodo.1220173`.

[30] Toma, T., Sakamoto, S. & Miyagi, I. The seasonal appearance of *Aedes albopictus* in Okinawajima, the Ryukyu archipelago, Japan. *Mosquito News* **42**, 179–183 (1982).

[31] Suzuki, A., Tsuda, Y., Takagi, M. & Wada, Y. Seasonal observation on some population attributes of *Aedes albopictus* females in Nagasaki, Japan, with emphasis on the relation between the body size and the survival. *Tropical Medicine* **35**, 91–99 (1993).

[32] Kori, M. *et al.* The 2014 autochthonous dengue fever outbreak in Tokyo: A case series study and assessment of the causes and preventive measures. *Respiratory Medicine Case Reports* **31**, 101246 (2020).

[33] Xia, D. *et al.* Photoperiodic diapause in a subtropical population of *Aedes albopictus* in Guangzhou, China: Optimized field-laboratory-based study and statistical models for comprehensive characterization. *Infectious Diseases of Poverty* **7**, 1–13 (2018).

[34] Xu, L. *et al.* Climate variation drives dengue dynamics. *Proceedings of the National Academy of Sciences of the United States of America* **114**, 113–118 (2017).

[35] Hwang, M. J. *et al.* Temporal trend of *Aedes albopictus* in local urban parks of the Republic of Korea. *Journal of Medical Entomology* **57**, 1082–1089 (2020).

[36] Gouagna, L. C. *et al.* Strategic approach, advances, and challenges in the development and application of the SIT for area-wide control of *Aedes albopictus* mosquitoes in Reunion island. *Insects* **11**, 1–24 (2020).

[37] Haramboure, M. *et al.* Modelling the control of *Aedes albopictus* mosquitoes based on sterile males release techniques in a tropical environment. *Ecological Modelling* **424**, 109002 (2020).

[38] Palisades, N. Y. N. S. D. & (SEDAC), A. C. Center for International Earth Science Information Network - CIESIN - Columbia University. 2016. Gridded Population of the World, Version 4 (GPWv4): Administrative Unit Center Points with Population Estimates. Palisades, NY: NASA Socioeconomic Data and Ap. URL `https://doi.org/10.7927/H4JW8BX5`. Accessed: 2024-05-23.

[39] ECDC. Autochthonous vectorial transmission of dengue virus in mainland EU/EEA, 2010-present (2022). URL `https://www.ecdc.europa.eu/en/all-topics-z/dengue/surveillance-and-disease-data/autochthonous-transmission-den` [illegible]. Accessed: 2024-05-23.

[40] Yue, Y. *et al.* Spatial analysis of dengue fever and exploration of its environmental and socio-economic risk factors using ordinary least squares: A case study in five districts of Guangzhou City, China, 2014. *International Journal of Infectious Diseases* **75**, 39–48 (2018).

[41] Lin, H. *et al.* Community involvement in dengue outbreak control: An integrated rigorous intervention strategy. *PLoS Neglected Tropical Diseases* **10**, 1–10 (2016).

[42] Hafsia S, Haramboure M, Wilkinson DA, Baldet T, Yemadje-Menudier L, Vincent M, Tran A, Atyame C, M. P. Overview of dengue outbreaks in the south western Indian and analysis of factors involved in the shift towards endemicity in Reunion Island: a systematic review. *PLOS Neglected Tropical Diseases* 1–16 (2022).

[43] Vincent, M. *et al.* From dengue outbreaks to endemicity: Reunion island, france, 2018 to 2021. *European Communicable Disease Bulletin* **28** (2023).

[44] Vincent, M. *et al.* From the threat to the large outbreak: Dengue on Reunion Island, 2015 to 2018. *Eurosurveillance* **24**, 1–8 (2019).

[45] Liu-Helmersson, J. *et al.* Climate change and *Aedes* vectors: 21st century projections for dengue transmission in Europe. *EBioMedicine* **7**, 267–277 (2016).

[46] Mordecai, E. A. *et al.* Detecting the impact of temperature on transmission of Zika, dengue, and chikungunya using mechanistic models. *PLoS Neglected Tropical Diseases* **11**, 1–18 (2017).
