# Supplementary material for: Role of vector phenotypic plasticity in disease transmission as illustrated by the spread of dengue virus by Aedes albopictus
Source: Nat Commun. 2024 Sep 7;15:7823. doi: 10.1038/s41467-024-52144-5 (PMC11379831; doi:10.1038/s41467-024-52144-5)
Supplement: Supplementary file 3 — Reporting Summary [file 41467_2024_52144_MOESM3_ESM.pdf]

nature portfolio

Corresponding author(s): Dominic Brass
Last updated by author(s): Aug 14, 2024

Reporting Summary

Nature Portfolio wishes to improve the reproducibility of the work that we publish. This form provides structure for consistency and transparency in reporting. For further information on Nature Portfolio policies, see our Editorial Policies and the Editorial Policy Checklist.

Statistics

For all statistical analyses, confirm that the following items are present in the figure legend, table legend, main text, or Methods section.

n/a	Confirmed	
☒	☐	The exact sample size (*n*) for each experimental group/condition, given as a discrete number and unit of measurement
☒	☐	A statement on whether measurements were taken from distinct samples or whether the same sample was measured repeatedly
☐	☒	The statistical test(s) used AND whether they are one- or two-sided<br>*Only common tests should be described solely by name; describe more complex techniques in the Methods section.*
☐	☒	A description of all covariates tested
☐	☒	A description of any assumptions or corrections, such as tests of normality and adjustment for multiple comparisons
☐	☒	A full description of the statistical parameters including central tendency (e.g. means) or other basic estimates (e.g. regression coefficient) AND variation (e.g. standard deviation) or associated estimates of uncertainty (e.g. confidence intervals)
☒	☐	For null hypothesis testing, the test statistic (e.g. *F*, *t*, *r*) with confidence intervals, effect sizes, degrees of freedom and *P* value noted<br>*Give P values as exact values whenever suitable.*
☒	☐	For Bayesian analysis, information on the choice of priors and Markov chain Monte Carlo settings
☒	☐	For hierarchical and complex designs, identification of the appropriate level for tests and full reporting of outcomes
☒	☐	Estimates of effect sizes (e.g. Cohen's *d*, Pearson's *r*), indicating how they were calculated

Our web collection on statistics for biologists contains articles on many of the points above.

Software and code

Policy information about availability of computer code

Data collection	No software was used for data collection.
Data analysis	Julia v.1.8. - Used to run all mechanistic models.<br>R v.4.3.2. - Used to run statistical analyses and to produce plots.<br>All code used to produce the results in this paper is hosted on GitHub (https://github.com/DomBrass/Aedes_DDE).

For manuscripts utilizing custom algorithms or software that are central to the research but not yet described in published literature, software must be made available to editors and reviewers. We strongly encourage code deposition in a community repository (e.g. GitHub). See the Nature Portfolio guidelines for submitting code & software for further information.

Data

Policy information about availability of data

All manuscripts must include a data availability statement. This statement should provide the following information, where applicable:

- Accession codes, unique identifiers, or web links for publicly available datasets
- A description of any restrictions on data availability
- For clinical datasets or third party data, please ensure that the statement adheres to our policy

The ERA5-land climate reanalysis dataset can be accessed from the Copernicus Climate Data Store ([\url{https://cds.climate.copernicus.eu/}]). The Gridded Population of the Word, Version (DPWv4) can be accessed through the Socioeconomic Data and Applications Center ([\url{https://sedac.ciesin.columbia.edu/}]. The

digitised life-history data and observations of field populations are provided in the Source Data file and also in the associated GitHub repository ([\url{https://github.com/DomBrass/Aedes_DDE}]).

Research involving human participants, their data, or biological material

Policy information about studies with human participants or human data. See also policy information about sex, gender (identity/presentation), and sexual orientation and race, ethnicity and racism.

Reporting on sex and gender	N/A
Reporting on race, ethnicity, or other socially relevant groupings	N/A
Population characteristics	N/A
Recruitment	N/A
Ethics oversight	N/A

Note that full information on the approval of the study protocol must also be provided in the manuscript.

Field-specific reporting

Please select the one below that is the best fit for your research. If you are not sure, read the appropriate sections before making your selection.

☐ Life sciences ☐ Behavioural & social sciences ☒ Ecological, evolutionary & environmental sciences

For a reference copy of the document with all sections, see nature.com/documents/nr-reporting-summary-flat.pdf

Ecological, evolutionary & environmental sciences study design

All studies must disclose on these points even when the disclosure is negative.

Study description	We derive, parameterise and simulate a mathematical model that predicts the population and trait dynamics of the invasive mosquito Aedes albopictus and the ability of these populations to transmit dengue. The model is parameterised using data from laboratory studies and it's ability to predict mosquito population dynamics independently validated against field data from across the species global range. Our predictions of dengue dynamics are compared to observed outbreaks and we examine the role of phenotypic plasticity in driving disease transmission.
Research sample	N/A
Sampling strategy	N/A
Data collection	Dominic Brass gathered data regarding disease outbreaks, field data from mosquito populations, and life-history data from published sources.
Timing and spatial scale	Data regarding disease outbreaks, field data from mosquito populations, and life-history data were gathered from published sources in 2021-2022. Model development and writing the paper took two years.
Data exclusions	No data was excluded.
Reproducibility	To ensure the work is reproducible we use open source programming languages, attribute all datasets used, and have hosted the code used to run the analyses on GitHub.
Randomization	N/A
Blinding	N/A

Did the study involve field work? ☐ Yes ☒ No

Reporting for specific materials, systems and methods

We require information from authors about some types of materials, experimental systems and methods used in many studies. Here, indicate whether each material, system or method listed is relevant to your study. If you are not sure if a list item applies to your research, read the appropriate section before selecting a response.

Materials & experimental systems

n/a	Involved in the study
☒	☐ Antibodies
☒	☐ Eukaryotic cell lines
☒	☐ Palaeontology and archaeology
☒	☐ Animals and other organisms
☒	☐ Clinical data
☒	☐ Dual use research of concern
☒	☐ Plants

Methods

n/a	Involved in the study
☒	☐ ChIP-seq
☒	☐ Flow cytometry
☒	☐ MRI-based neuroimaging

Plants

Seed stocks	N/A
Novel plant genotypes	N/A
Authentication	N/A
